# Discovery of TIGIT inhibitors based on DEL and machine learning

**DOI:** 10.3389/fchem.2022.982539

**Published:** 2022-07-26

**Authors:** Feng Xiong, Mingao Yu, Honggui Xu, Zhenmin Zhong, Zhenwei Li, Yuhan Guo, Tianyuan Zhang, Zhixuan Zeng, Feng Jin, Xun He

**Affiliations:** ^1^ Shenzhen Innovation Center for Small Molecule Drug Discovery Co., Ltd., Shenzhen, China; ^2^ Shenzhen NewDEL Biotech Co., Ltd., Shenzhen, China

**Keywords:** DNA-encoded library, machine learning, protein-protein interaction, TIGIT, anti-tumor

## Abstract

Drug discovery has entered a new period of vigorous development with advanced technologies such as DNA-encoded library (DEL) and artificial intelligence (AI). The previous DEL-AI combination has been successfully applied in the drug discovery of classical kinase and receptor targets mainly based on the known scaffold. So far, there is no report of the DEL-AI combination on inhibitors targeting protein-protein interaction, including those undruggable targets with few or unknown active scaffolds. Here, we applied DEL technology on the T cell immunoglobulin and ITIM domain (TIGIT) target, resulting in the unique hit compound **1** (IC_50_ = 20.7 μM). Based on the screening data from DEL and hit derivatives **a1**-**a34**, a machine learning (ML) modeling process was established to address the challenge of poor sample distribution uniformity, which is also frequently encountered in DEL screening on new targets. In the end, the established ML model achieved a satisfactory hit rate of about 75% for derivatives in a high-scored area.

## 1 Introduction

One of the main breakthroughs to improve the success rate of new drug development is applying new technologies for hit discovery and optimization, such as DNA-encoded library (DEL) (Brenner and Lerner, 1992; Franzini et al., 2014; Johnson, 2018) and artificial intelligence (AI) (Smalley, 2017) et al. Thanks to the rapid growth of computing power and the availability of large datasets, AI is being used more and more frequently in the field of drug development. Among them, the hit discovery and optimization of lead compounds are one of the fastest-developing fields, generating massive amounts of high-quality compound datasets (Tetko et al., 2016). The most well-known AI-driven drug development (AIDD) case is the DDR1 inhibitors discovery by Zhavoronkov et al. They claimed to have discovered a highly active, selective, and bioavailable inhibitor of DDR1 within 21 days through AI-aided drug design (Zhavoronkov et al., 2019). However, the active inhibitors they finally obtained were too structurally like known DDR1 inhibitors, which raised some doubts that it was indeed a fast-follow drug development (Walters and Murcko, 2020). The main reason is that many active skeletons of DDR1 inhibitors have been reported. The built AI model is based on known data for skeleton modification, making it difficult to break through the constraints of existing skeletons and produce the first-in-class drugs with a novel skeleton. Therefore, it is still unknown how long the AIDD development will be widely and successfully applied in first-in-class drugs discovery.

For medicinal chemistry, traditional structural optimization mainly relies on medicinal chemists to analyze the structure-activity relationship (SAR) through continuous cycle of chemical synthesis-bioactivity tests. However, this approach is often time-consuming and varies from target to target. In this way, it is still difficult for a bioactive compound to reach IC_50_ value of nanomolar from micromolar range in a short period. Fortunately, this limit of efficiency has been substantially improved by AIDD (Griffen et al., 2020). One of the most used functions of AIDD is to improve this efficiency through rapid model iterations significantly and finally provide fast-follow drug candidates. Therefore, integrating AIDD with other technologies which have the potential to discover first-in-class hit compounds will be valuable while may be accompanied by challenges.

**FIGURE 1:**
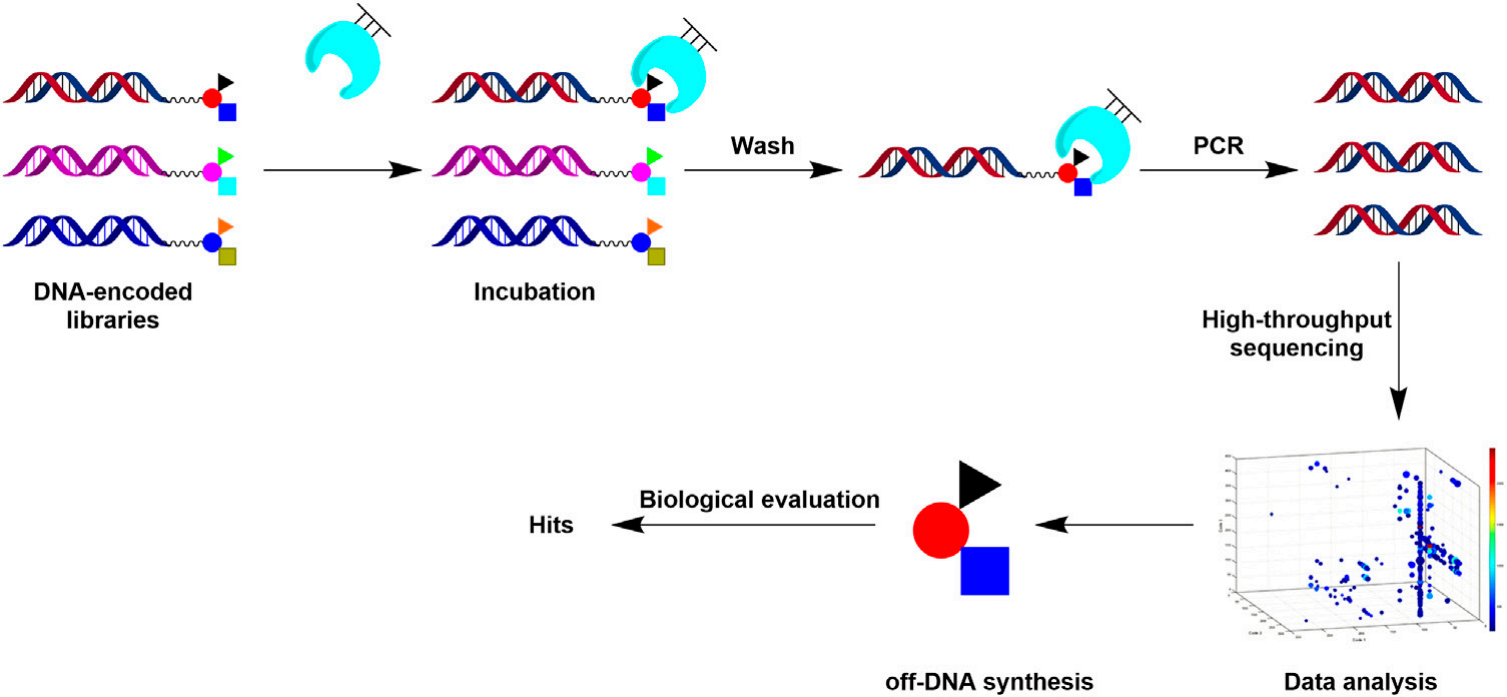
Workflow for DEL screening using immobilized proteins.

DEL is achieved through combinatorial chemistry and DNA-encoding techniques. With library modularity, DELs can be built in a time-saving and labor-saving way. This technology can construct and screen unprecedented scale combinatorial compound libraries (hundreds of billions scale) and discover numerous high-affinity ligands with high efficiency and low cost through protein target affinity screening and high-throughput sequencing and decoding (Buller et al., 2010; Kalliokoski, 2015). (Figure 1) (Goodnow et al., 2017). DEL can be used to create compound libraries with higher molecular weight. Empirically, such DEL libraries appear well suited for discovering ligands for protein-protein interaction (PPI) targets, which are increasingly needed for hits. In contrast, kinase or typical receptor are other target classes often with available hit information through traditional HTS and similar approaches based on existed skeletons. Therefore, performing DEL on such targets may not be as pressing (Goodnow et al., 2017). To our knowledge, the number of small-molecule inhibitors identified by DEL screening of PPI targets in the past decade is relatively few, mainly including LFA-1, TEAD, Bcl-xL, and IL-2. (Buller et al., 2009; Kollmann et al., 2014; Kunig et al., 2020; Gironda-Martínez et al., 2021; Wang et al., 2022). On the other hand, the PPI targets is much challenging for DEL screening, due to the lack of information on existing scaffolds from other sources. Generally, PPI targets contain large and flat binding surfaces which hinder small molecules to bind strongly. Therefore, DEL’s application on the PPI target will probably generate a few or no hits. Every hit compound for such a target is much more unique and valuable. Once a hit compound with an active scaffold is obtained, developing a first-in-class drug candidate against PPI targets is much more promising.

Although machine learning (ML), as a branch of AI, has been applied to multiple areas of drug discovery, to our knowledge, cases of DEL combined with ML have not been reported until recently (McCloskey et al., 2020; Lim et al., 2022). McCloskey et al. successfully performed ML modeling using data obtained from DEL screening for targets including sEH (a hydrolase), ERα (a nuclear receptor), and c-KIT (a kinase). Another example came from Lim et al., who screened carbonic anhydrase (CAIX), soluble epoxide hydrolase (sEH), and SIRT2 by DEL and ML combination. Such reports mainly aim at classical targets such as kinases and receptors with explicit ligand binding sites and many active scaffolds. Hence, it is easier to conduct DEL library building to obtain many functional building blocks. Then, based on many positive ligands/samples, the problem of uneven sample distribution is avoided, facilitating ML modeling greatly. However, the main disadvantage is that having a novel skeleton will be much more challenging. Hence, it probably will meet the dilemma of being a fast-follower as previous DDR1 inhibitors found by AIDD. In this case, the most critical role of DEL as a promising tool to find potential first-in-class hits was not fully realized (Walters and Murcko, 2020).

For drug development, ML is a well-established, proven tool that can dramatically improve the success and efficiency of drug optimization. Therefore, DEL-ML combined application should not be absent from finding ligands for PPI targets, especially for those with adequate antibody candidates but no small molecule inhibitors. This combination is expected to discover first-in-class hit compounds through DEL, and then the screening data can be efficiently analyzed and iterated through ML to obtain highly bioactive compounds. However, in such case, ML modeling may face a stubborn difficulty-uneven sample distribution caused by too few positive samples/hits. Uneven distribution of samples creates different obstacles for different ML models. More data tends to outperform better algorithmic models. In 2017, Altae-Tran et al. used the One-Shot Learning to generate molecular graphs to build a model with a minimal number of samples on drug property prediction. However, whether this method is suitable for analyzing the DEL’s highly uneven data distribution remains unknown (Altae-Tran et al., 2017; Lu et al., 2020; Wang et al., 2020).

Immune checkpoint inhibitors (ICI), a type of tumor immunotherapy, have attracted much attention for their remarkable anti-tumor activity in pre-clinical and clinical studies. ICI representative drugs like PD-1 inhibitors Keytruda and Nivolumab have reached 30 billion dollars in terms of global sales amount (Clarke et al., 2018). T cell immunoglobulin and immunoreceptor tyrosine inhibitory motif (T cell immunoglobulin and ITIM domain, TIGIT), another type of immune checkpoint (IC), was discovered by Yu et al. through bioinformatics in 2009. The expression of malignant tumor-infiltrating lymphocytes is significantly increased, making TIGIT a potential blockbuster target for cancer immunotherapy (Yu et al., 2009). In numerous pre-clinical and clinical trials, anti-TIGIT antibody therapy has achieved significant tumor-suppressive efficacy (Joller et al., 2011; Zhang et al., 2018; Preillon et al., 2021). Currently, most TIGIT inhibitors in drug development and clinical stages are antibodies, while no peer-reviewed literature has reported small molecule TIGIT inhibitors (Rotte et al., 2021). Biological drug development faces many safety challenges, mainly immunogenicity, including anti-TIGIT antibodies. After biological drugs enter the human body, a cytokine storm could occur, causing a strong immune response. This known pathway resulted in various severe clinical side effects. Compared with biological drugs, small molecule drugs have much less risk of immunogenicity, with significant advantages like low cost in R&D and manufactory and diversified administration approaches (Prueksaritanont and Tang, 2012; Wan, 2016; Makurvet, 2021). Therefore, it is still necessary to develop small-molecule inhibitors for TIGIT target.

**SCHEME 1:**
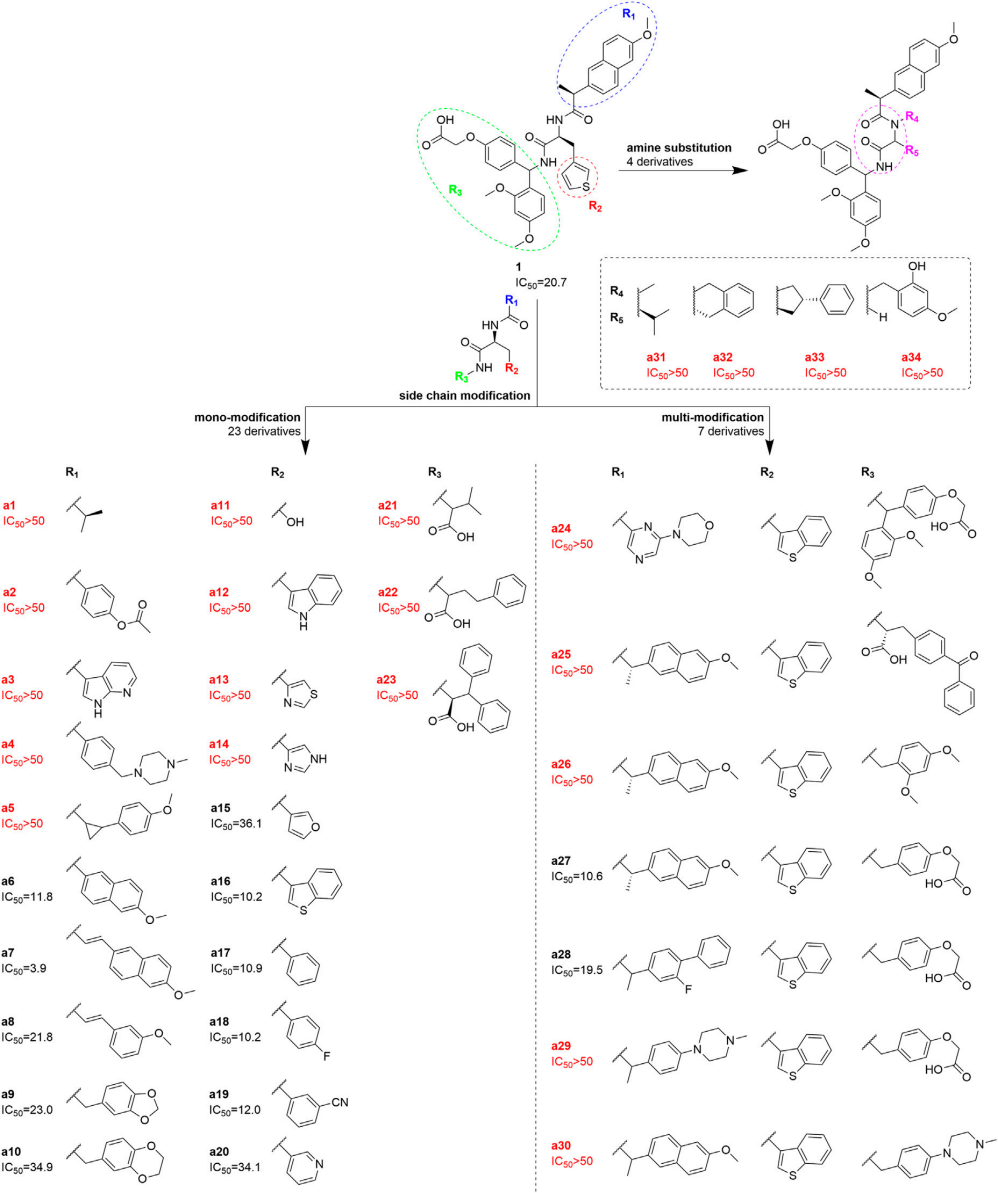
The structure and corresponding protein-protein blocking activity for TIGIT/CD155 complex (IC_50_/µM) of compound **1** and its derivatives **a1**-**a34**. Derivatives **a1**-**a23** were single-site substituted (R_1_, R_2,_ and R_3,_ respectively); Derivatives **a24**-**a30** were multi-site substituted (R_1_ -R_3_); **a31**-**a34** were derivatives with modifications including cyclization on the scaffold amine group (R_4_) and ortho carbons group (R_5_).

In this study, the own-built DEL platform was used to construct a 30-million-member DNA-encoded library composed of 3 building blocks, followed by affinity binding screening on the TIGIT target. The hit compound was identified with high post-selection counts and enriched folds (EF). Indeed, after off-DNA synthesis, a moderately active small molecule hit compound **1** was found (half-fold binding inhibition for TIGIT/CD155 complex, IC_50_ = 20.7 µM). A series of derivatives **a1**-**a34** were obtained by structural modification, including the more active molecule **a7** (IC_50_ = 3.9 µM, Scheme 1). Furthermore, to comprehensively analyze the DEL’s dataset, we input it for ML modeling, exploring various positive sample amplification methods to address the problem of highly uneven sample distribution (only one positive hit **1,** and the count value distribution is highly uneven). This model has a hit rate of around 75% for the high-score derivative samples in the validation and test sets. With such a well-established model, it is expected to be a good drug-hunter for TIGIT inhibitors when screening virtual molecule databases like ChEMBL and ZINC in the future.

## 2 Materials and experiments

### 2.1 DNA-encoded library screening, chemical synthesis, and bio experiments

DEL screening, chemical synthesis, bio-activity experiments, and characterization of compounds are described in supporting information.

### 2.2 Machine learning modeling

#### 2.2.1 Data preparation

The compounds from DEL screening and structure modification (divided into type 1 and 2) are used as the model’s training, validation, and test sets. Among them, the hit compound **1** in DEL is a trisynthon molecule composed of three building blocks. The corresponding machine learning model is established by transforming molecule structure as molecular fingerprints.

#### 2.2.2 Calculation of score value

The bioactivity of each trisynthon can calculate from the corresponding count and enrichment fold (EF) under different experimental conditions, including the presence of the target-library, beads-library, and target-DNA-tag, respectively. To eliminate the dimensional differences in these conditions, data obtained were normalized firstly, and the score was calculated based on normalized count and EF values. The score calculation formula is described as follows:

$$\mathrm{count}=\mathrm{coun}t_{\mathrm{norm}\_\mathrm{target}}-\mathrm{coun}t_{\mathrm{norm}\_\mathrm{beads}}-\mathrm{coun}t_{\mathrm{norm}\_\mathrm{tag}}$$ (1)

$$\mathrm{EF}=EF_{\mathrm{norm}\_\mathrm{target}}-EF_{\mathrm{norm}\_\mathrm{beads}}-EF_{\mathrm{norm}\_\mathrm{tag}}$$ (2)

score=a∗count+b∗EF (3)

Among them, count_norm_target_, count_norm_beads_, and count_norm_tag_ represent the normalized value of count value under the above three different conditions, which aim to eliminate the undesired environmental effects and interaction effects of beads and DNA-tag with the target, respectively. The same rule was applied to EF normalized values. According to the principle of protein-ligand affinity and PCR amplification, high count and EF values mean the molecule has high-affinity activity. Based on our previous experience, the count value significantly impacts the affinity activity. Thus, when defining the weight coefficient of the score for formula score = a*count + b*EF, the count value is given a higher weight as *a* = 0.8 with EF value as *b* = 0.2. According to this calculation formula, the unique positive sample/hit **1** (IC_50_ = 20.7 µM) in the DEL library scored 0.85 in the preliminarily established model. Since the count and EF values cannot be obtained reversely for the structure-modified derivatives, we also need to assign a score value to them. According to the indicated relationship between the IC_50_ value and the count value, the following rule was set: IC_50_ value (µM) < 10, 10 to 20, 20 to 30, 30 to 40, 40 to 50, and > 50 is given score 1, 0.9, 0.8, 0.7, 0.6, and 0 corresponding.

#### 2.2.3 Dataset partitioning

Through DEL screening, a total of 1,104,808 valid data were generated and available for model building. Undersampling was firstly employed to pre-process the DEL dataset because there were too few positive samples. We sort the dataset according to the score value from low to high and sample with interval N. At the same time, since there is only one positive sample, to ensure the model can learn sufficient information from the positive sample, the sampling multiple is set for oversampling the top 100 samples with highest score value. The digital bit value on the molecular fingerprint is randomly modified to generate more positive samples with high similarity. The generated sample score value is based on the original sample plus random (−0.1, 0.1) interval treatment. 1) The data obtained by the combination of undersampling and oversampling is used as the training set; 2) Excluding the training set in the DEL dataset, 100 thousand samples are randomly selected as the validation set 1, and 100 thousand samples are randomly selected from the remaining DEL dataset as Test set 1; 3) The 34 molecules obtained by chemical modification are arranged in order of activity from high to low, and the odd number is defined as the validation set 2 with the even one is the test set 2.

#### 2.2.4 Molecular representation

Simplified molecular input line entry specification (SMILES) sequence can represent each building block in DEL. RDKit provided a smarts-based reaction according to the offered SMILES sequence and smarts template (Tosco et al., 2014). Input the SMILES of each building block accompanied with smarts reaction template resulted in the SMILES sequence of trisynthon product. Representing molecules into the dataset required for training models is an important step. Different molecule representation methods can be applied to various model architectures for training models. Commonly used molecular representations include: 1) molecular fingerprints, which encode molecular structure with a series of binary numbers that indicate the existence of specific substructures; 2) quantum physical chemistry and differential topology-based representation, which statisticians and cheminformatics usually apply 3) SMILES strings, which uniquely describe the structure of molecules by representing them as line symbols; 4) molecular graph, representing molecular pictures as line symbols; Graphing structural data-the atoms of the drug are used as graph nodes with the chemical bonds connecting the atoms are used as the graph edges (Sun et al., 2020). This study presents molecules by an extended connectivity fingerprint (ECFP) system with dimensions of 1024 or 2048 and a radius of 2 or 3 (Rogers and Hahn, 2010).

#### 2.2.5 Loss function and evaluation metrics

Since the classification model cannot correctly distinguish the high and low score values, we established a regression model for training. The model was learned by optimizing the training set MSE. At the same time, the sum of the MSE from the validation sets 1 and 2, together with the percentage of positive samples (valid2_ratio) in the validation set 2, are used to adjust the model. The model is adjusted according to the highest valid_ratio, and the corresponding sum of valid1_mse and valid2_mse is less than 0.15. Finally, the model is evaluated by analyzing the sum of MSE of test sets 1 and 2 and the percentage of active compounds with higher scores in test set 2 (test2_ratio).

#### 2.2.6 Machine learning modeling

The undersampling interval N in the training dataset, and generation coefficient of the positive sample, radius, and dimensions of molecular fingerprints, the number of hidden layers of the Multilayer perceptron (MLP) (Pinkus, 1999), the number of hidden units in the light gradient boosting machine (lightGBM) (Ke et al., 2017) are defined as hyperparameters. The grid search method is used. In the lightGBM model, the optimal parameters are as follows: the molecular fingerprint radius = 3, nBits = 2048, bagging_fraction = 0.8, feature_fraction = 0.76, lambda_l1 = 10, lambda_l2 = 10, and the learning rate = 0.5, N = 8, oversample_multiple = 800. In such case, the number of samples in the training set used is 146,852 for undersampling, and 80,000 for oversampling; for the MLP architecture, the optimal parameters are the input dimension = 1024, hidden layers = 1, optimizer is Adam, learning rate = 0.005, the hidden units = 256, the activation functions are “relu” and dropout = 0.8. In addition, the output unit = 1 and the activation function of the output layer is “softplus.” The corresponding fingerprints is nBits = 1024, radius = 2, N = 6, oversample_multiple = 400. In such case, the number of samples in the training set used is 192,468 for undersampling, and 40,000 for oversampling.

#### 2.2.7 Structure-activity relationship visualization

The atom-centered Gaussian visualization principle is defined as follows. Calculate the score of the original fingerprint, followed by masking defined bits in the molecular fingerprint. After that, the masked score of the bits in the molecular fingerprint was calculated. The weight score corresponding to the bits was defined as the difference between these two values. Normalize the bit weight by dividing it by the highest-scoring weight value. The normalized weight values were used to calculate the Gaussian distribution centered on the atom, generating a molecular map. Different colors indicate the contribution of each substructure to the prediction score.

## 3 Results and discussion

### 3.1 DNA-encoded library screening experiments

To identify potential binders for TIGIT, we constructed a tripeptide DEL containing 30 million unique compounds by a split-and-pool strategy (Supplementary Figure S1A). After DEL qualification, we performed screening using standard immobilized target protein selection methods (Figure 1) (Decurtins et al., 2016). Briefly, purified TIGIT was immobilized on NHS beads and incubated with a 30 million-member DELs, followed by repeated washing to remove non-adhesives. The binders were then recovered, PCR amplified, and the selected library DNA were sequenced by NGS. Parallel DEL screening was performed on beads without protein immobilization to exclude nonspecific binding between the library and blank beads.

**FIGURE 2:**
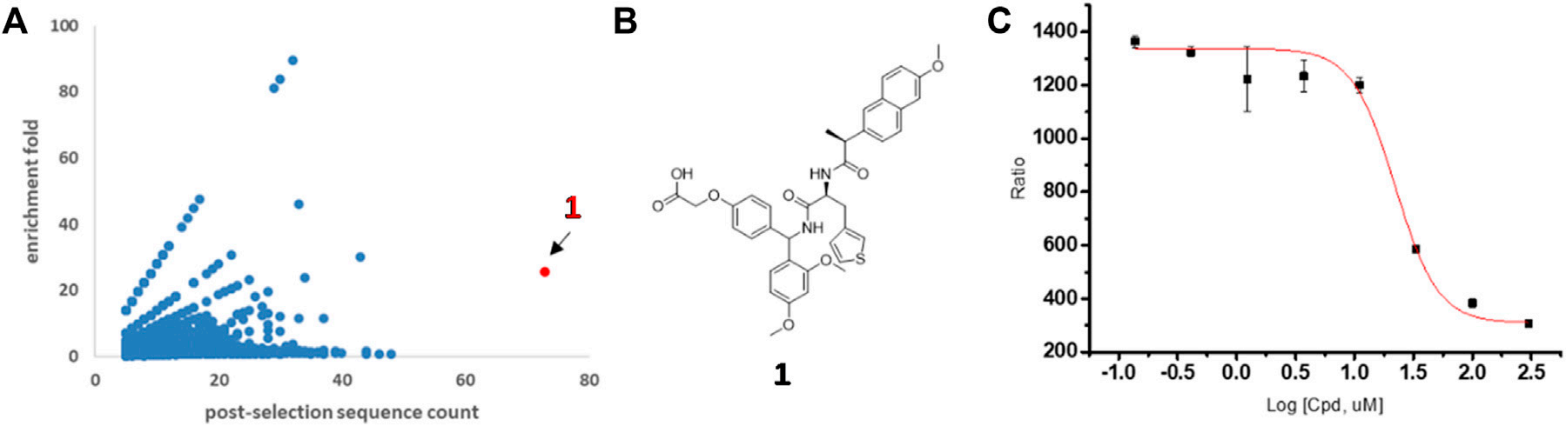
Discovery of novel TIGIT ligands by DEL screening. **(A)** Two-dimensional display of post-selection DNA sequencing data from TIGIT selection. *x*-axis: post-selection sequence counts; *y*-axis: post-selection enrichment fold = (post-selection counts %)/(pre-selection counts %). **(B)** Structure of hit **1**. **(C)** Blocking effects on TIGIT/CD155 complex in HTRF assay for hit **1**.

The resulting NGS data were processed with computational software to calculate individual codon sequences and displayed in a two-dimensional format (Figure 2A). Compounds possessing significant binding affinity against TIGIT resulted in high post-selection counts and enrichment folds and were located in the upper right corner of the scatterplot. Thus, the most enriched library molecule was found in the TIGIT screening and marked as potential ligand **1** (highlighted red in Figure 2A), whereas it was not observed in the bead-only control (Supplementary Figure S1B). The hit compound **1** was re-synthesized by “off-DNA” (the structure is shown in Figure 2B), and corresponding binding affinity was tested utilizing homogeneous time-resolved fluorescence (HTRF) technology. Compound **1** performed a moderate binding affinity from the inhibition assay with an IC_50_ value of 20.7 µM (Figure 2C).

To further chemically optimize compound **1** for better binding affinity, its side chains R_1_, R_2_, and R_3_ were modified before performing a similar HTRF assay (Scheme 1). Among all derivatives, **a6**, **a7**, **a16**, **a17**, **a18**, **a19**, and **a27** had improved TIGIT binding effects, while the others were not significantly improved or even lost blocking activities. Among them, compound **a7** had the highest binding affinity. Their structures and IC_50_ values were further involved in machine learning model construction.

### 3.2 Machine learning modeling

#### 3.2.1 Undersampling and oversampling

After sorting the training set from high to low activity, the training set was adjusted by controlling the undersampling interval N and the multiple of repeated sampling. Parameter selection is performed with the highest valid_ratio value and the smallest sum of the corresponding valid1_mse and valid2_mse. Such adjustments will produce results that achieve best on the validation but not on the test set. In the MLP model, when the oversampling multiple is 400 and 600 and N = 6, a maximum valid_ratio2 value and a relatively small value for the sum of valid1_mse and valid2_mse were obtained. At this point, the model performed best on the validation set. However, in the test set, the best performance is when the oversampling multiples are 400 and 600, and N is 8 and 4, respectively (Table 1). A similar result was observed in lightGBM model (Table 2). Therefore, a better prediction method may be setting a threshold for the above two sets firstly and predicting the score that meets the threshold, followed by taking the average value.

**TABLE 1:** The performance of MLP with representative undersampling interval N and repeated sampling multiples (complete data were provided in supporting information).

Model	Oversample_multiple	N	Train_mse	Valid1_mse	Valid2_mse	Valid2_ratio	Test1_mse	Test2_mse	Test2_ratio
MLP	400	4	0.0038	0.0039	0.153	0.33	0.0040	0.104	0.17
MLP	400	6	0.0037	0.0041	0.137	0.67	0.0041	0.101	0.50
MLP	400	8	0.0038	0.0045	0.150	0.50	0.0045	0.111	0.67
MLP	600	2	0.0038	0.0038	0.147	0.17	0.0038	0.100	0.33
MLP	600	4	0.0037	0.004	0.148	0.50	0.0041	0.104	0.67
MLP	600	6	0.0037	0.0043	0.142	0.67	0.0043	0.098	0.50

**TABLE 2:** The performance of lightGBM with representative undersampling interval N and repeated sampling multiples (complete data were provided in supporting information).

Model	Oversample_multiple	N	Train_mse	Valid1_mse	Valid2_mse	Valid2_ratio	Test1_mse	Test2_mse	Test2_ratio
lightGBM	600	4	0.0074	0.0036	0.172	0.50	0.0046	0.162	0.67
lightGBM	600	6	0.0027	0.0030	0.167	0.50	0.0029	0.191	0.33
lightGBM	600	8	0.0036	0.0045	0.170	0.33	0.0034	0.134	0.67
lightGBM	800	2	0.0064	0.0039	0.178	0.50	0.0045	0.170	0.50
lightGBM	800	4	0.0036	0.0032	0.131	0.67	0.0043	0.168	0.33
lightGBM	800	6	0.0086	0.0054	0.172	0.50	0.0054	0.172	0.33
lightGBM	800	8	0.0032	0.0045	0.138	0.67	0.0033	0.138	0.50

#### 3.2.2 Positive sample generation

To compare the difference between positive sample generation and direct oversampling, the bits on the molecular fingerprints are randomly changed, including 1) randomly replacing the value of bit 0 on 1-4 molecular fingerprints with 1; 2) randomly reversed bit values on 1-4 molecular fingerprints. The generative model did not outperform the oversampling one when the number of molecules generated was the same as the oversampling. Additionally, we found a significant drop in valid2_ratio and test2_ratio in model performance when such noise was introduced in MLP, while it was not observed in lightGBM. The difference may be that MLP is more sensitive to such noise than lightGBM. (Table 3).

**TABLE 3:** The average result obtained by randomly setting the value of bit 0 on 1–4 molecular fingerprints to 1 and repeating ten times.

Model	Modify_bit^a^	Modify_type^a^	Train_mse	Valid1_mse	Valid2_mse	Valid2_ratio	Test1_mse	Test2_mse	Test2_ratio
MLP	1	1	0.0049	0.0043	0.157	0.32	0.0041	0.143	0.37
MLP	1	2	0.0052	0.0045	0.158	0.36	0.0043	0.152	0.33
MLP	2	1	0.0051	0.0043	0.164	0.41	0.0041	0.160	0.40
MLP	2	2	0.0052	0.0043	0.161	0.40	0.0041	0.161	0.35
MLP	3	1	0.0051	0.0042	0.166	0.28	0.0040	0.163	0.35
MLP	3	2	0.0055	0.0045	0.163	0.38	0.0043	0.167	0.38
MLP	4	1	0.0052	0.0041	0.171	0.28	0.0040	0.165	0.45
MLP	4	2	0.0056	0.0042	0.166	0.37	0.0040	0.171	0.28
lightGBM	1	1	0.0047	0.0046	0.140	0.50	0.0043	0.128	0.62
lightGBM	1	2	0.0058	0.0049	0.189	0.48	0.0044	0.237	0.39
lightGBM	2	1	0.0045	0.0046	0.138	0.46	0.0042	0.128	0.62
lightGBM	2	2	0.0067	0.0054	0.176	0.48	0.0050	0.227	0.35
lightGBM	3	1	0.0042	0.0044	0.137	0.50	0.0041	0.127	0.67
lightGBM	3	2	0.0055	0.0049	0.163	0.55	0.0045	0.222	0.35
lightGBM	4	1	0.0042	0.0044	0.136	0.50	0.0041	0.128	0.67
lightGBM	4	2	0.0047	0.0045	0.163	0.52	0.0042	0.216	0.37

a Modify_bit is to modify the number of digits of the fingerprint randomly, modify_type = 1 is the performance when the bit of the fingerprint is set to 0 and 1, and modify_type = 2 is the reverse performance of setting.

#### 3.2.3 Model performance

**FIGURE 3:**
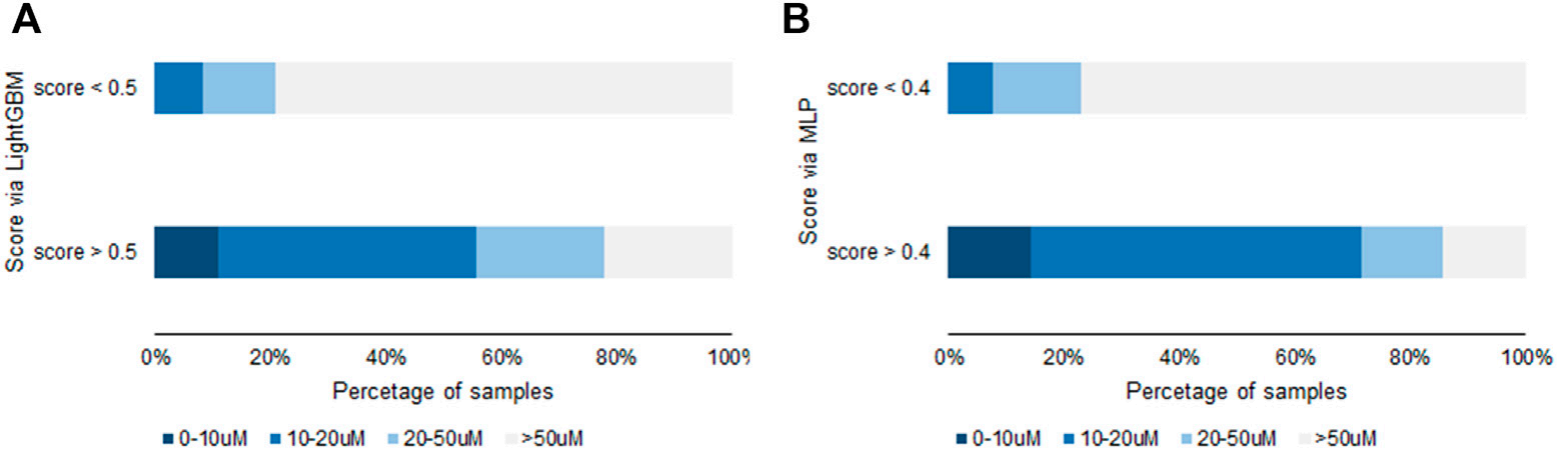
The relationship between lightGBM **(A)** and MLP **(B)** prediction score and IC_50_ value.

MLP and lightGBM models were built and compared. Initially, the unique positive sample **1** was applied for training with an oversample. The resulted model’s unsatisfying performance is shown in Tables 4, 5. This imperfect model may result from too simple positive sample structure. The features of positive samples are not learned sufficiently. Afterward, the model’s performance changed by adding a positive sample from the validation set to the training set, with the identical oversample multiples for both models. In the case of one more positive sample, the model performed much better with **a6** than the other positive samples (Scheme 1). In the validation set 2 and test set 2 from the lightGBM model, there are nine samples with a score greater than 0.5, 7 of which are active compounds (IC_50_ < 50 µM), with an overall hit rate of 78%. In the set with a score less than 0.5, the hit rate is less than 30%. This rule is observed in both validation and test sets. Similar results were observed for the MLP model. The overall hit percentage of these two models is shown in Figure 3. In addition, we also tried two additional positive samples including **a6**. Unfortunately, the model’s performance did not improve, meaning that for samples with relatively high similarity, adding a minimal number of samples may not be beneficial. Introducing more positive samples is still the key to improving the model’s generalization performance.

**TABLE 4:** Model performance without additional positive sample.

Model	Train_mse	Valid1_mse	Valid2_mse	Test1_mse	Test2_mse	Valid2_ratio	Test2_ratio
MLP	0.0026	0.0039	0.163	0.0039	0.189	0.33	0.33
lightGBM	0.0021	0.0035	0.230	0.0035	0.225	0.33	0.33

**TABLE 5:** Model performance with additional positive sample **a6**.

Model	Train_mse	Valid1_mse	Valid2_mse	Test1_mse	Test2_mse	Valid2_ratio	Test2_ratio
MLP	0.0037	0.0041	0.137	0.0040	0.102	0.67	0.5
lightGBM	0.0087	0.0067	0.132	0.0067	0.145	0.67	0.5

#### 3.2.4 Performance of molecular fingerprints

When using different molecular fingerprint settings, the results of the positive samples from models will also be different. When using molecular fingerprints with nBits = 2048 and radius = 3, the samples with score > 0.5 from lightGBM included **a7**, **a15**-**a20**. On the other hand, when using molecular fingerprints with setting nBits = 1024, radius = 2, the samples with scores > 0.5 by lightGBM and >0.4 by MLP included **a7**, **a15**-**a19,** and **a27**. Therefore, simultaneously selecting different molecular fingerprints for modeling is conducive to obtaining a more comprehensive screening for the model.

#### 3.2.5 Structure-activity relationship-specific analysis

**FIGURE 4:**
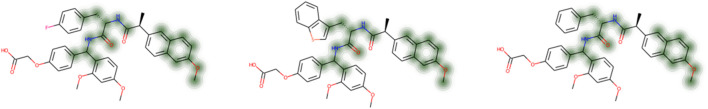
Gaussian distribution of compounds with high predicted scores.

Visualizing the important features learned by the model is helpful for medicinal chemists to understand the model better and obtain the structure-activity relationship (SAR). Figure 4 shows a plot of the Gaussian distribution with high model prediction scores with some molecules as examples, where green indicates fragments that are conducive to a higher score. Almost all compounds with high predicted values contain the same fragments, including the aromatic 2-methoxy-6-naphthalene and the amino acid scaffold (S)-1-azaneyl-2-(oxo-methyl -amino)-3-propan-1-one. These fragments are considered beneficial for the enrichment of compounds and can improve affinity activity. We also noticed that the highly active compounds have other common fragments, such as the carboxyl functional group in R_3_. Still, this functional group also frequently appears in other inactive compounds, so the ML model comprehensively learned that its positive contribution is not confirmed. Consequently, the aromatic naphthalene of R_1_ in the parent compound is an active functional group and should not be modified. While R_2_ and R_3_ have the potential for chemical modification, the specific SAR remains to be explored.

## 4 Conclusion

Either DEL or AI applications in drug discovery emerged during the past decade. Their combination for discovering and developing new PPI inhibitors is also promising to provide vital drug candidates. With more academic institutes and the pharmaceutical industry investing in DEL technology development, taking full advantage of the DEL-generated terabyte-level dataset, including negative data, is a coming-up task. Specifically, efficiently constructing a model will be much more challenging with a few positive or even only one positive sample, which is nearly unavoidable in the real world. This study analyzed the big data with one unique positive sample hit **1** generated by DEL screening on the TIGIT target. A series of derivatives **a1**-**a34** beyond the DEL dataset, including higher active derivative **a7**, were chemically synthesized to validate and test the ML models. Moreover, the difference between fingerprint molecule generation and oversampling or undersampling methods was investigated to reach an even distributed dataset for MLP and lightGBM models. The systemic investigation of building ML models based on a tiny number of positive samples provides help for the establishment of subsequent models. To our knowledge, this is the first reported small molecule inhibitors against TIGIT in the peer-reviewed literature. This study will facilitate developing small molecule inhibitors against PPI targets for tumor immunotherapy. The further bioactivity investigation of the hit and derivatives and application of ML models for virtual database screening is still ongoing.

## Data Availability

The original contributions presented in the study are included in the article/Supplementary Material, further inquiries can be directed to the corresponding authors.
