# Supplementary material for: Discovery of TIGIT inhibitors based on DEL and machine learning
Source: Front Chem. 2022 Jul 26;10:982539. doi: 10.3389/fchem.2022.982539 (PMC9360614; doi:10.3389/fchem.2022.982539)
Supplement: Supplementary file 1 [file DataSheet1.docx]

Supplementary Material

## DNA-encoded library screening against TIGIT

15 μL NHS beads NHS-activated Sepharose^TM^ 4 Fast Flow was washed twice with immobilization buffer (0.2 M NaHCO_3_, 0.5 M NaCl, pH 8.3) before immobilization. 200 pmol TIGIT was diluted to 100 μL with immobilization buffer, followed by incubation with NHS beads at 4 ℃ for 16 hrs. After incubation, excessive NHS groups were quenched by 0.1 M Tris-HCl, pH 8.5 at 4 ℃ for 4 hrs. The beads were then washed with 0.1 M Tris-HCl, pH 8.5 for 3 times and 0.1 M NaOAc, 0.5 M NaCl, pH 4.5 for 3 times. The washing step was repeated for another 5 times to ensure the removal of un-immobilized proteins.

500 pmol library was diluted into 100 μL total volume with PBS supplemented with 0.01 mg/mL Herring Sperm DNA before selection. The TIGIT immobilized beads were rinsed twice with PBS and incubated with library at 4 ℃ for 4 hrs. After incubation, beads were washed with PBS for 10 times to remove unbound molecules, followed by elution of remained library with 20 μL ddH_2_O at 95℃ for 20 min. The elution was subjected to PCR amplification directly and the resulting products were submitted for Illumina sequencing.


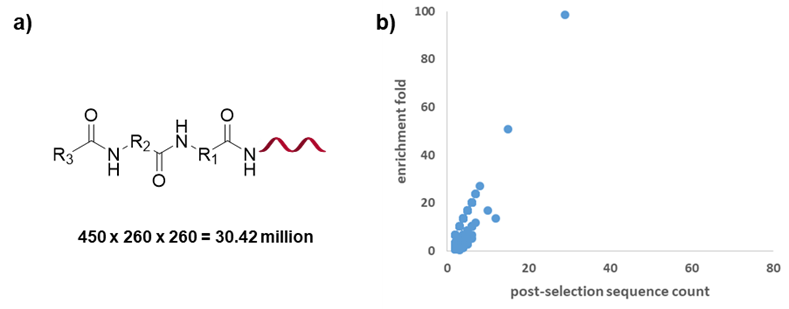


Figure **S1.** a) The structure of 30-million compound. b) Two-dimensional display of post-selection DNA sequencing data from beads-only selection. x-axis: post-selection sequence count; y-axis: post-selection enrichment fold = (post-selection counts%/)/(pre-selection counts%).

## HTRF assay

For all compounds, the binding affinities were measured with TIGIT/CD155 Binding Assay (Immuno-Oncology, Part # 63ADK000CB07PEG) following the standard kit protocol.

1. **Synthesis and characterization of new compounds**

**3.1** General procedure for preparation of compounds

*Compound* ***1*** *was synthesized using standard Fmoc chemistry:*

1. Resin preparation: To the 2-CTC Resin (0.3 mmol, 1.00 eq, Sub 1.00 mmol/g) in DMF was agitated with N_2_ for 2h at 20^o^C. Then the mixture was filtered to get the resin.
2. 20% piperidine in DMF (10 ml) was added and agitated the resin with N_2_ for another 30 min. The resin was washed with DMF (10 ml * 5) and filtered to get the resin.
3. Coupling: A solution of HATU (0.21 g, 0.57 mmol, 1.9 eq) and Fmoc-Cys(Me)-OH (0.22 g, 0.60 mmol, 2.00 eq) in DMF (5 mL) was added DIEA (0.21 ml, 1.20 mmol , 4.00 eq) to the resin and agitated with N_2_ for 30 min at 20^o^C. The resin was then washed with DMF (10 ml * 4).
4. Deprotection: 20% piperidine in DMF (10 mL) was added to the resin and the mixture was agitated with N_2_ for 30 min at 20^o^C. The resin was then washed with DMF (10 ml * 5).

Repeat above step 3 to 4 for the coupling of following amino acids: (1-3).

Note:

| **#** | **Materials** | **Coupling reagents** |
| --- | --- | --- |
| 1 | Fmoc-Cys(Me)-OH (2.00 eq) | HATU (1.9 eq) and DIEA (4.00 eq) |
| 2 | Fmoc-L-Homephenylalanine (2.00 eq) | HATU (1.9 eq) and DIEA (4.00 eq) |
| 3 | 2-Nitrocinnamic acid (2.00 eq) | HATU (1.9 eq) and DIEA (4.00 eq) |

*Peptide Cleavage and Purification:*

1. Add cleavage buffer (20%HFI/80%DCM；JY-24 used the buffer of 30%TFE/70%DCM) to the flask containing the side chain protected peptide at room temperature and stir 15 min for 2 times.
2. The peptide was precipitated with cold isopropyl ethyl.
3. Filter and collect the filter cake.
4. Isopropyl ethyl washes two more times.
5. Dry the crude peptide under vacuum overnight.
6. The crude peptide was purified by prep-HPLC (A: 0.05% HCl in H_2_O, B: ACN) to give the final product **1** (0.20 mmol, NH_4_HCO_3_) as an white solid.

*Purification conditions:*

| Separation condition | |
| --- | --- |
| Dissolution condition | Dissolve in 40%DMF-H_2_O |
| Instrument | Gilson GX-281 |
| Mobile Phase | A: H_2_O (0.01mmol/L NH_4_HCO_3_ in H_2_O) |
|  | B: CH_3_CN |
| Gradient | 10-70-50 min. Retention time:27.5 min |
| Column | Gemini,5um,c18,110A+luna,c18,10um,100A |
| Flow Rate | 20 mL/Min |
| Wavelength | 214/254 nm |
| Oven Tem. | 30^o^C |

*The other compounds were obtained by similarly method.*

**3.2 Characterization of compounds**

Compound **1**

^1^H NMR (400 MHz, DMSO-d_6_) δ 8.33 (d, *J* = 8.7 Hz, 2H), 7.77 – 7.65 (m, 2H), 7.41 (dd, *J* = 12.7, 10.0 Hz, 2H), 7.14 (ddd, *J* = 53.3, 46.3, 10.9 Hz, 3H), 6.82 (dd, *J* = 18.8, 7.8 Hz, 2H), 6.64 (dd, *J* = 30.1, 8.6 Hz, 1H), 6.38 (dd, *J* = 46.6, 8.5 Hz, 1H), 6.27 – 5.99 (m, 1H), 4.65 (s, 1H), 4.38 (d, *J* = 15.6 Hz, 1H), 3.84 (t, *J* = 11.8 Hz, 3H), 3.70 (s, 1H), 3.69 – 3.54 (m, 3H), 3.09 – 2.78 (m, 1H), 1.27 (dd, *J* = 16.8, 10.3 Hz, 3H). MS(ESI): [M+H]^+^ m/z calcd. For C_38_H_39_N_2_O_8_S = 683.2, found = 683.5.

Compound **a1**

^1^H NMR (400 MHz, DMSO-d_6_) δ 8.50 (dd, *J* = 23.3, 8.7 Hz, 1H), 7.93 (dd, *J* = 12.2, 8.6 Hz, 1H), 7.40 (ddd, *J* = 6.2, 5.0, 2.9 Hz, 1H), 7.24 – 6.86 (m, 5H), 6.79 (dd, *J* = 12.1, 8.8 Hz, 2H), 6.63 – 6.41 (m, 2H), 6.19 (dd, *J* = 11.2, 8.6 Hz, 1H), 4.59 (d, *J* = 3.5 Hz, 3H), 3.81 – 3.65 (m, 6H), 3.04 – 2.69 (m, 3H), 0.89 (ddd, *J* = 9.4, 8.9, 4.7 Hz, 6H). MS(ESI): [M-H]^+^ m/z calcd. For C_28_H_31_N_2_O_7_S = 539.2, found = 539.2.

Compound **a2**

^1^H NMR (400 MHz, DMSO-d_6_) δ 8.63 (dt, *J* = 19.4, 8.6 Hz, 2H), 7.97 – 7.79 (m, 2H), 7.41 (dt, *J* = 7.6, 3.9 Hz, 1H), 7.17 (dddd, *J* = 23.6, 8.9, 6.0, 4.0 Hz, 6H), 6.98 (d, *J* = 8.6 Hz, 1H), 6.80 (dd, *J* = 8.5, 7.4 Hz, 2H), 6.64 – 6.43 (m, 2H), 6.35 – 6.15 (m, 1H), 4.89 – 4.72 (m, 1H), 4.62 (d, *J* = 1.5 Hz, 2H), 3.71 (dd, *J* = 26.2, 2.5 Hz, 6H), 3.14 – 2.99 (m, 2H), 2.28 (d, *J* = 2.3 Hz, 3H). MS(ESI): [M-H]^+^ m/z calcd. For C_33_H_31_N_2_O_9_S = 631.2, found = 630.9.

Compound **a3**

^1^H NMR (400 MHz, DMSO-d_6_) δ 12.14 (s, 1H), 8.68 (dd, *J* = 26.0, 8.7 Hz, 1H), 8.39 (d, *J* = 7.9 Hz, 1H), 8.27 (dt, *J* = 6.4, 2.6 Hz, 2H), 8.18 (t, *J* = 8.7 Hz, 1H), 7.52 – 7.38 (m, 1H), 7.30 – 7.05 (m, 5H), 6.97 (d, *J* = 8.7 Hz, 1H), 6.79 (dd, *J* = 8.8, 2.7 Hz, 2H), 6.62 – 6.44 (m, 2H), 6.23 (t, *J* = 8.8 Hz, 1H), 4.98 (tdd, *J* = 15.3, 11.7, 5.8 Hz, 2H), 4.61 (d, *J* = 3.0 Hz, 3H), 3.73 (d, *J* = 7.7 Hz, 3H), 3.66 (d, *J* = 9.4 Hz, 3H), 3.17 – 2.96 (m, 2H). MS(ESI): [M+H]^+^ m/z calcd. For C_32_H_31_N_4_O_7_S = 615.2, found = 615.1.

Compound **a4**

^1^H NMR (400 MHz, DMSO-d_6_) δ 13.71 – 12.27 (m, 1H), 8.69 (dd, *J* = 23.3, 8.5 Hz, 1H), 8.54 (t, *J* = 8.9 Hz, 1H), 7.82 (dd, *J* = 7.9, 5.7 Hz, 2H), 7.50 – 7.36 (m, 3H), 7.16 (ddd, *J* = 51.3, 15.8, 7.6 Hz, 4H), 7.00 – 6.78 (m, 3H), 6.58 – 6.46 (m, 2H), 6.23 (t, *J* = 8.3 Hz, 1H), 4.89 – 4.78 (m, 1H), 4.62 (s, 2H), 3.71 (dd, *J* = 24.8, 3.0 Hz, 9H), 3.38 (s, 2H), 3.17 – 3.02 (m, 4H), 2.78 (s, 3H). MS(ESI): [M+H]^+^ m/z calcd. For C_37_H_43_N_4_O_7_S = 687.3, found =687.2.

Compound **a5**

^1^H NMR (400 MHz, DMSO-d_6_) δ 12.99 (s, 1H), 8.74 – 8.48 (m, 1H), 8.48 – 8.22 (m, 1H), 7.57 – 7.35 (m, 1H), 7.23 – 7.10 (m, 1H), 7.10 – 6.96 (m, 4H), 6.96 – 6.73 (m, 4H), 6.66 – 6.36 (m, 2H), 6.20 (ddd, *J* = 11.9, 8.5, 5.8 Hz, 1H), 4.90 – 4.44 (m, 3H), 3.80 – 3.67 (m, 9H), 3.13 – 2.72 (m, 2H), 2.02 (ddt, *J* = 13.0, 8.8, 7.3 Hz, 2H), 1.32 – 0.97 (m, 2H). MS(ESI): [M+Na]^+^ m/z calcd. For C_35_H_36_N_2_NaO_8_S = 667.2, found = 667.3.

Compound **a6**

^1^H NMR (400 MHz, DMSO-d_6_) δ 8.77 – 8.56 (m, 2H), 8.36 (d, *J* = 4.1 Hz, 1H), 8.01 – 7.78 (m, 3H), 7.53 – 7.36 (m, 2H), 7.36 – 6.89 (m, 6H), 6.85 – 6.67 (m, 2H), 6.61 – 6.43 (m, 2H), 6.29 – 6.19 (m, 1H), 4.87 (s, 1H), 4.45 (s, 2H), 3.90 (d, *J* = 1.8 Hz, 3H), 3.74 (d, *J* = 4.2 Hz, 3H), 3.66 (d, *J* = 6.1 Hz, 3H), 3.17 – 3.05 (m, 2H). MS(ESI): [M+Na]^+^ m/z calcd. For C_36_H_34_N_2_NaO_8_S = 677.2, found = 677.2.

Compound **a7**

^1^H NMR (400 MHz, DMSO-d_6_) δ 12.94 (s, 1H), 8.72 (dd, *J* = 27.1, 8.6 Hz, 1H), 8.35 (dd, *J* = 8.3, 5.0 Hz, 1H), 7.97 (d, *J* = 5.2 Hz, 1H), 7.92 – 7.77 (m, 2H), 7.65 (t, *J* = 7.8 Hz, 1H), 7.59 – 7.39 (m, 2H), 7.34 (s, 1H), 7.25 – 7.15 (m, 2H), 7.12 – 7.00 (m, 2H), 6.98 – 6.88 (m, 1H), 6.80 (ddd, *J* = 15.6, 6.5, 2.0 Hz, 3H), 6.61 – 6.39 (m, 2H), 6.31 – 6.18 (m, 1H), 4.96 – 4.74 (m, 1H), 4.62 (s, 2H), 3.89 (s, 3H), 3.83 – 3.61 (m, 6H), 3.13 – 2.83 (m, 2H). MS(ESI): [M+Na]^+^ m/z calcd. For C_38_H_36_N_2_NaO_8_S = 703.2, found = 703.3.

Compound **a8**

^1^H NMR (400 MHz, DMSO-d_6_) δ 12.97 (s, 1H), 8.72 (dd, *J* = 27.1, 8.6 Hz, 1H), 8.32 (dd, *J* = 8.3, 5.1 Hz, 1H), 7.48 – 7.27 (m, 3H), 7.25 – 6.98 (m, 6H), 6.98 – 6.87 (m, 2H), 6.86 – 6.70 (m, 3H), 6.58 – 6.45 (m, 2H), 6.23 (t, *J* = 9.4 Hz, 1H), 4.89 – 4.75 (m, 1H), 4.61 (d, *J* = 1.1 Hz, 2H), 3.82 – 3.67 (m, 9H), 3.09 – 2.81 (m, 2H). MS(ESI): [M+Na]^+^ m/z calcd. For C_34_H_34_N_2_NaO_8_S = 653.2, found = 653.2.

Compound **a9**

^1^H NMR (400 MHz, DMSO-d_6_) δ 8.39 (ddd, *J* = 140.7, 22.4, 10.4 Hz, 2H), 7.46 – 6.69 (m, 10H), 6.69 – 6.34 (m, 3H), 6.33 – 5.89 (m, 3H), 4.55 (d, *J* = 53.5 Hz, 3H), 3.72 (d, *J* = 15.3 Hz, 6H), 2.86 (dd, *J* = 42.0, 31.5 Hz, 3H). MS(ESI): [M+Na]^+^ m/z calcd. For C_33_H_32_N_2_NaO_9_S = 655.2, found = 655.1.

Compound **a10**

^1^H NMR (400 MHz, CDCl_3_) δ 7.54 – 7.39 (m, 1H), 7.10 (ddd, *J* = 23.0, 4.8, 2.9 Hz, 1H), 7.04 – 6.86 (m, 3H), 6.76 (ddd, *J* = 13.5, 11.4, 6.3 Hz, 5H), 6.65 (dd, *J* = 13.3, 1.8 Hz, 1H), 6.58 – 6.51 (m, 1H), 6.47 – 6.29 (m, 2H), 6.16 (t, *J* = 8.9 Hz, 1H), 4.76 (dt, *J* = 20.4, 7.1 Hz, 3H), 4.22 (d, *J* = 2.9 Hz, 4H), 3.76 (d, *J* = 1.3 Hz, 3H), 3.61 (s, 3H), 3.38 (s, 2H), 3.06 – 2.92 (m, 2H). MS(ESI): [M+Na]^+^ m/z calcd. For C_34_H_34_N_2_NaO_9_S = 669.2, found = 669.2.

Compound **a11**

^1^H NMR (400 MHz, DMSO-d_6_) δ 8.19 (dd, *J* = 38.0, 8.1 Hz, 1H), 7.94 (dd, *J* = 12.6, 8.6 Hz, 1H), 7.73 (ddd, *J* = 16.8, 13.0, 6.7 Hz, 3H), 7.48 (ddd, *J* = 17.5, 8.4, 1.5 Hz, 1H), 7.27 (dd, *J* = 6.7, 2.3 Hz, 1H), 7.13 (ddd, *J* = 9.0, 6.5, 2.5 Hz, 1H), 6.97 – 6.56 (m, 5H), 6.42 (dd, *J* = 6.1, 2.3 Hz, 1H), 6.26 – 6.01 (m, 2H), 4.93 (dt, *J* = 10.9, 5.3 Hz, 1H), 4.57 (d, *J* = 12.3 Hz, 2H), 4.44 – 4.33 (m, 1H), 3.96 – 3.79 (m, 4H), 3.63 (dd, *J* = 26.8, 7.9 Hz, 9H), 1.41 (dd, *J* = 8.3, 7.3 Hz, 3H). MS(ESI): [M+H]^+^ m/z calcd. For C_34_H_37_N_2_O_9_ = 617.2, found = 617.4.

Compound **a12**

^1^H NMR (400 MHz, DMSO-d_6_) δ 10.82 (d, *J* = 10.2 Hz, 1H), 8.43 – 8.14 (m, 2H), 7.66 (ddd, *J* = 30.9, 16.6, 8.5 Hz, 4H), 7.52 – 7.22 (m, 3H), 7.19 – 6.93 (m, 4H), 6.88 – 6.78 (m, 2H), 6.67 (dt, *J* = 17.0, 8.7 Hz, 3H), 6.52 – 6.40 (m, 1H), 6.22 (ddd, *J* = 37.2, 8.5, 2.3 Hz, 1H), 6.10 (d, *J* = 8.4 Hz, 1H), 4.63 (dd, *J* = 55.6, 6.6 Hz, 3H), 3.96 – 3.55 (m, 11H), 3.04 (dtd, *J* = 23.4, 14.4, 8.6 Hz, 3H), 1.26 (d, *J* = 7.0 Hz, 3H). MS(ESI): [M+Na]^+^ m/z calcd. For C_42_H_41_N_3_NaO_8_ = 738.2, found = 738.2.

Compound **a13**

^1^H NMR (400 MHz, DMSO-d_6_) δ 12.98 (s, 1H), 9.01 (d, *J* = 6.6 Hz, 1H), 8.41 – 8.21 (m, 2H), 7.84 – 7.65 (m, 3H), 7.40 (dd, *J* = 16.1, 8.8 Hz, 1H), 7.35 – 7.23 (m, 2H), 7.13 (t, *J* = 7.0 Hz, 1H), 7.01 – 6.39 (m, 6H), 6.17 (tt, *J* = 20.0, 9.8 Hz, 2H), 4.79 (dd, *J* = 14.3, 8.4 Hz, 1H), 4.58 (d, *J* = 18.0 Hz, 2H), 3.85 (s, 3H), 3.82 – 3.70 (m, 2H), 3.69 – 3.41 (m, 5H), 3.20 (dd, *J* = 14.6, 5.8 Hz, 1H), 3.03 (dd, *J* = 14.5, 8.9 Hz, 1H), 1.30 (t, *J* = 6.2 Hz, 3H). MS(ESI): [M+H]^+^ m/z calcd. For C_37_H_38_N_3_O_8_S = 684.3, found = 684.3.

Compound **a14**

^1^H NMR (400 MHz, DMSO-d_6_) δ 13.92 (s, 1H), 8.85 (s, 1H), 8.38 (ddd, *J* = 28.5, 22.3, 8.3 Hz, 2H), 7.82 – 7.60 (m, 3H), 7.50 – 7.22 (m, 3H), 7.21 – 7.08 (m, 1H), 7.06 – 6.70 (m, 4H), 6.61 (d, *J* = 8.6 Hz, 1H), 6.57 – 5.99 (m, 3H), 4.72 (dd, *J* = 15.8, 8.2 Hz, 1H), 4.60 (t, *J* = 15.9 Hz, 2H), 3.95 – 3.77 (m, 4H), 3.67 (dt, *J* = 34.6, 21.2 Hz, 6H), 3.06 (dd, *J* = 14.4, 6.7 Hz, 1H), 2.91 (dd, *J* = 14.7, 7.8 Hz, 1H), 1.35 (dd, *J* = 14.7, 7.5 Hz, 3H). MS(ESI): [M+H]^+^ m/z calcd. For C_37_H_39_N_4_O_8_ = 667.3, found = 667.2.

Compound **a15**

^1^H NMR (400 MHz, DMSO-d_6_) δ 8.32 (ddd, *J* = 19.8, 11.8, 7.5 Hz, 2H), 7.71 (ddd, *J* = 12.2, 11.2, 6.0 Hz, 3H), 7.57 – 7.35 (m, 2H), 7.32 – 7.07 (m, 2H), 6.91 – 6.79 (m, 3H), 6.67 (dd, *J* = 43.3, 8.7 Hz, 2H), 6.54 – 6.02 (m, 5H), 4.76 – 4.50 (m, 3H), 3.73 (ddd, *J* = 50.9, 23.2, 11.4 Hz, 11H), 2.95 (ddd, *J* = 56.2, 15.3, 7.6 Hz, 2H), 1.33 (t, *J* = 7.0 Hz, 3H). MS(ESI): [M-H]^+^ m/z calcd. For C_38_H_37_N_2_O_9_ = 665.3, found = 665.2.

Compound **a16**

^1^H NMR (400 MHz, DMSO-d_6_) δ 8.53 – 8.29 (m, 2H), 7.95 (dt, *J* = 8.3, 7.8 Hz, 2H), 7.69 (ddd, *J* = 12.3, 11.1, 6.2 Hz, 3H), 7.50 – 7.31 (m, 4H), 7.30 – 7.02 (m, 2H), 6.94 – 6.04 (m, 7H), 4.81 (dd, *J* = 14.5, 8.2 Hz, 1H), 4.57 (d, *J* = 10.5 Hz, 2H), 3.93 – 3.53 (m, 10H), 3.14 – 3.00 (m, 1H), 1.26 (d, *J* = 7.0 Hz, 3H). MS(ESI): [M-H]^+^ m/z calcd. For C_42_H_39_N_2_O_8_S = 731.3, found = 731.2.

Compound **a17**

^1^H NMR (400 MHz, DMSO-d_6_) δ 8.43 – 8.12 (m, 2H), 7.86 – 7.33 (m, 4H), 7.32 – 7.01 (m, 7H), 6.94 – 6.59 (m, 5H), 6.51 – 6.03 (m, 3H), 4.75 – 4.48 (m, 3H), 3.73 (ddd, *J* = 49.8, 30.7, 10.7 Hz, 11H), 3.05 – 2.72 (m, 2H), 1.23 (d, *J* = 6.8 Hz, 3H). MS(ESI): [M-H]^+^ m/z calcd. For C_40_H_39_N_2_O_8_ = 675.3, found = 675.3.

Compound **a18**

^1^H NMR (400 MHz, DMSO-d_6_) δ 12.97 (s, 1H), 8.33 (ddd, *J* = 24.7, 16.6, 8.6 Hz, 2H), 7.70 (dt, *J* = 18.3, 8.9 Hz, 3H), 7.38 (dd, *J* = 15.0, 8.5 Hz, 1H), 7.25 (dd, *J* = 13.8, 8.1 Hz, 3H), 7.18 – 7.00 (m, 3H), 6.94 – 6.79 (m, 3H), 6.69 (dd, *J* = 24.5, 8.7 Hz, 2H), 6.44 (d, *J* = 2.1 Hz, 1H), 6.41 – 6.19 (m, 1H), 6.10 (t, *J* = 9.0 Hz, 1H), 4.74 – 4.53 (m, 3H), 3.65 (d, *J* = 7.9 Hz, 3H), 3.58 (s, 2H), 2.97 (dd, *J* = 16.6, 11.2 Hz, 1H), 2.79 (dd, *J* = 14.0, 9.8 Hz, 1H), 1.24 (dd, *J* = 6.7, 4.3 Hz, 3H). MS(ESI): [M+Na]^+^ m/z calcd. For C_40_H_39_FN_2_NaO_8_ = 717.3, found = 717.2.

Compound **a19**

^1^H NMR (400 MHz, DMSO-d_6_) δ 12.95 (s, 1H), 8.58 – 8.27 (m, 2H), 7.89 – 7.52 (m, 6H), 7.52 – 7.30 (m, 2H), 7.26 (dd, *J* = 10.5, 2.4 Hz, 1H), 7.12 (td, *J* = 8.9, 2.5 Hz, 1H), 6.97 – 6.79 (m, 3H), 6.70 (dd, *J* = 27.9, 8.8 Hz, 2H), 6.46 (dd, *J* = 12.9, 2.3 Hz, 1H), 6.31 (ddd, *J* = 51.0, 8.5, 2.4 Hz, 1H), 6.10 (t, *J* = 9.1 Hz, 1H), 4.89 – 4.48 (m, 3H), 3.98 – 3.70 (m, 6H), 3.65 (d, *J* = 5.6 Hz, 3H), 3.28 – 2.96 (m, 2H), 2.85 (dt, *J* = 23.1, 11.5 Hz, 1H), 1.23 (t, *J* = 6.8 Hz, 3H). MS(ESI): [M+Na]^+^ m/z calcd. For C_41_H_39_N_3_NaO_8_ = 724.3, found = 724.2.

Compound **a20**

^1^H NMR (400 MHz, DMSO-d_6_) δ 12.93 (s, 1H), 8.68 (s, 2H), 8.54 – 8.37 (m, 2H), 8.14 (d, *J* = 7.0 Hz, 1H), 7.95 (s, 1H), 7.84 – 7.60 (m, 4H), 7.44 – 7.24 (m, 2H), 7.13 (t, *J* = 8.7 Hz, 1H), 6.99 – 6.82 (m, 3H), 6.71 (dd, *J* = 35.7, 8.6 Hz, 2H), 6.54 – 6.05 (m, 3H), 4.88 – 4.49 (m, 4H), 3.86 (d, *J* = 8.5 Hz, 3H), 3.76 (dd, *J* = 14.8, 8.0 Hz, 2H), 3.65 (s, 3H), 3.58 (s, 2H), 3.16 (dd, *J* = 13.6, 5.8 Hz, 1H), 3.02 – 2.70 (m, 4H), 1.23 (t, *J* = 6.6 Hz, 3H). MS(ESI): [M+H]^+^ m/z calcd. For C_39_H_40_N_3_O_8_ = 677.3, found = 678.2.

Compound **a21**

^1^H NMR (400 MHz, DMSO-d_6_) δ 12.67 (s, 1H), 8.23 (d, *J* = 8.4 Hz, 1H), 7.91 – 7.60 (m, 4H), 7.45 – 7.33 (m, 2H), 7.29 – 6.92 (m, 4H), 4.64 (td, *J* = 9.2, 4.5 Hz, 1H), 4.06 (dd, *J* = 8.4, 5.5 Hz, 1H), 3.91 – 3.71 (m, 4H), 2.95 (ddd, *J* = 23.8, 14.2, 7.0 Hz, 2H), 1.93 (dd, *J* = 12.8, 6.7 Hz, 1H), 1.29 (d, *J* = 7.0 Hz, 3H), 0.71 (dd, *J* = 6.7, 4.5 Hz, 6H). MS(ESI): [M+H]^+^ m/z calcd. For C_26_H_31_N_2_O_5_S = 483.2, found = 483.5.

Compound **a22**

^1^H NMR (400 MHz, DMSO-d_6_) δ 12.62 (s, 1H), 8.24 (d, *J* = 8.1 Hz, 2H), 7.85 – 7.52 (m, 3H), 7.46 – 7.35 (m, 2H), 7.26 – 6.88 (m, 9H), 4.62 (td, *J* = 9.2, 4.6 Hz, 1H), 4.14 – 4.01 (m, 1H), 3.88 – 3.74 (m, 4H), 3.08 – 2.82 (m, 2H), 1.99 – 1.66 (m, 2H), 1.30 (d, *J* = 7.0 Hz, 3H). MS(ESI): [M+H]^+^ m/z calcd. For C_31_H_33_N_2_O_5_S = 545.2, found = 545.5.

Compound **a23**

^1^H NMR (400 MHz, DMSO-d_6_) δ 12.46 (d, *J* = 2.7 Hz, 1H), 8.41 – 8.10 (m, 1H), 8.10 – 7.85 (m, 1H), 7.85 – 7.57 (m, 3H), 7.54 – 6.84 (m, 15H), 5.39 – 4.88 (m, 1H), 4.62 – 4.32 (m, 1H), 4.32 – 4.16 (m, 1H), 4.08 – 3.81 (m, 3H), 3.81 – 3.63 (m, 1H), 2.98 – 2.77 (m, 1H), 2.77 – 2.62 (m, 1H), 1.24 (d, *J* = 6.4 Hz, 3H). MS(ESI): [M+H]^+^ m/z calcd. For C_36_H_35_N_2_O_5_S = 607.2, found = 607.2.

Compound **a24**

^1^H NMR (400 MHz, DMSO-d_6_) δ 12.97 (s, 1H), 8.94 (dd, *J* = 41.0, 8.5 Hz, 1H), 8.49 (s, 1H), 8.40 (dd, *J* = 8.2, 4.6 Hz, 1H), 8.31 (d, *J* = 2.3 Hz, 1H), 8.07 – 7.87 (m, 2H), 7.54 – 7.26 (m, 3H), 7.21 – 7.04 (m, 2H), 6.95 (d, *J* = 8.7 Hz, 1H), 6.80 (dd, *J* = 17.5, 8.7 Hz, 2H), 6.61 – 6.46 (m, 2H), 6.25 (dd, *J* = 15.4, 8.5 Hz, 1H), 5.06 – 4.90 (m, 1H), 4.62 (d, *J* = 2.6 Hz, 2H), 3.76 – 3.64 (m, 13H), 3.63 – 3.49 (m, 5H), 3.43 – 3.29 (m, 2H). MS(ESI): [M+H]^+^ m/z calcd. For C_37_H_38_N_5_O_8_S = 711.2, found =711.2.

Compound **a25**

^1^H NMR (400 MHz, DMSO-d_6_) δ 12.93 (s, 1H), 8.62 (d, *J* = 8.4 Hz, 1H), 8.20 (d, *J* = 8.9 Hz, 1H), 7.91 (dd, *J* = 20.2, 7.3 Hz, 2H), 7.62 (ddd, *J* = 21.4, 14.8, 7.8 Hz, 7H), 7.35 (dd, *J* = 18.3, 10.0 Hz, 7H), 7.23 (d, *J* = 18.6 Hz, 2H), 7.09 (dd, *J* = 8.8, 2.2 Hz, 1H), 4.85 – 4.45 (m, 2H), 4.02 – 3.65 (m, 4H), 3.19 (dd, *J* = 13.6, 4.2 Hz, 1H), 2.98 – 2.82 (m, 2H), 2.81 – 2.72 (m, 1H), 1.17 (d, *J* = 7.0 Hz, 3H). MS(ESI): [M+H]^+^ m/z calcd. For C_41_H_37_N_2_O_6_S = 685.2, found = 685.2.

Compound **a26**

^1^H NMR (400 MHz, DMSO-d_6_) δ 8.35 (d, *J* = 8.5 Hz, 1H), 8.20 (t, *J* = 5.7 Hz, 1H), 7.97 (dd, *J* = 7.3, 5.0 Hz, 2H), 7.79 – 7.58 (m, 4H), 7.46 – 7.23 (m, 6H), 7.12 (dd, *J* = 8.9, 2.5 Hz, 1H), 6.76 (d, *J* = 8.3 Hz, 1H), 6.46 (d, *J* = 2.3 Hz, 1H), 6.18 (dd, *J* = 8.3, 2.4 Hz, 1H), 4.72 (dd, *J* = 14.2, 8.4 Hz, 1H), 4.06 (d, *J* = 5.2 Hz, 2H), 3.93 – 3.71 (m, 6H), 3.68 (d, *J* = 7.9 Hz, 7H), 1.24 (d, J = 5.1 Hz, 3H). MS(ESI): [M+H]^+^ m/z calcd. For C_34_H_35_N_2_O_5_S = 583.2, found = 583.2.

Compound **a27**

^1^H NMR (400 MHz, DMSO-d_6_) δ 12.96 (s, 1H), 8.38 (dd, *J* = 15.9, 7.1 Hz, 2H), 8.02 – 7.93 (m, 2H), 7.80 – 7.66 (m, 3H), 7.49 – 7.34 (m, 4H), 7.26 (d, *J* = 2.4 Hz, 1H), 7.12 (dt, *J* = 8.0, 4.0 Hz, 1H), 6.96 (d, *J* = 8.6 Hz, 2H), 6.71 (d, *J* = 8.7 Hz, 2H), 4.77 – 4.56 (m, 3H), 4.11 (d, *J* = 5.7 Hz, 2H), 3.92 – 3.75 (m, 4H), 3.26 (dd, *J* = 14.4, 5.9 Hz, 1H), 3.11 (dd, *J* = 14.4, 8.6 Hz, 1H), 1.26 (t, *J* = 7.9 Hz, 3H). MS(ESI): [M+H]^+^ m/z calcd. For C_34_H_3_HN_2_O_6_S = 597.2, found = 597.2.

Compound **a28**

^1^H NMR (400 MHz, DMSO-d_6_) δ 8.57 (d, *J* = 39.0 Hz, 2H), 8.02 – 7.83 (m, 2H), 7.61 – 7.26 (m, 9H), 7.19 (t, *J* = 8.6 Hz, 1H), 7.07 (dd, *J* = 12.5, 6.9 Hz, 2H), 6.96 (d, *J* = 8.6 Hz, 1H), 6.69 (dd, *J* = 20.9, 8.6 Hz, 2H), 4.64 (dd, *J* = 24.6, 5.3 Hz, 1H), 4.27 – 4.10 (m, 2H), 4.02 (d, *J* = 11.0 Hz, 2H), 3.77 (dd, *J* = 7.0, 3.0 Hz, 1H), 1.26 (dd, *J* = 43.9, 7.0 Hz, 3H). MS(ESI): [M+H]^+^ m/z calcd. For C_35_H_32_FN_2_O_5_S = 611.2, found = 611.

Compound **a29**

^1^H NMR (400 MHz, DMSO-d_6_) δ 8.29 – 8.17 (m, 2H), 7.95 (dd, *J* = 13.2, 7.5 Hz, 2H), 7.45 – 7.34 (m, 3H), 7.13 (d, *J* = 8.7 Hz, 2H), 6.91 (d, *J* = 8.6 Hz, 2H), 6.83 (d, *J* = 8.7 Hz, 2H), 6.68 (d, *J* = 8.6 Hz, 2H), 4.66 (d, *J* = 5.9 Hz, 1H), 4.38 (s, 2H), 4.20 (dd, *J* = 15.0, 6.2 Hz, 1H), 4.04 (dd, *J* = 15.0, 5.4 Hz, 1H), 3.58 (s, 1H), 3.28 – 3.23 (m, 1H), 3.09 (dd, *J* = 11.8, 5.9 Hz, 5H), 2.70 – 2.62 (m, 4H), 2.34 (s, 3H), 1.14 (d, *J* = 7.0 Hz, 3H). MS(ESI): [M+H]^+^ m/z calcd. For C_34_H_39_N_4_O_5_S = 615.3, found = 615.

Compound **a30**

^1^H NMR (400 MHz, CDCl_3_) δ 7.90 (d, *J* = 7.3 Hz, 1H), 7.84 (d, *J* = 7.2 Hz, 1H), 7.64 (dd, *J* = 8.6, 6.5 Hz, 2H), 7.56 (s, 1H), 7.37 (d, *J* = 1.7 Hz, 2H), 7.24 (d, *J* = 1.3 Hz, 1H), 7.15 (dd, *J* = 8.9, 2.5 Hz, 1H), 7.10 (d, *J* = 2.4 Hz, 1H), 6.92 (s, 1H), 6.82 (d, *J* = 8.6 Hz, 2H), 6.72 (d, *J* = 8.6 Hz, 2H), 6.16 (d, *J* = 7.5 Hz, 1H), 5.69 (s, 1H), 4.68 (d, *J* = 6.1 Hz, 1H), 4.09 (d, *J* = 5.6 Hz, 2H), 3.93 (s, 3H), 3.65 (d, *J* = 7.1 Hz, 1H), 3.32 (dd, *J* = 14.3, 6.0 Hz, 1H), 3.28 – 3.10 (m, 5H), 2.66 (s, 4H), 2.42 (s, 3H), 1.56 (d, *J* = 7.2 Hz, 3H). MS(ESI): [M+H]^+^ m/z calcd. For C_37_H_40_N_4_O_3_S = 621.3, found = 621.

Compound **a31**

^1^H NMR (400 MHz, DMSO-d_6_) δ 8.74 (ddd, *J* = 29.6, 25.5, 8.3 Hz, 1H), 7.96 – 7.20 (m, 5H), 7.16 – 6.75 (m, 5H), 6.63 – 6.37 (m, 2H), 6.35 – 5.91 (m, 1H), 4.88 – 4.48 (m, 3H), 4.28 – 4.01 (m, 1H), 3.93 – 3.53 (m, 10H), 2.83 (dd, *J* = 24.1, 12.2 Hz, 3H), 2.16 – 1.97 (m, 1H), 1.37 – 1.24 (m, 3H), 1.03 – 0.39 (m, 6H). MS(ESI): [M+H]^+^ m/z calcd. For C_37_H_43_N_2_O_8_ = 643.3, found = 643.4.

Compound **a32**

^1^H NMR (400 MHz, DMSO-d_6_) δ 13.03 (s, 1H), 8.47 (ddd, *J* = 28.8, 13.2, 6.6 Hz, 1H), 8.10 – 7.49 (m, 4H), 7.47 – 6.20 (m, 15H), 6.19 – 5.55 (m, 1H), 5.27 – 4.07 (m, 7H), 4.06 – 3.63 (m, 10H), 3.17 – 2.84 (m, 2H), 1.40 (t, *J* = 6.0 Hz, 3H). MS(ESI): [M+Na]^+^ m/z calcd. For C_41_H_40_N_2_NaO_8_ = 711.3, found = 711.4.

Compound **a33**

^1^H NMR (400 MHz, DMSO-d_6_) δ 13.01 (s, 1H), 9.10 – 8.23 (m, 1H), 8.13 – 7.07 (m, 16H), 7.07 – 6.95 (m, 1H), 6.93 – 6.75 (m, 2H), 6.69 – 6.34 (m, 3H), 6.21 (t, *J* = 9.1 Hz, 1H), 4.87 – 4.39 (m, 4H), 4.17 (qd, *J* = 17.9, 8.9 Hz, 2H), 3.94 – 3.80 (m, 4H), 3.80 – 3.69 (m, 6H), 3.32 – 3.03 (m, 2H), 2.85 (td, *J* = 10.7, 5.4 Hz, 1H), 1.82 (tdd, *J* = 33.3, 21.5, 12.1 Hz, 1H), 1.39 – 1.17 (m, 3H). MS(ESI): [M+H]^+^ m/z calcd. For C_42_H_43_N_2_O_8_ = 703.3, found = 703.3.

Compound **a34**

^1^H NMR (400 MHz, DMSO-d_6_) δ 13.00 (s, 1H), 9.69 (d, *J* = 65.5 Hz, 1H), 8.41 (ddd, *J* = 32.3, 16.1, 5.3 Hz, 1H), 7.95 – 7.56 (m, 3H), 7.50 – 7.31 (m, 1H), 7.27 (s, 1H), 7.07 (dddd, *J* = 14.4, 11.0, 6.9, 2.5 Hz, 4H), 6.92 – 6.73 (m, 3H), 6.38 (ddddd, *J* = 14.4, 10.8, 8.3, 7.2, 2.9 Hz, 5H), 4.82 – 4.50 (m, 3H), 4.36 – 3.94 (m, 3H), 3.93 – 3.80 (m, 4H), 1.37 – 1.23 (m, 3H). MS(ESI): [M+H]^+^ m/z calcd. For C_41_H_43_N_2_O_10_ = 723.3, found = 723.2.

**3.3 ^1^H-NMR and mass spectra (MS) of compounds**


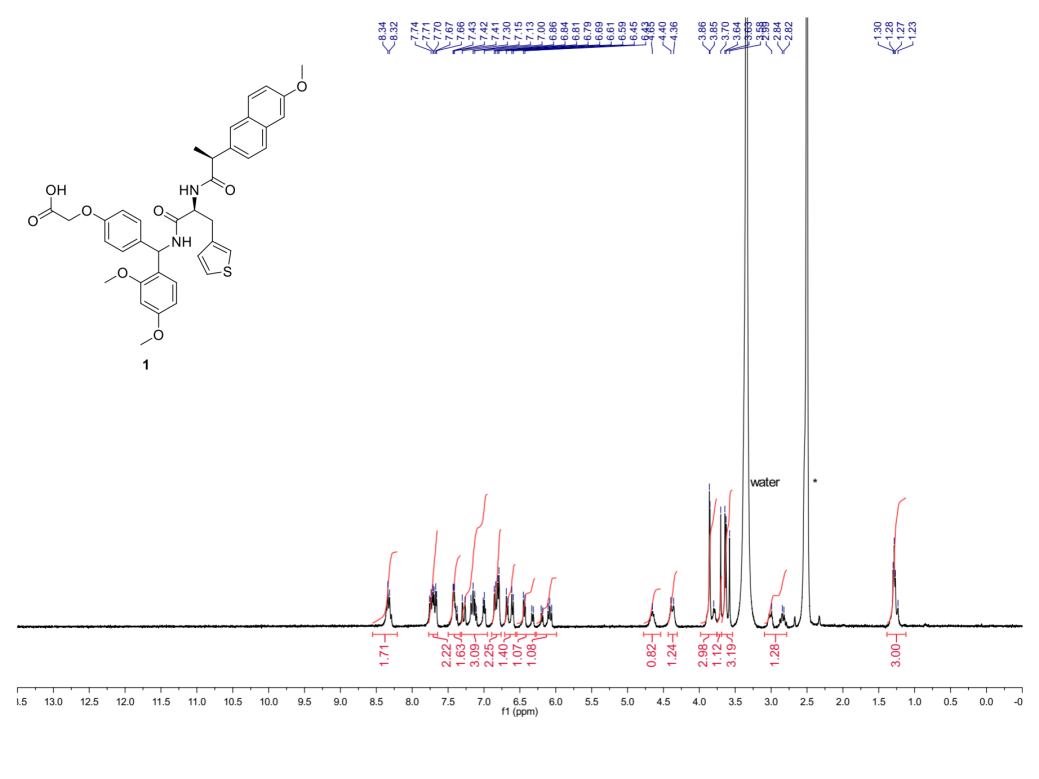


Figure **S2.** ^1^H-NMR spectrum of compound **1** in DMSO-d_6_.


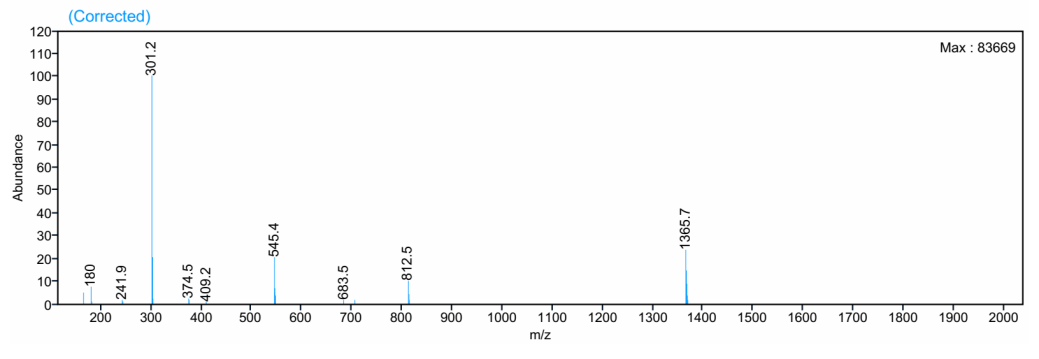


Figure **S3.** ESI mass spectrum of compound **1**.


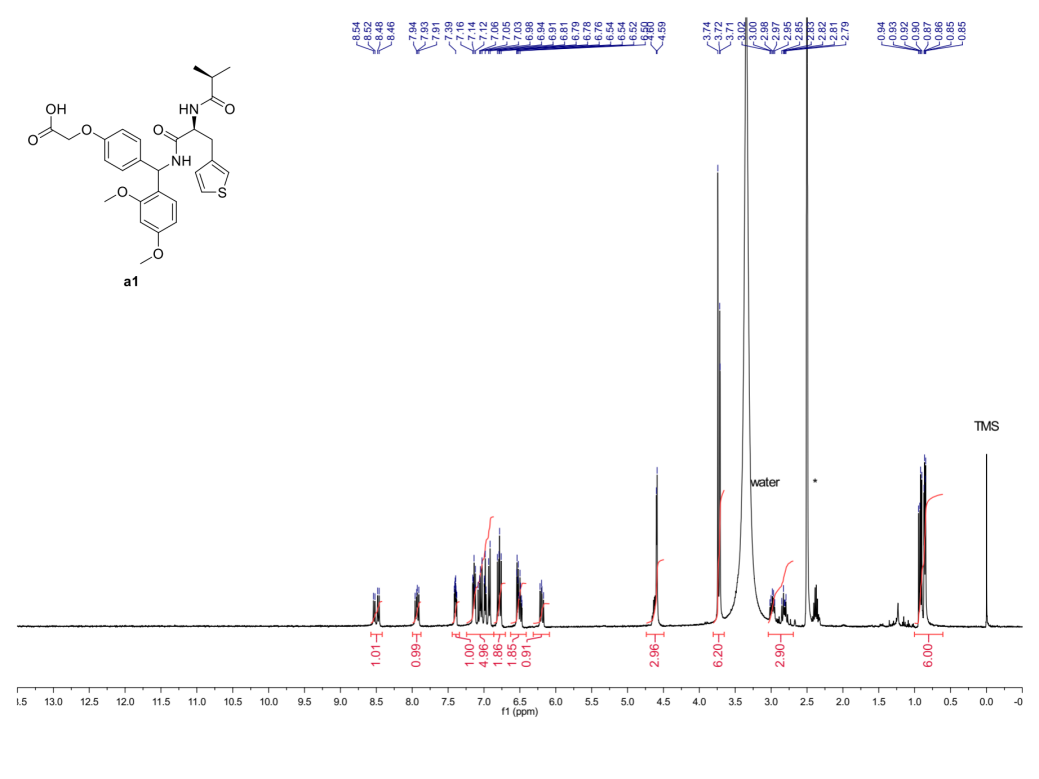


Figure **S4.** ^1^H-NMR spectrum of compound **a1** in DMSO-d_6_.


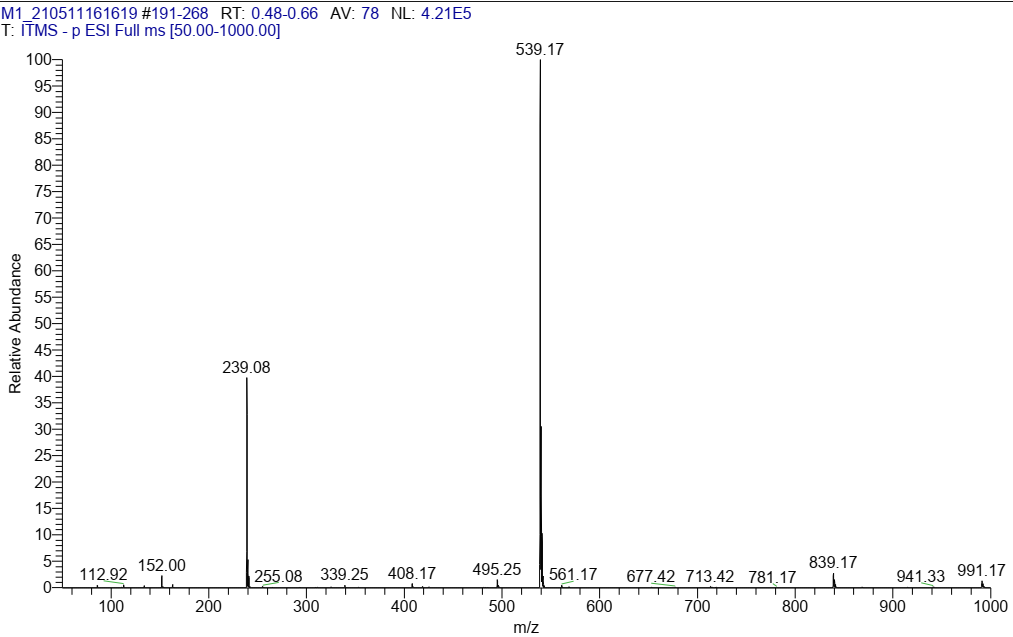


Figure **S5.** ESI mass spectrum of compound **a1**.


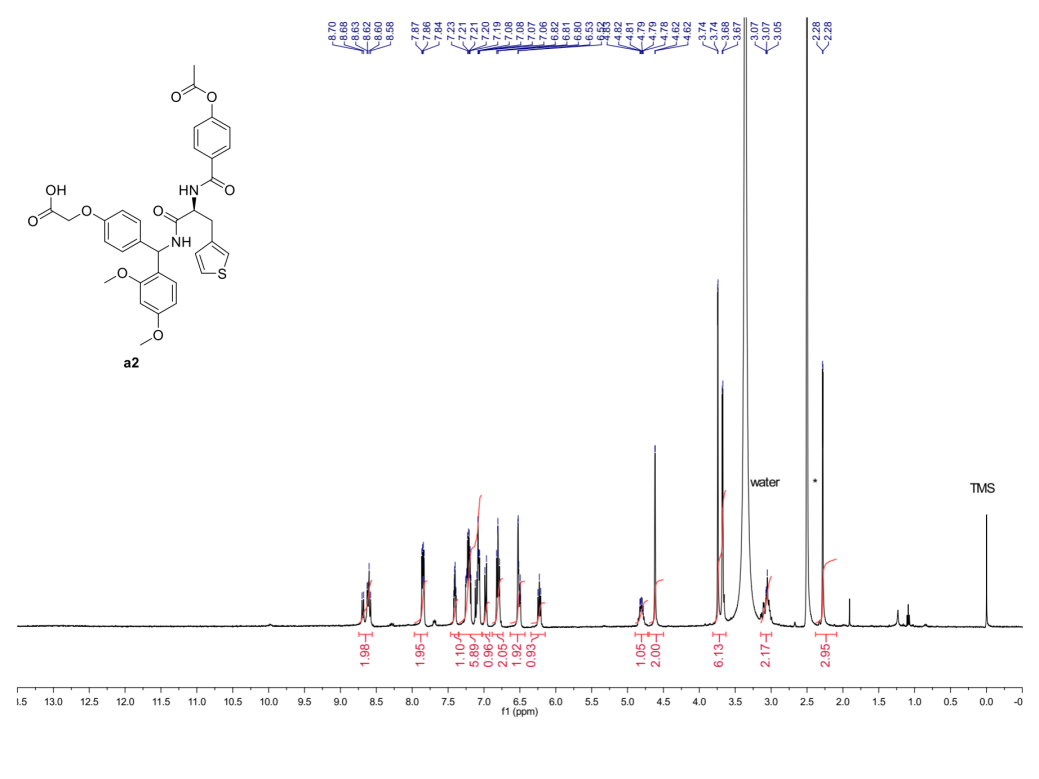


Figure **S6.** ^1^H-NMR spectrum of compound **a2** in DMSO-d_6_.


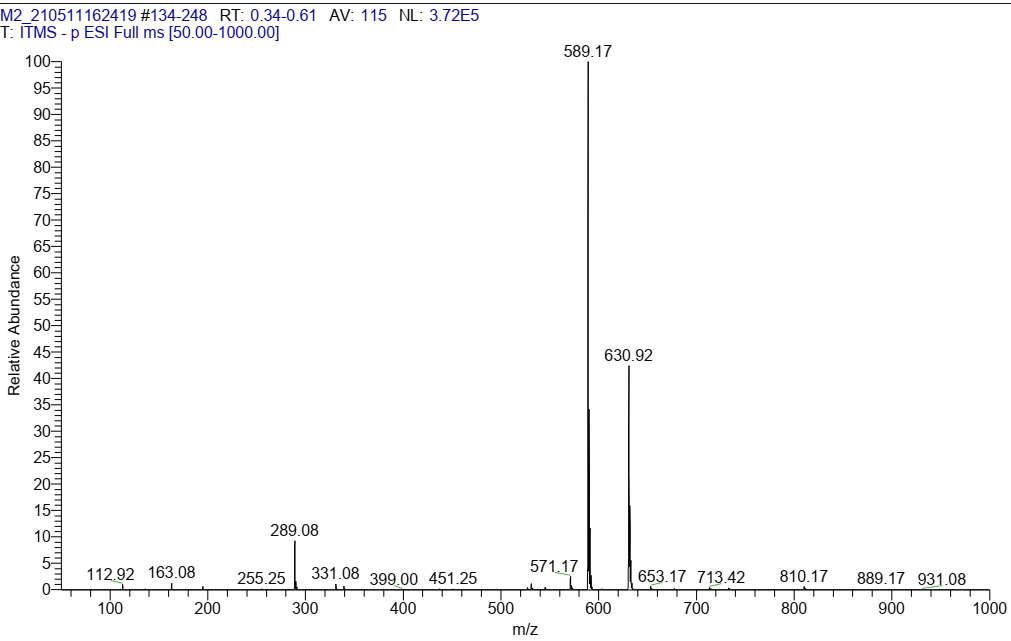


Figure **S7.** ESI mass spectrum of compound **a2**.


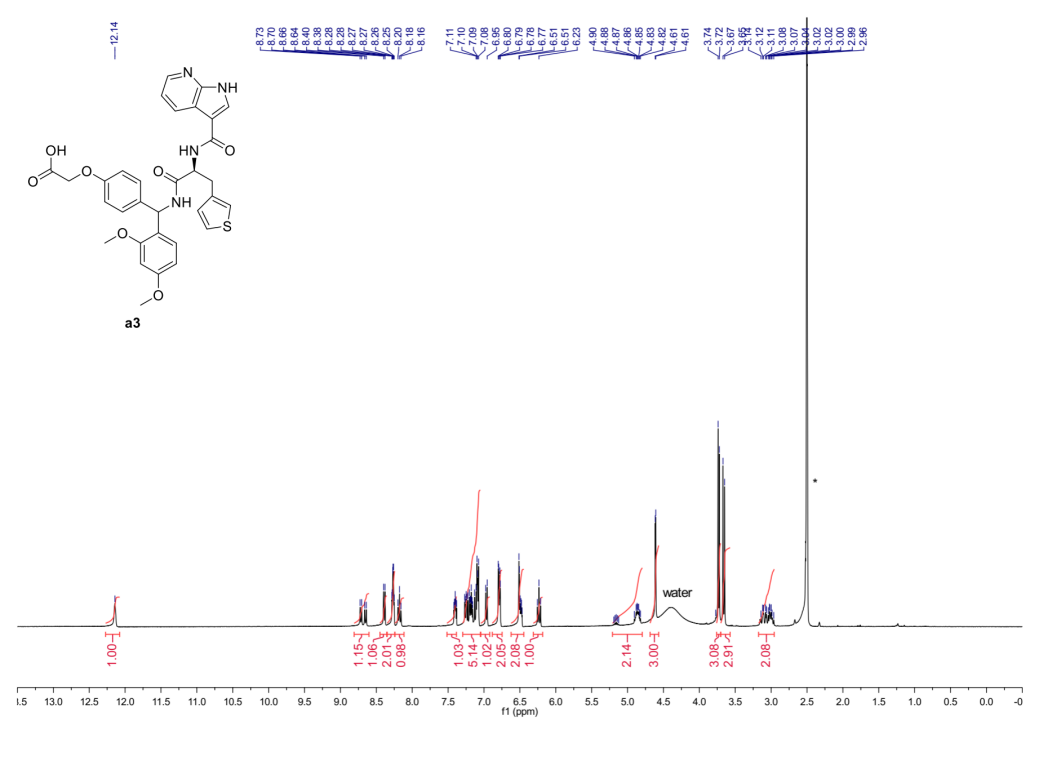


Figure **S8.** ^1^H-NMR spectrum of compound **a3** in DMSO-d_6_.


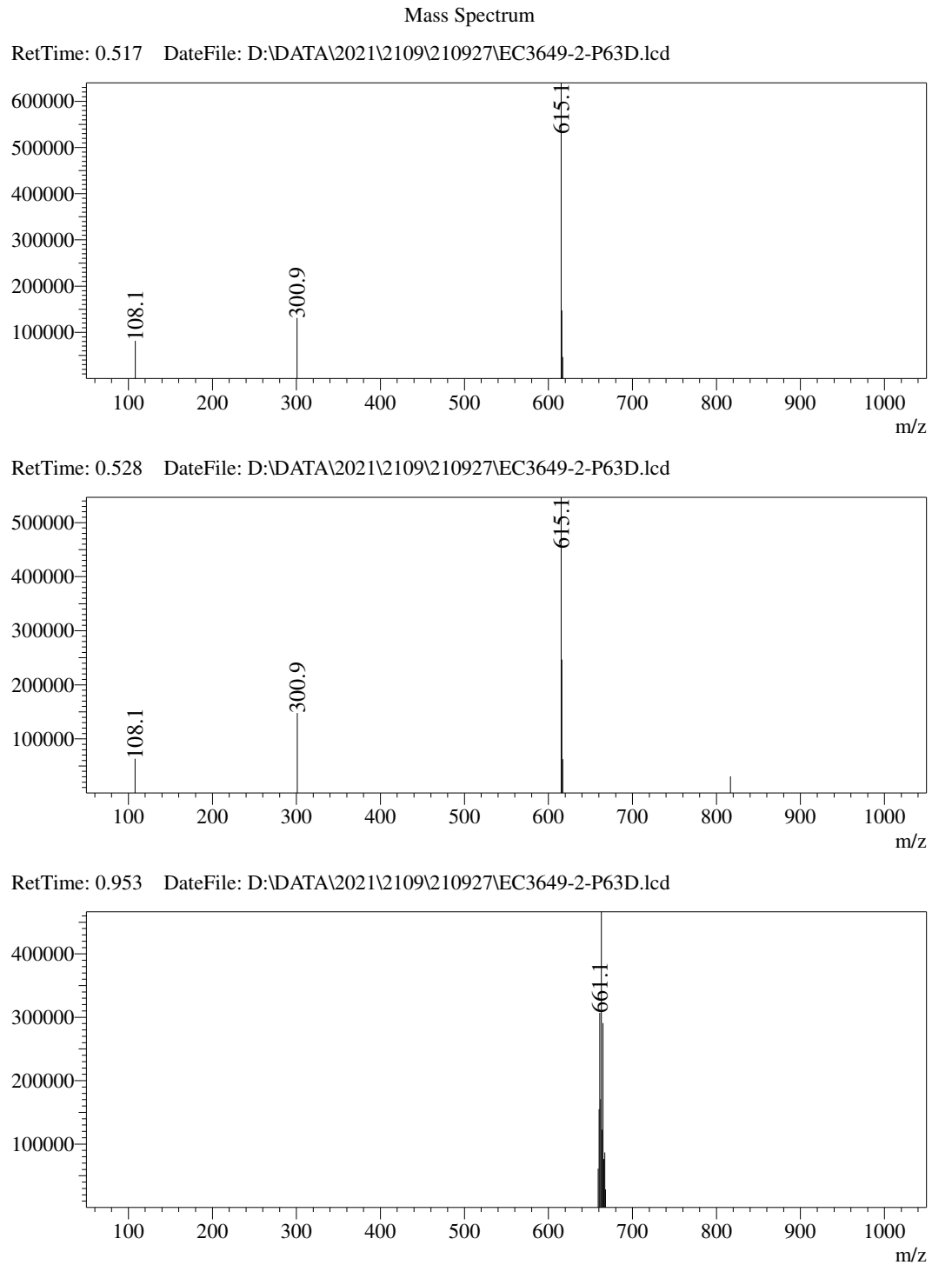


Figure **S9.** ESI mass spectrum of compound **a3**.


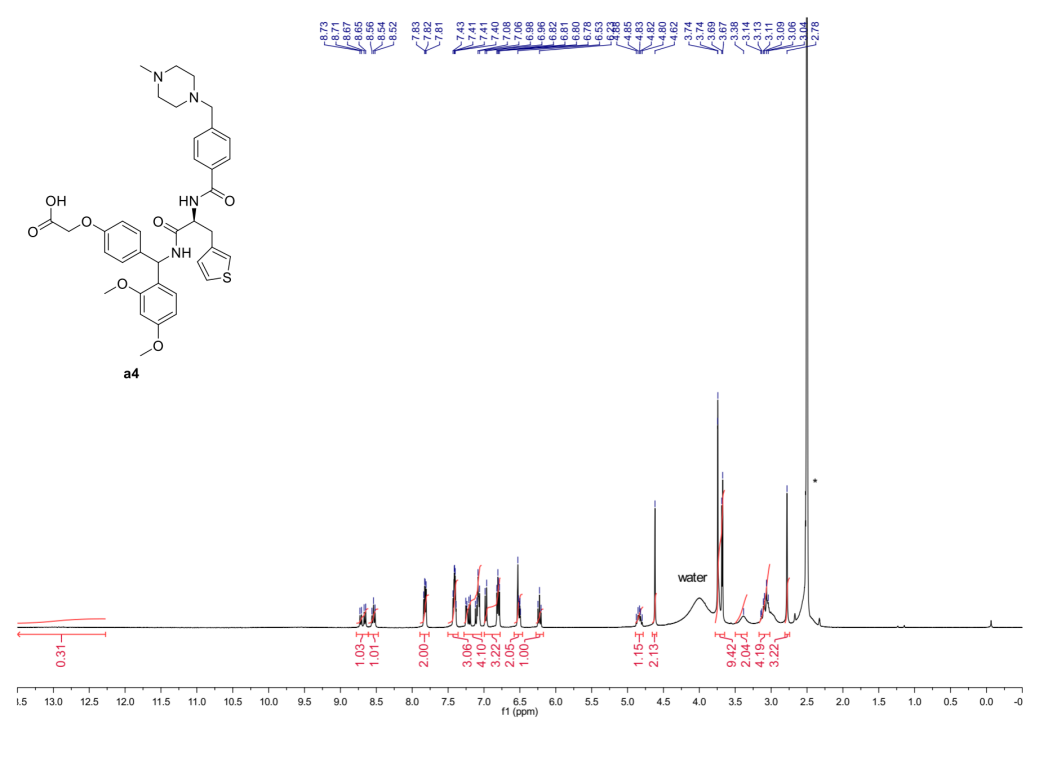


Figure **S10.** ^1^H-NMR spectrum of compound **a4** in DMSO-d_6_.


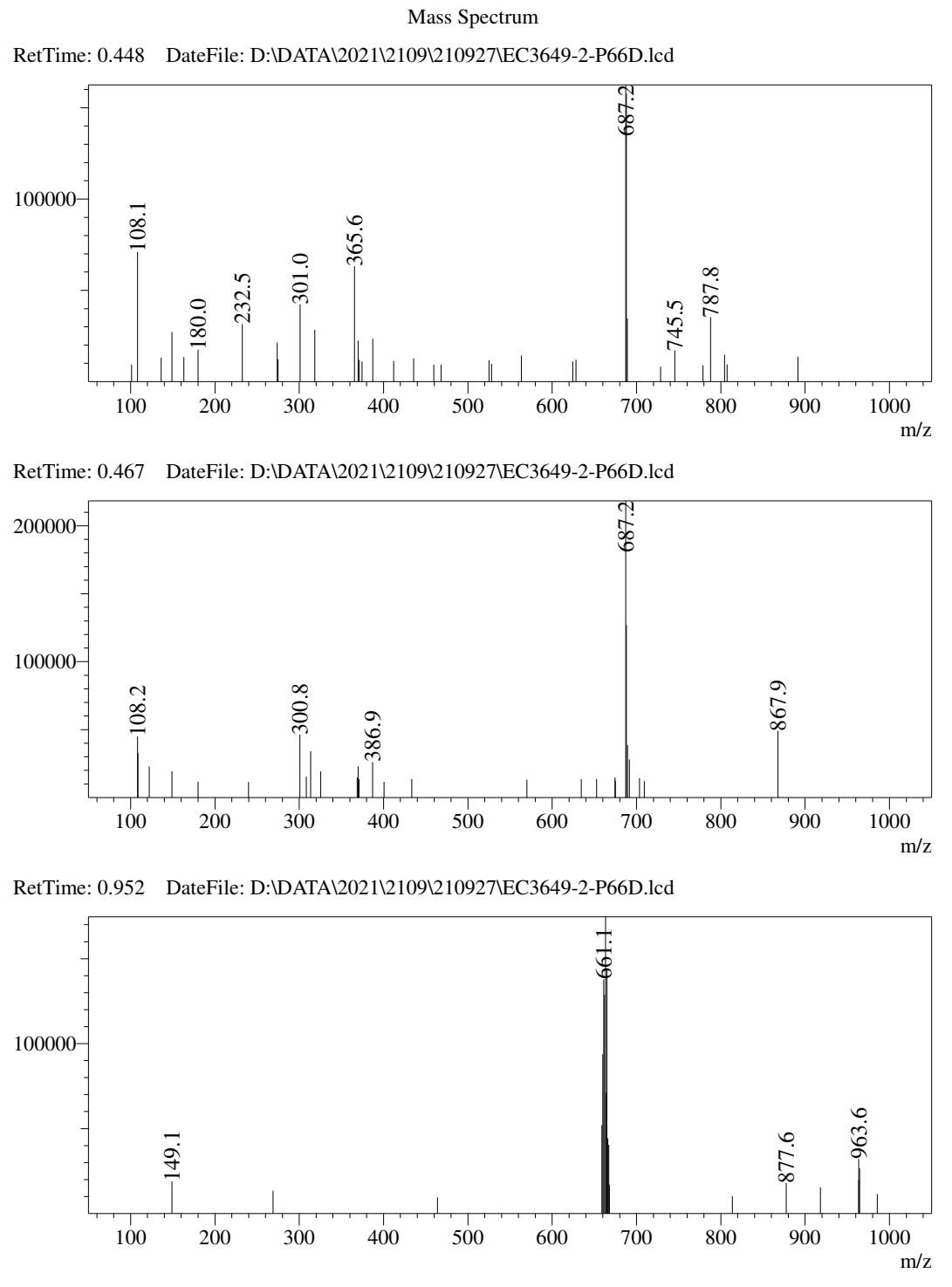


Figure **S11.** ESI mass spectrum of compound **a4**.


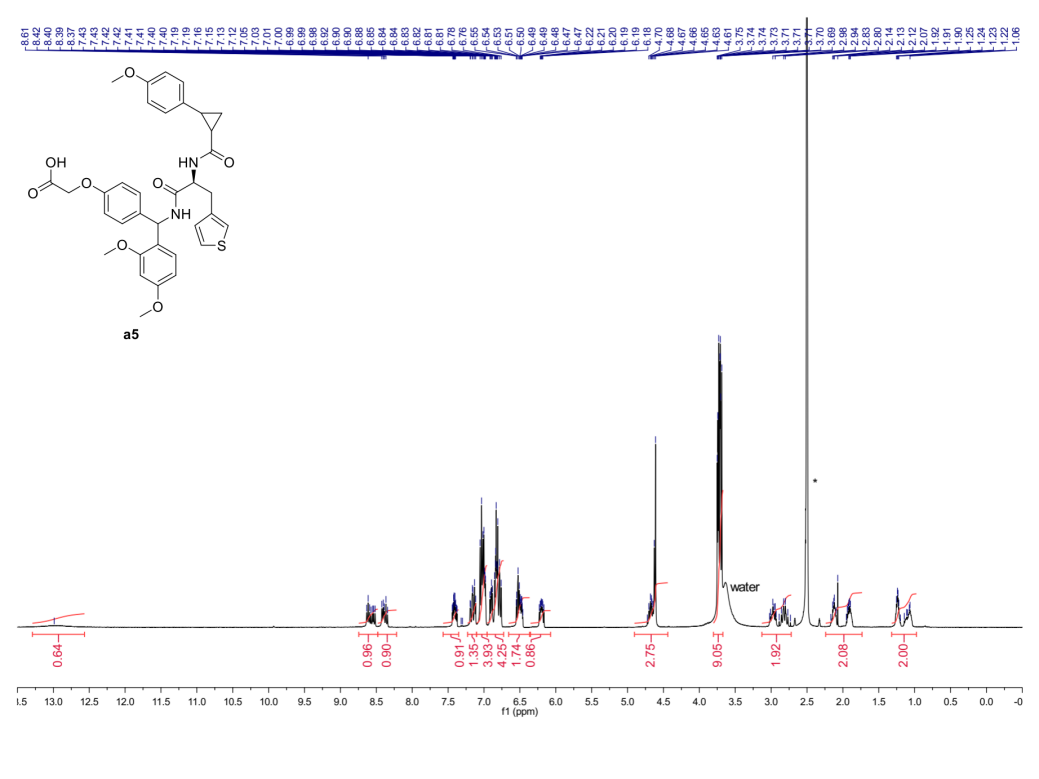


Figure **S12.** ^1^H-NMR spectrum of compound **a5** in DMSO-d_6_.


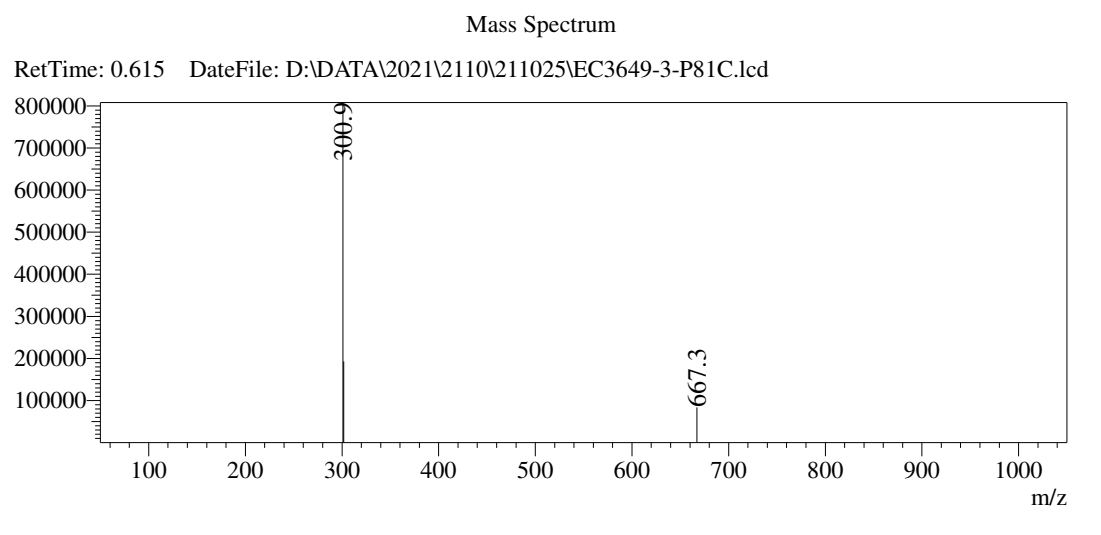


Figure **S13.** ESI mass spectrum of compound **a5**.


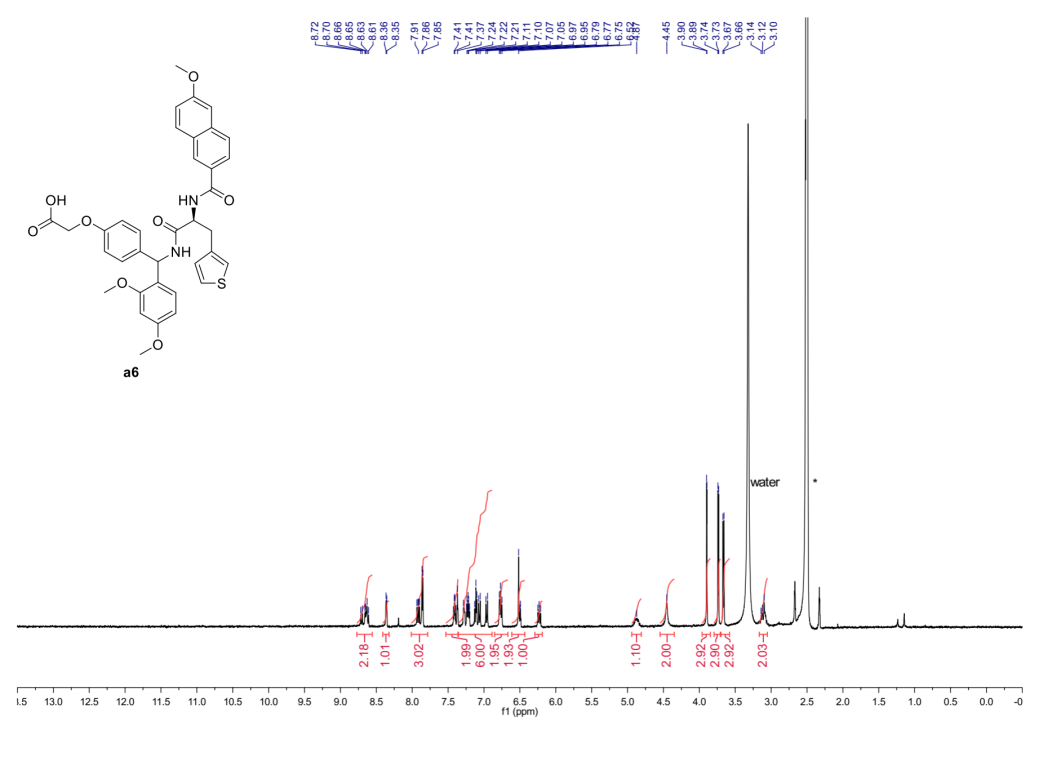


Figure **S14.** ^1^H-NMR spectrum of compound **a6** in DMSO-d_6_.


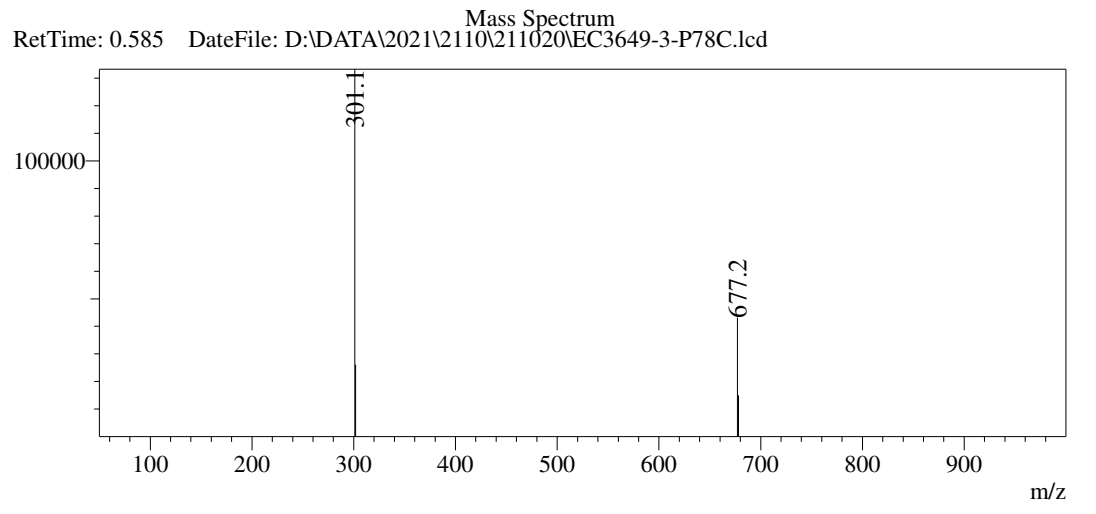


Figure **S15.** ESI mass spectrum of compound **a6**.


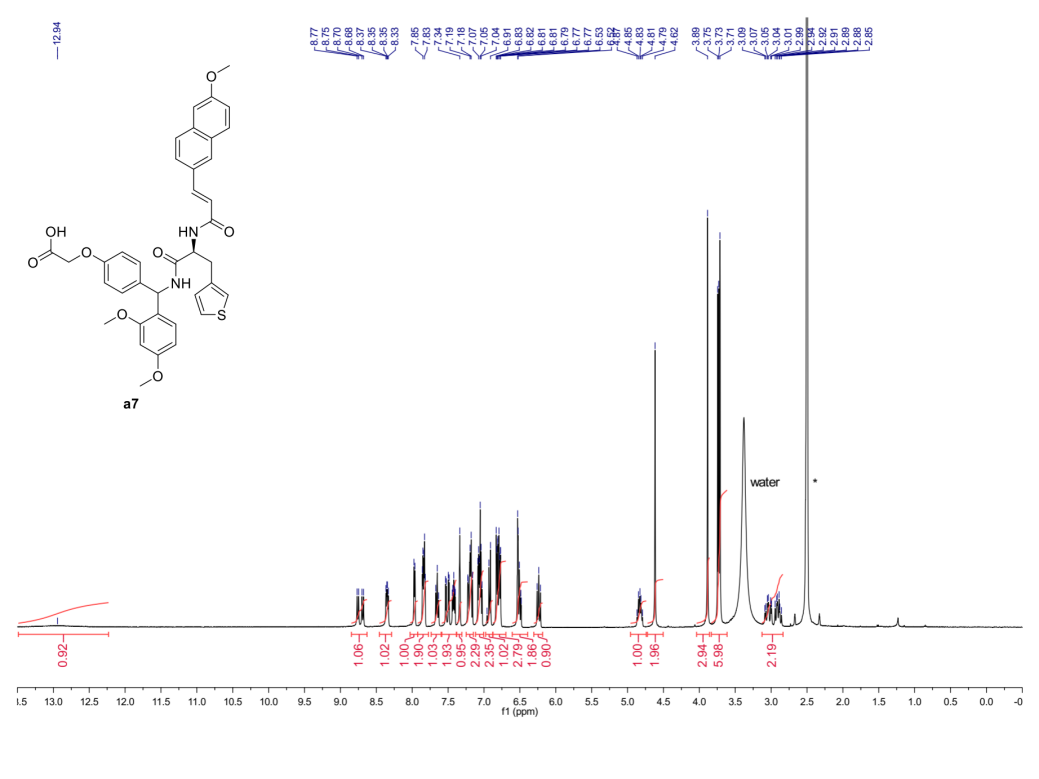


Figure **S16.** ^1^H-NMR spectrum of compound **a7** in DMSO-d_6_.


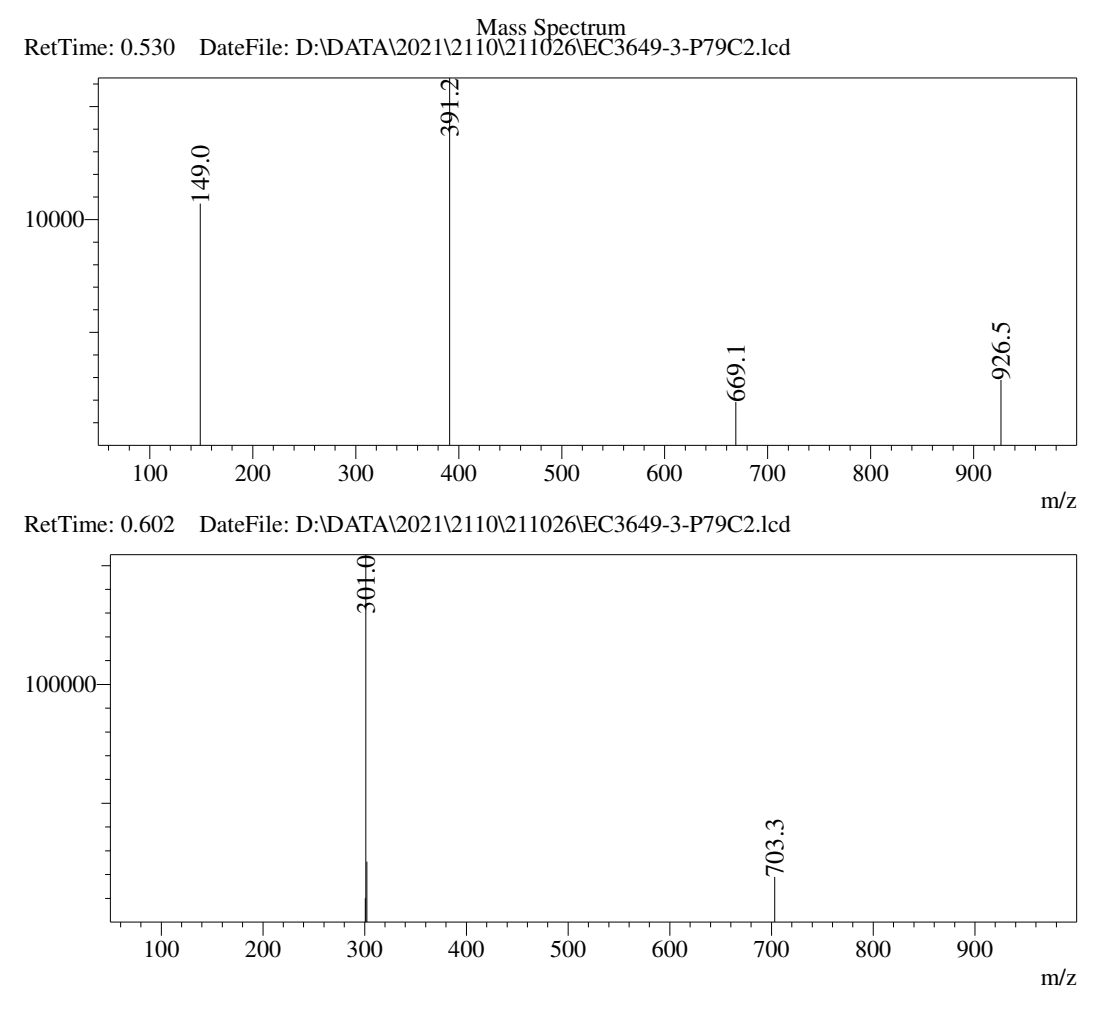


Figure **S17.** ESI mass spectrum of compound **a7**.


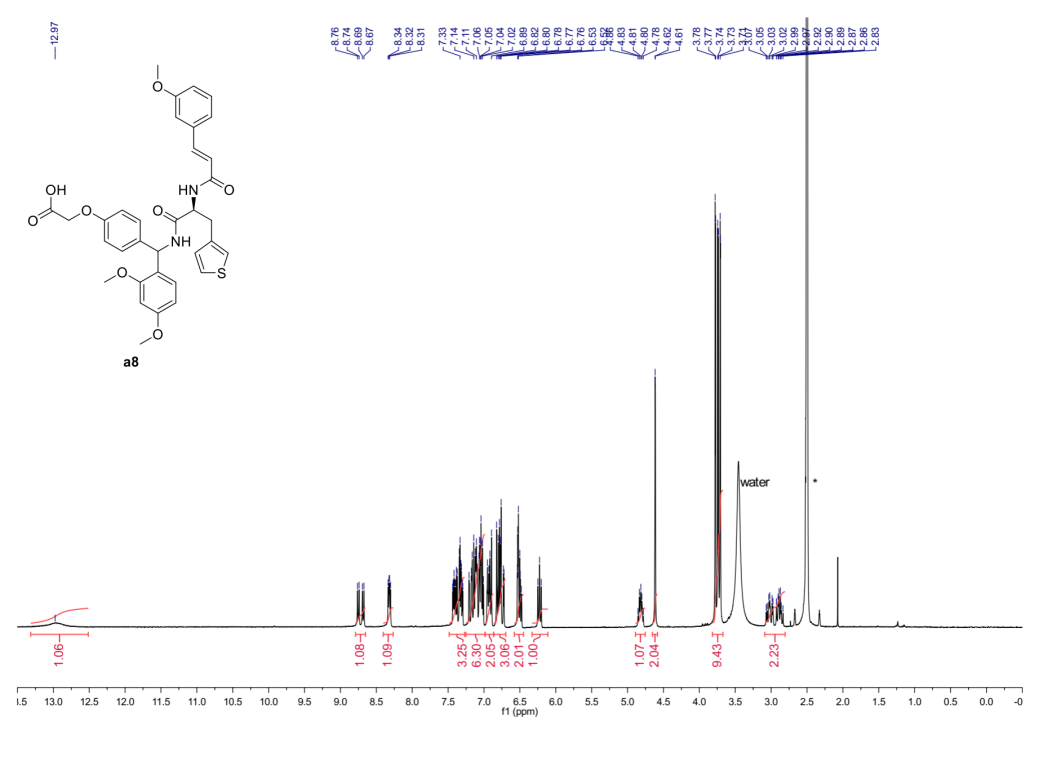


Figure **S18.** ^1^H-NMR spectrum of compound **a8** in DMSO-d_6_.


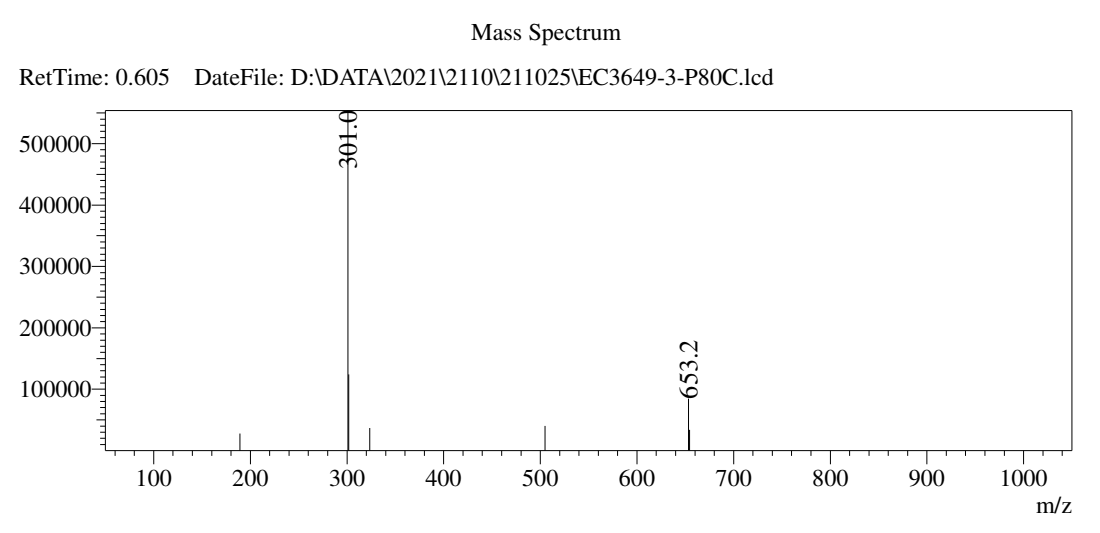


Figure **S19.** ESI mass spectrum of compound **a8**.


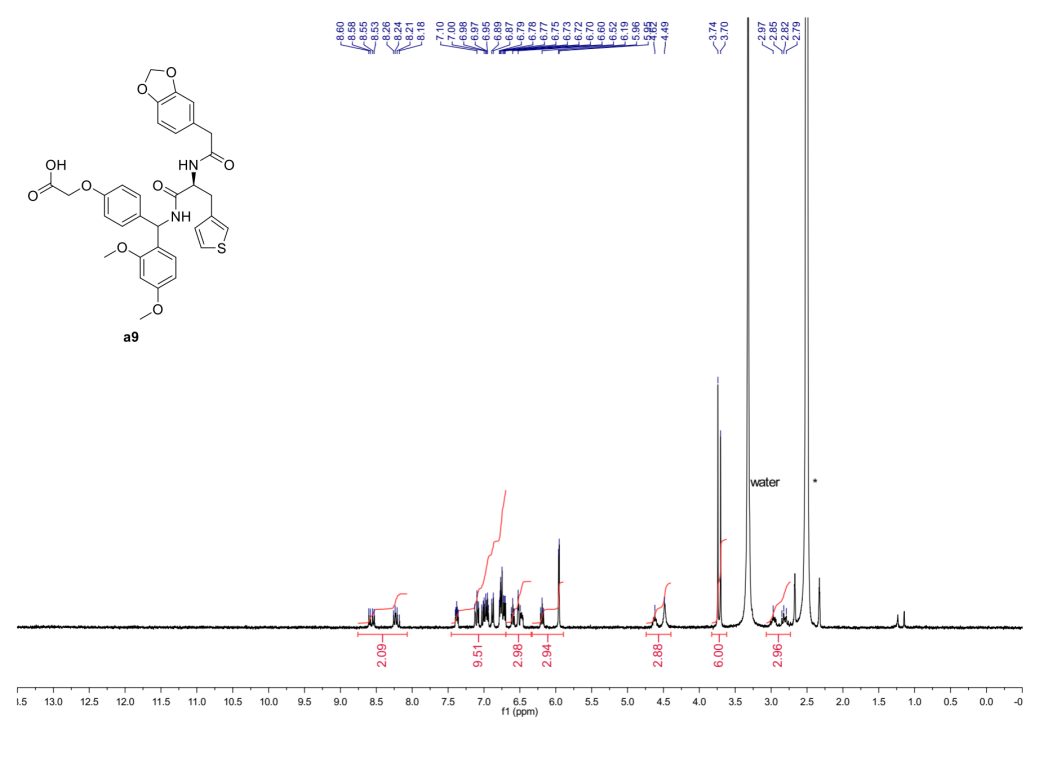


Figure **S20.** ^1^H-NMR spectrum of compound **a9** in DMSO-d_6_.


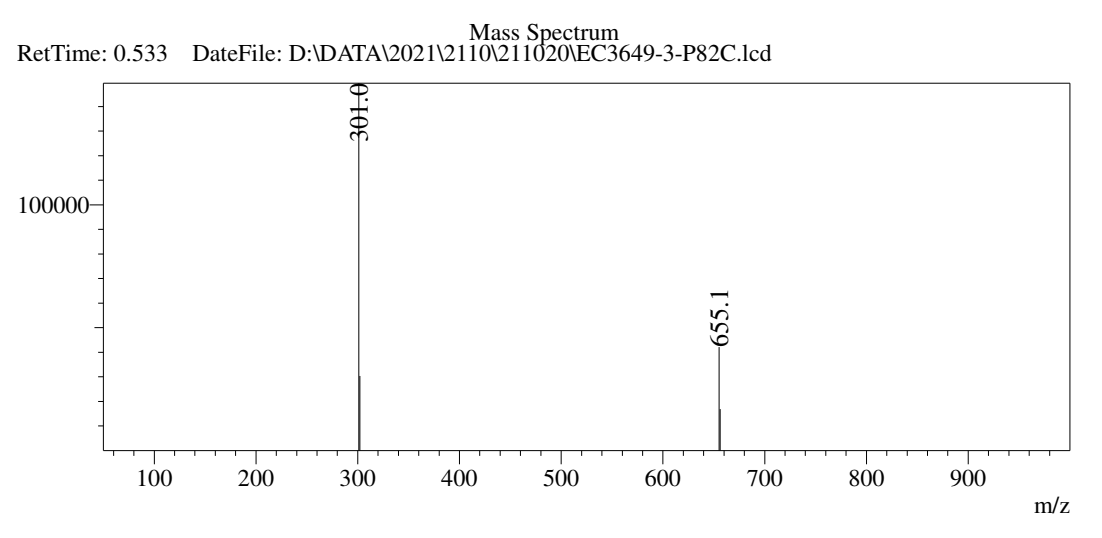


Figure **S21.** ESI mass spectrum of compound **a9**.


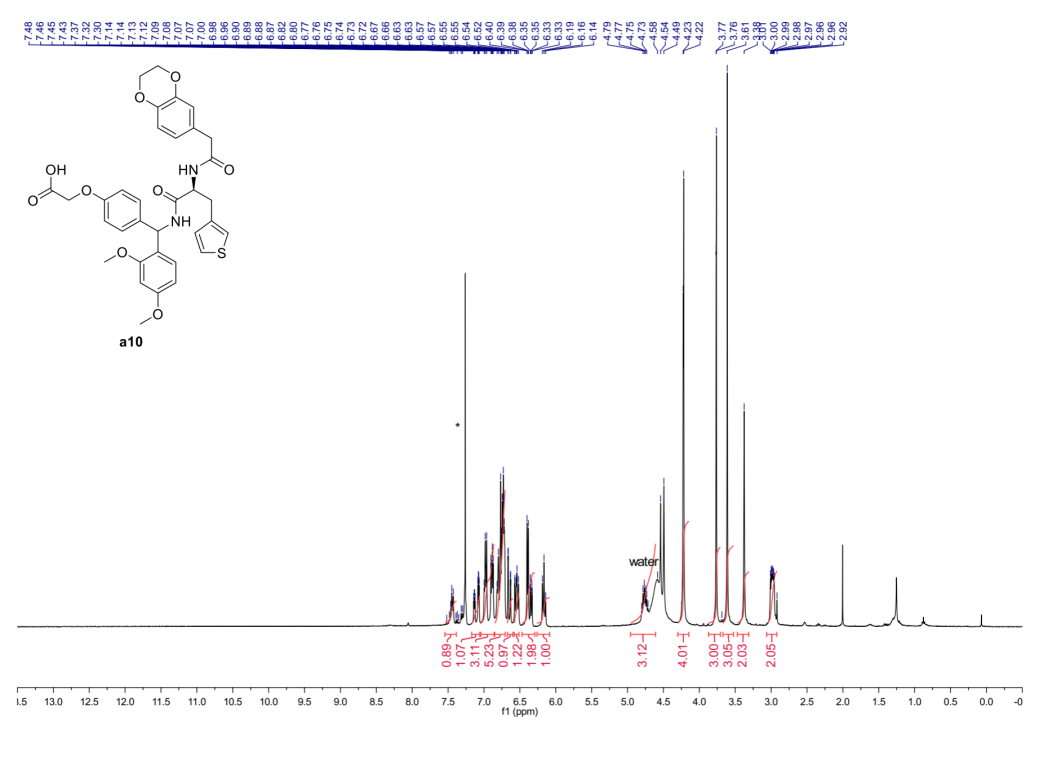


Figure **S22.** ^1^H-NMR spectrum of compound **a10** in CDCl_3_.


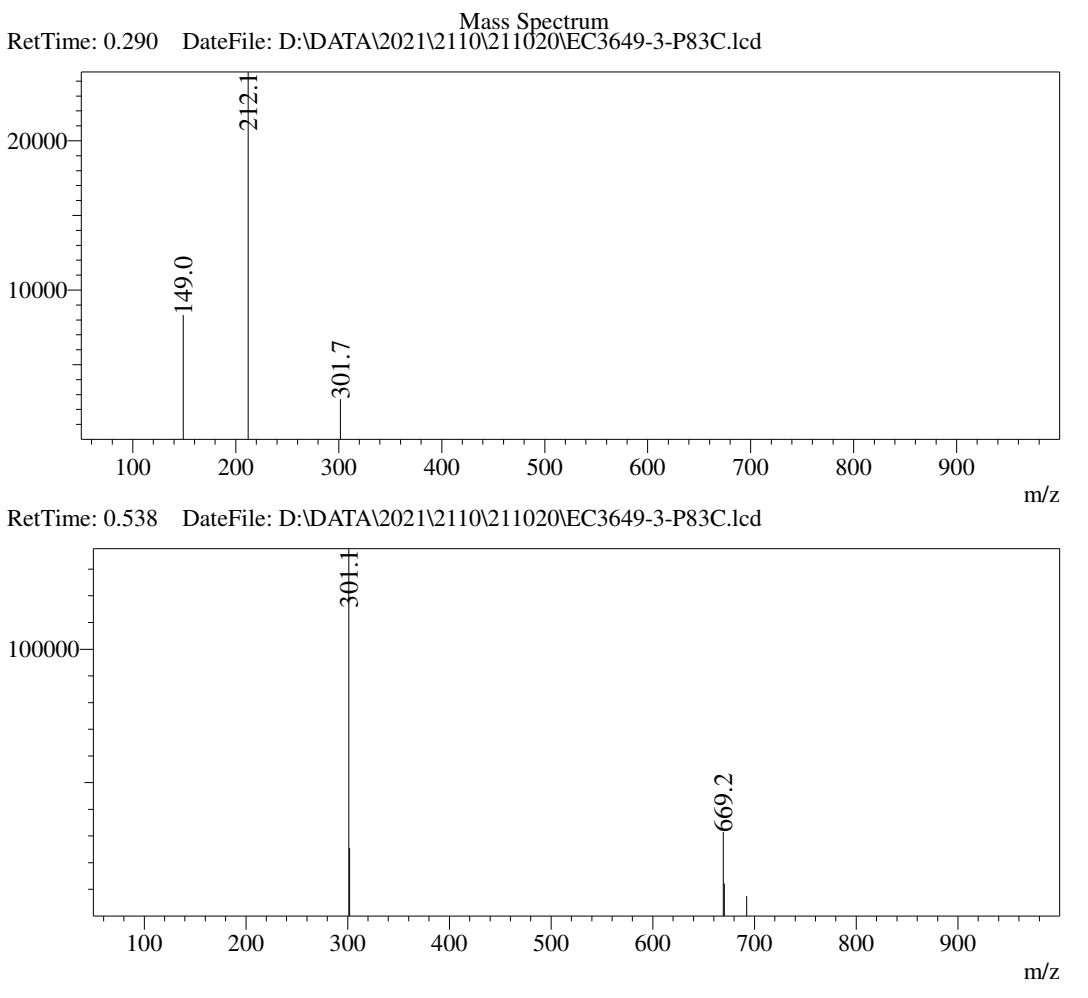


Figure **S23.** ESI mass spectrum of compound **a10**.


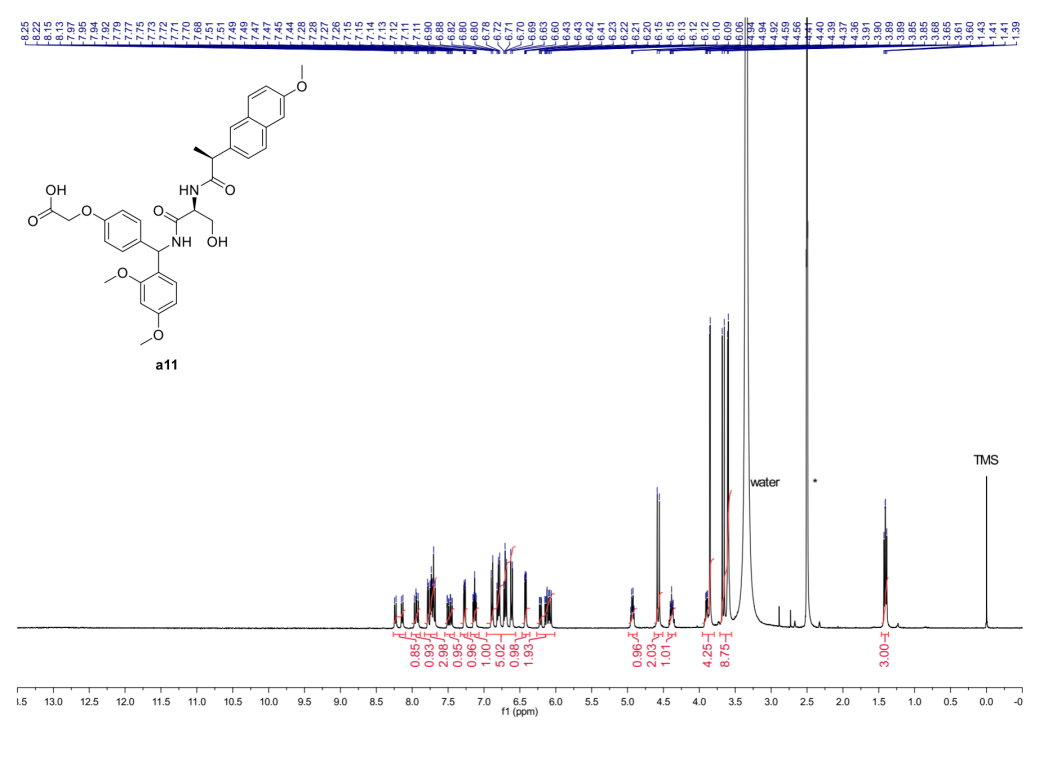


Figure **S24.** ^1^H-NMR spectrum of compound **a11** in DMSO-d_6_.


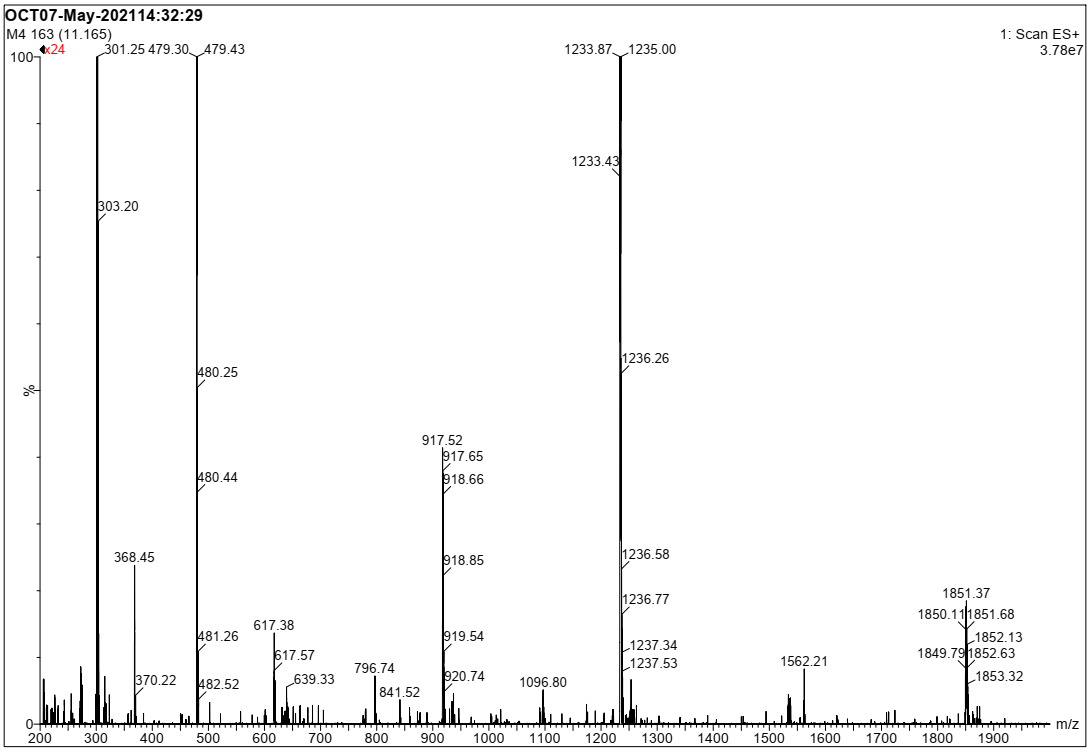


Figure **S25.** ESI mass spectrum of compound **a11**.


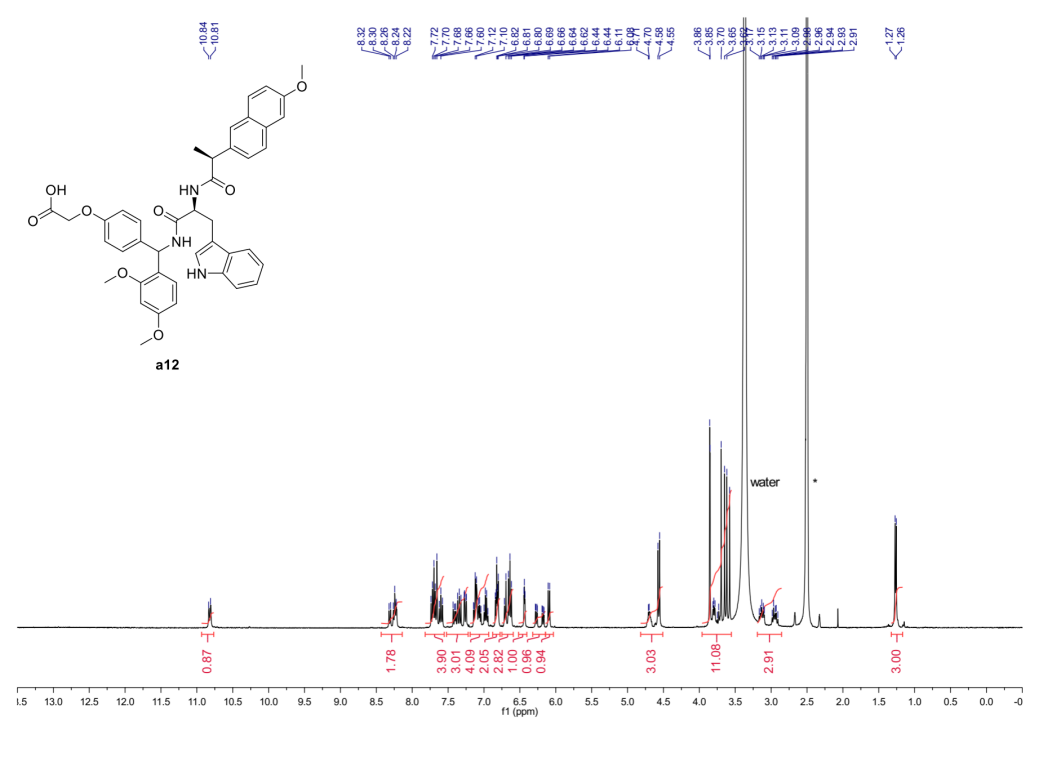


Figure **S26.** ^1^H-NMR spectrum of compound **a12** in DMSO-d_6_.


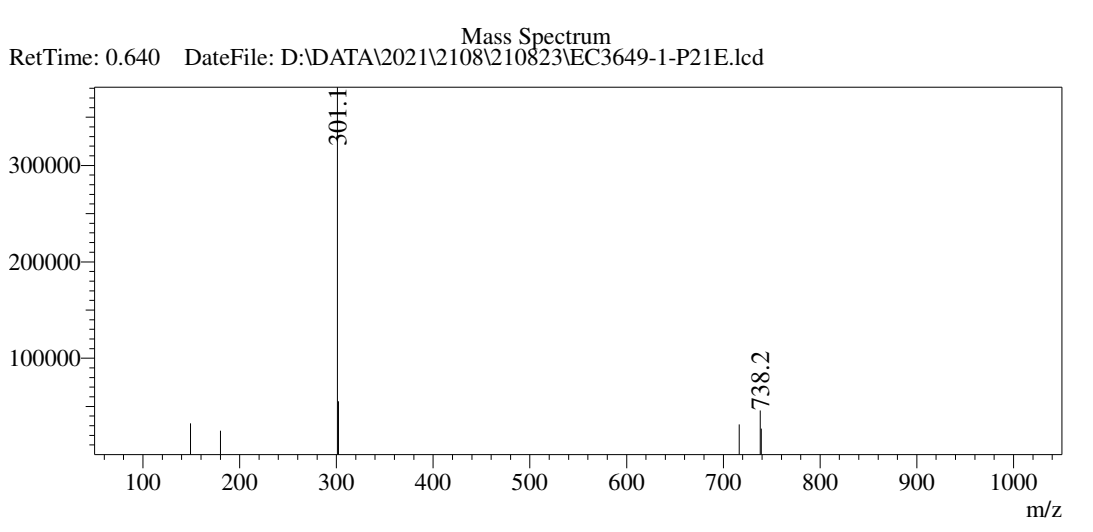


Figure **S27.** ESI mass spectrum of compound **a12**.


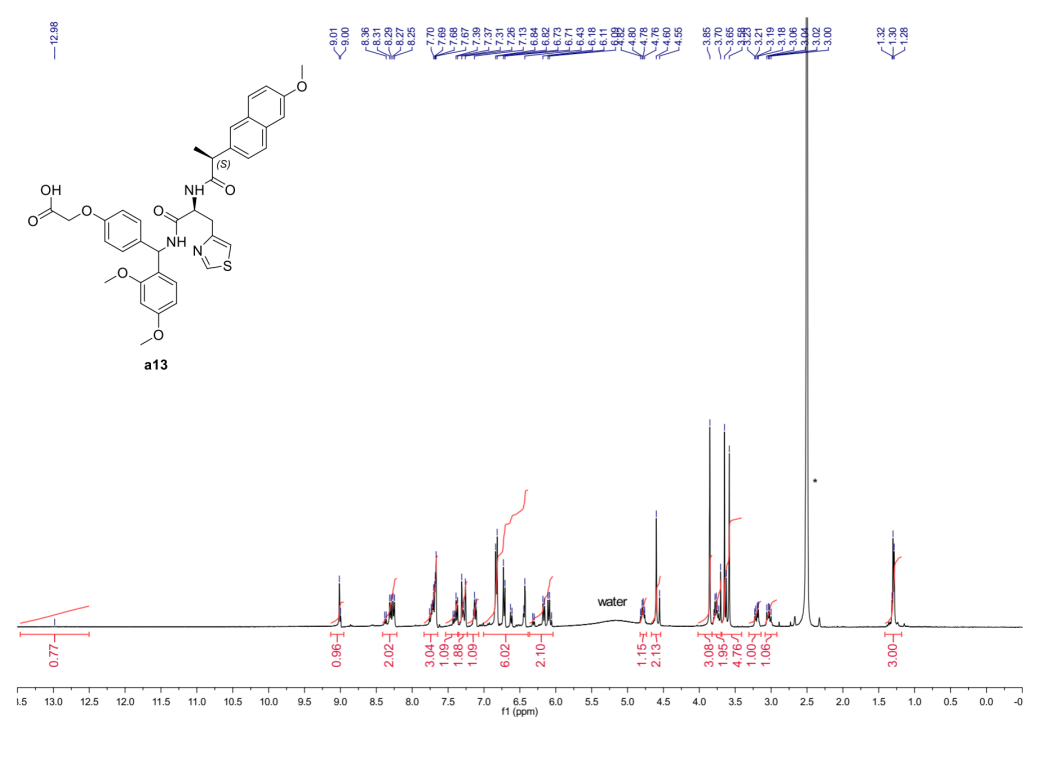


Figure **S28.** ^1^H-NMR spectrum of compound **a13** in DMSO-d_6_.


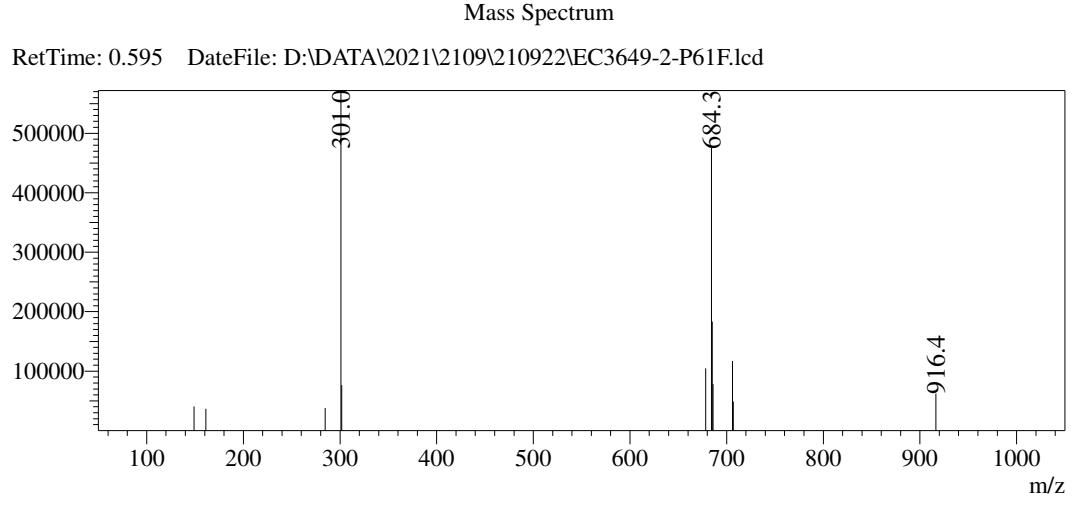


Figure **S29.** ESI mass spectrum of compound **a13**.


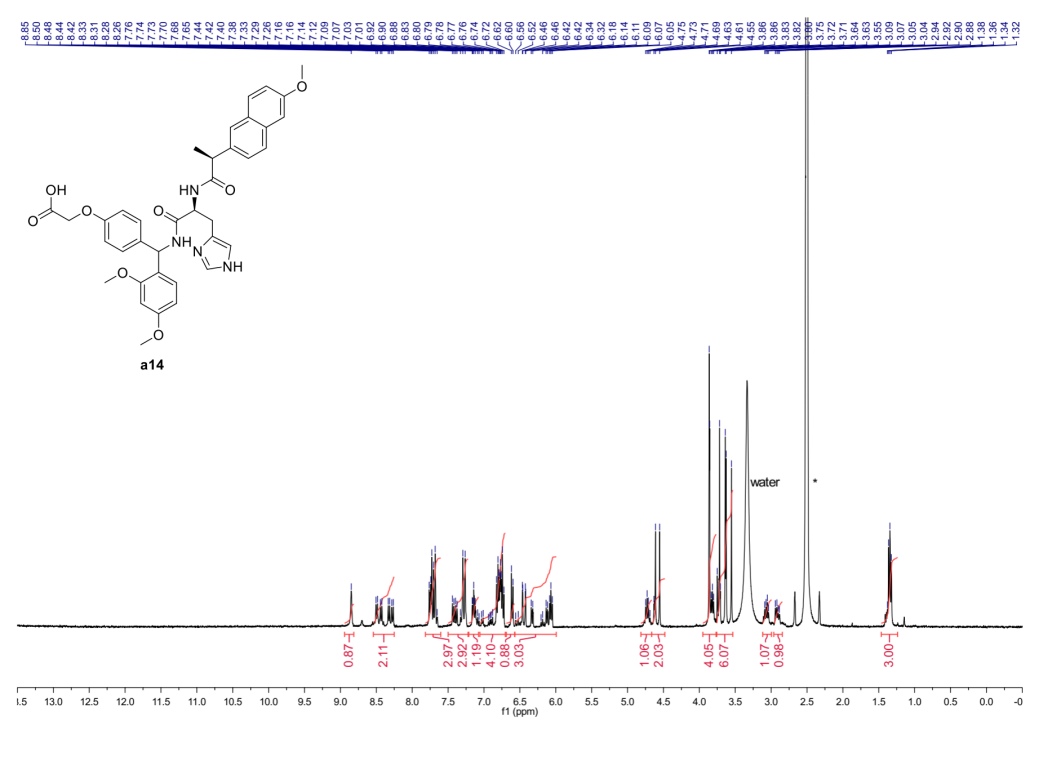


Figure **S30.** ^1^H-NMR spectrum of compound **a14** in DMSO-d_6_.


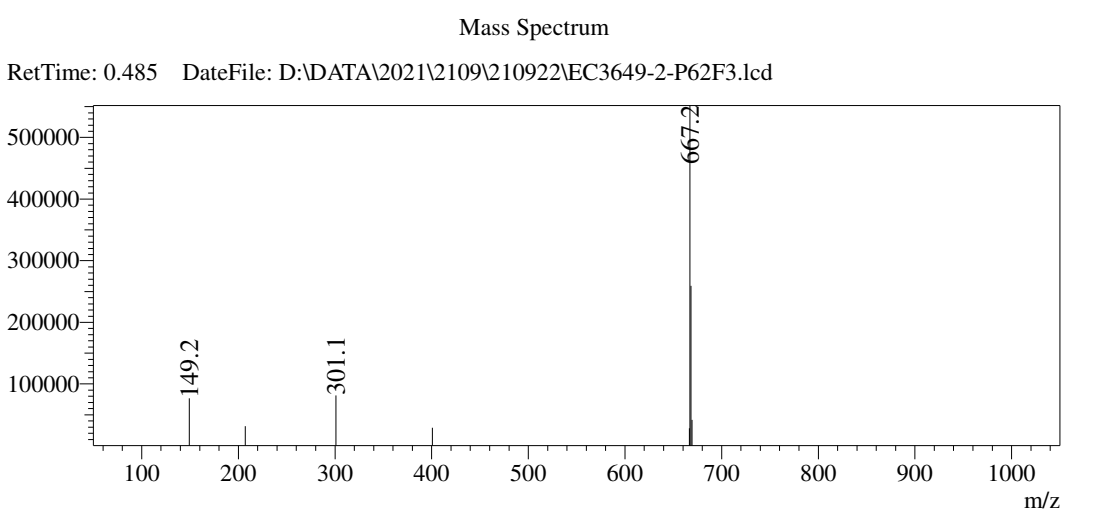


Figure **S31.** ESI mass spectrum of compound **a14**.


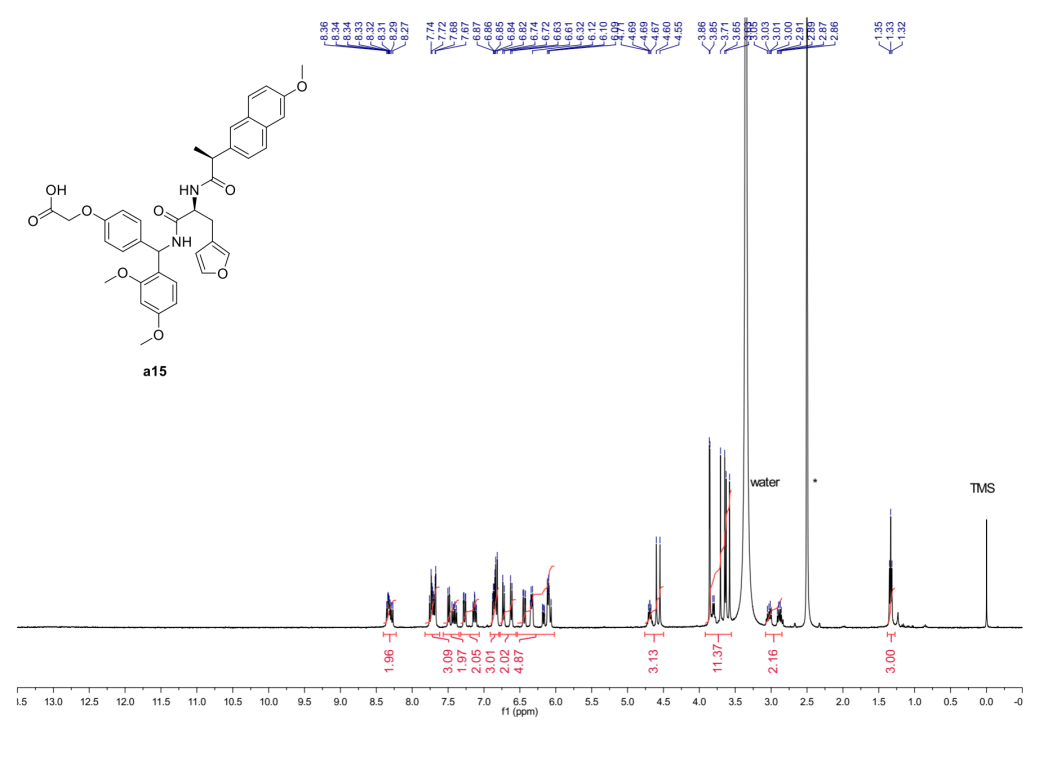


Figure **S32.** ^1^H-NMR spectrum of compound **a15** in DMSO-d_6_.

Figure **S33.** ESI mass spectrum of compound **a15**.


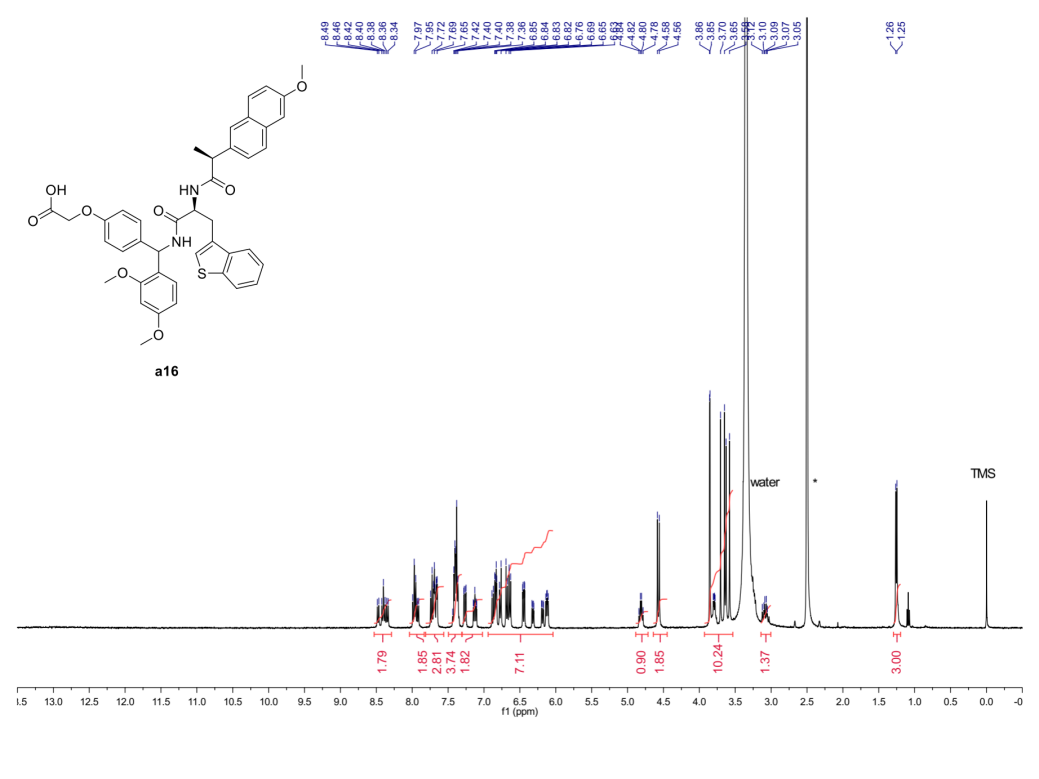


Figure **S34.** ^1^H-NMR spectrum of compound **a16** in DMSO-d_6_.


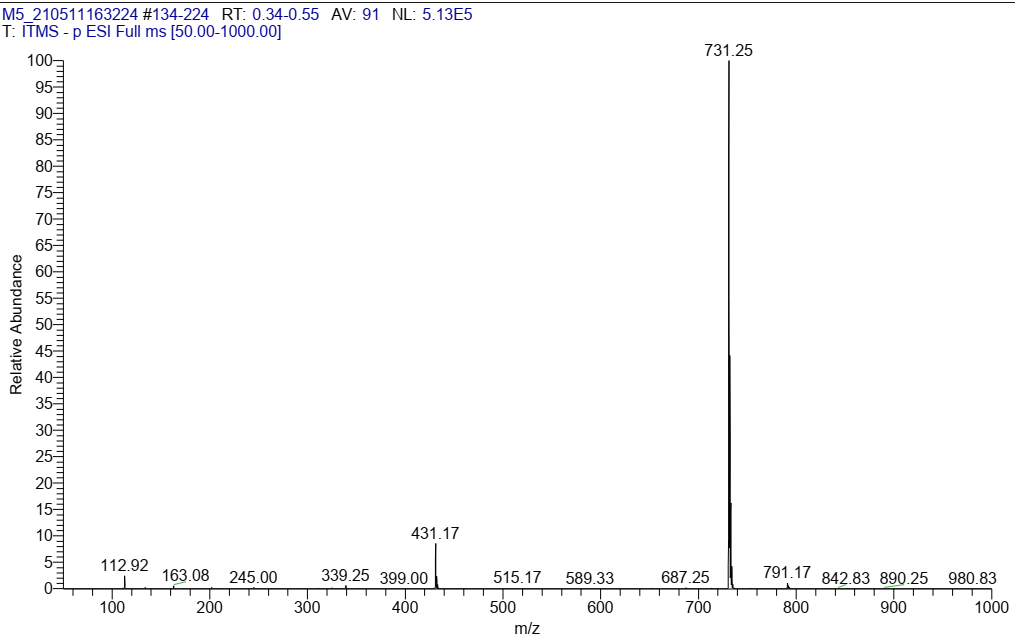


Figure **S35.** ESI mass spectrum of compound **a16**.


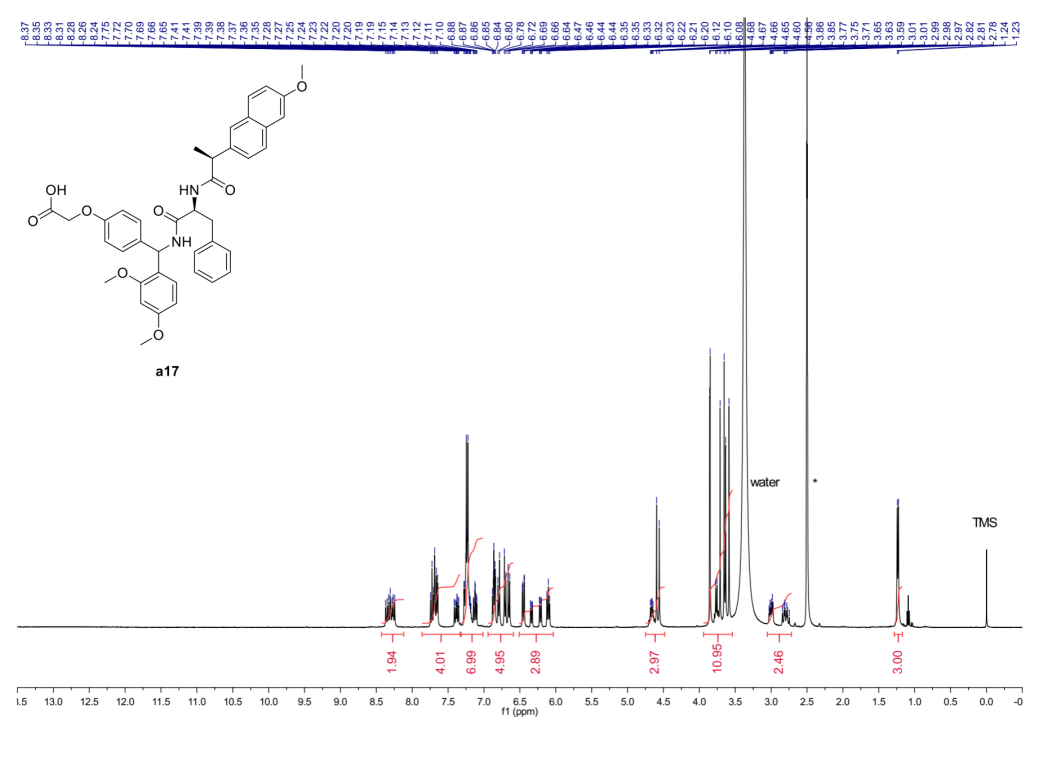


Figure **S36.** ^1^H-NMR spectrum of compound **a17** in DMSO-d_6_.


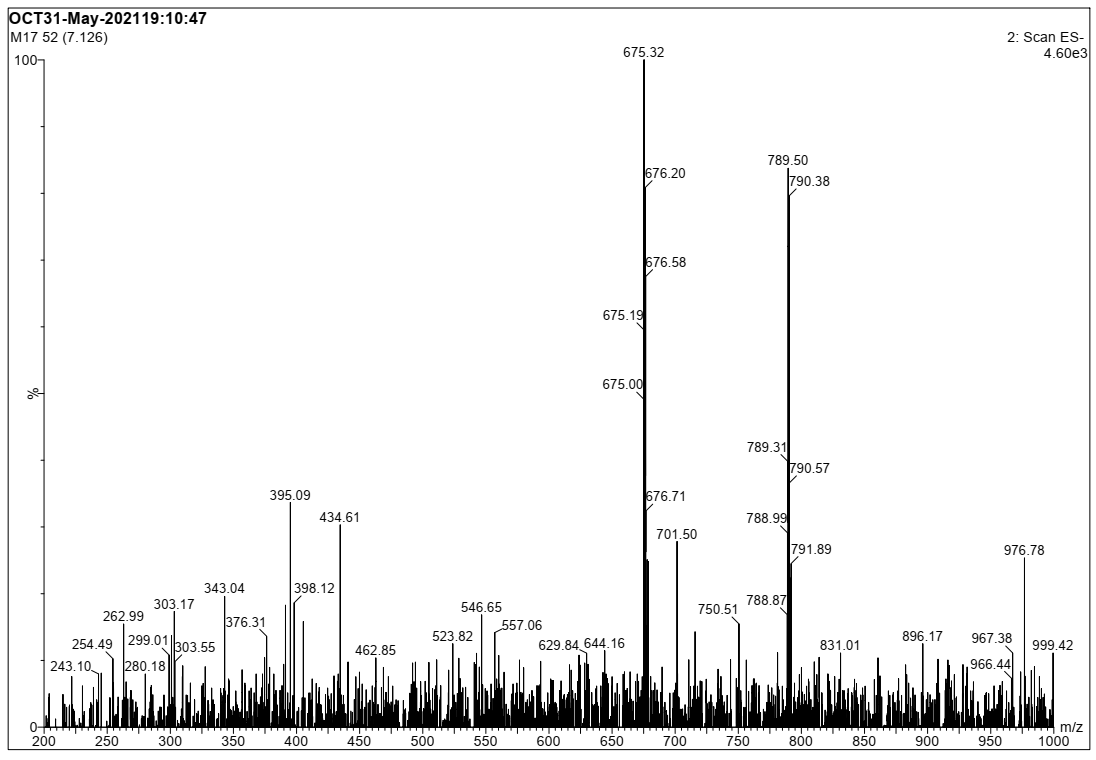


Figure **S37.** ESI mass spectrum of compound **a17**.


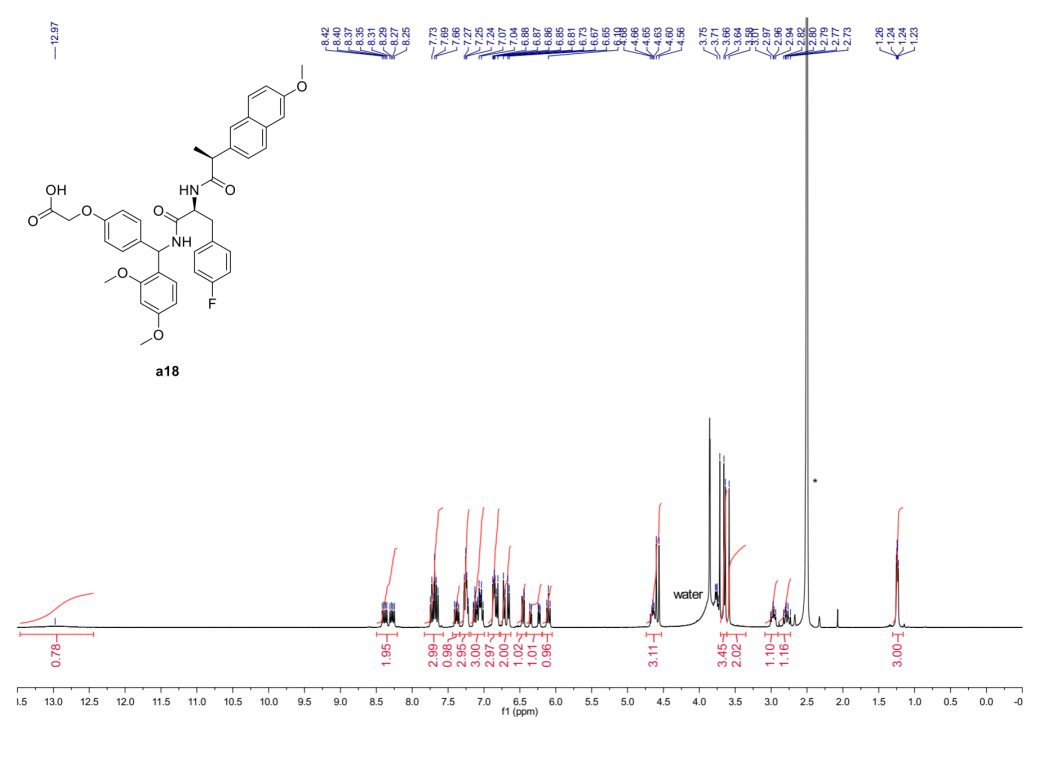


Figure **S38.** ^1^H-NMR spectrum of compound **a18** in DMSO-d_6_.


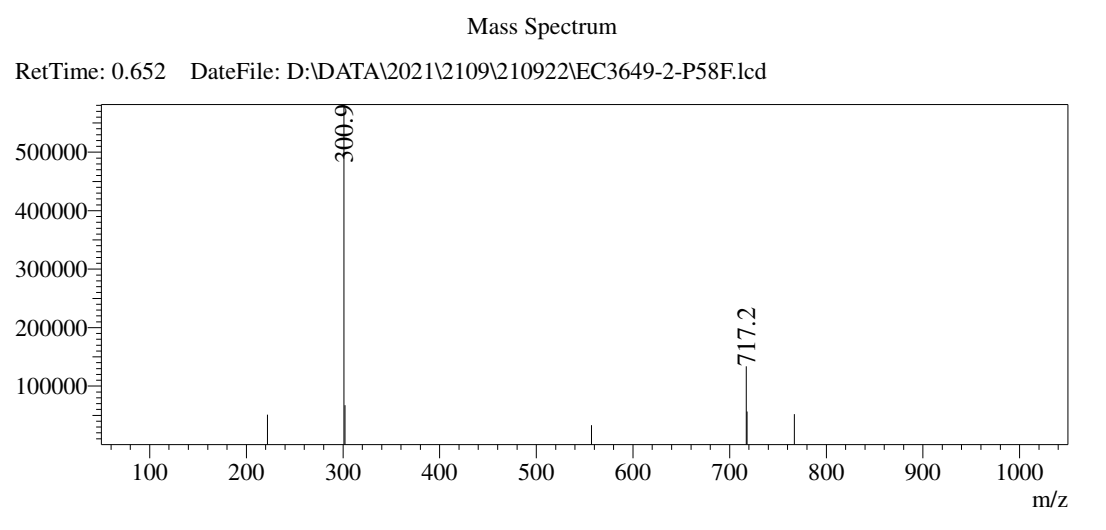


Figure **S39.** ESI mass spectrum of compound **a18**.


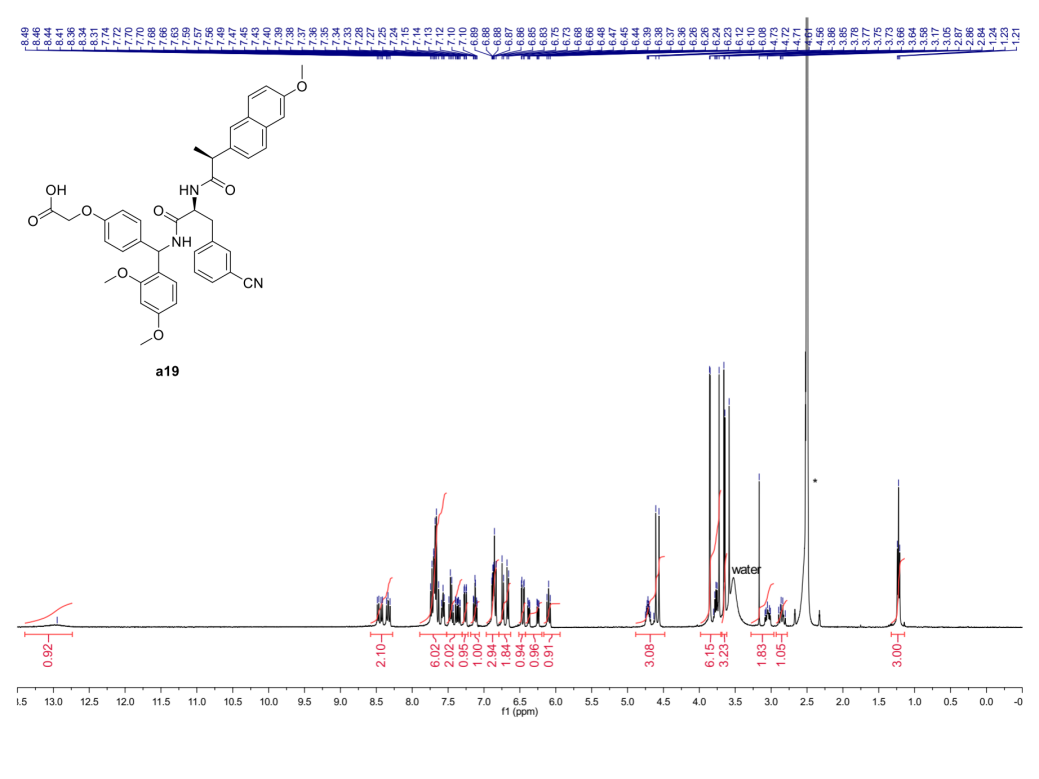


Figure **S40.** ^1^H-NMR spectrum of compound **a19** in DMSO-d_6_.


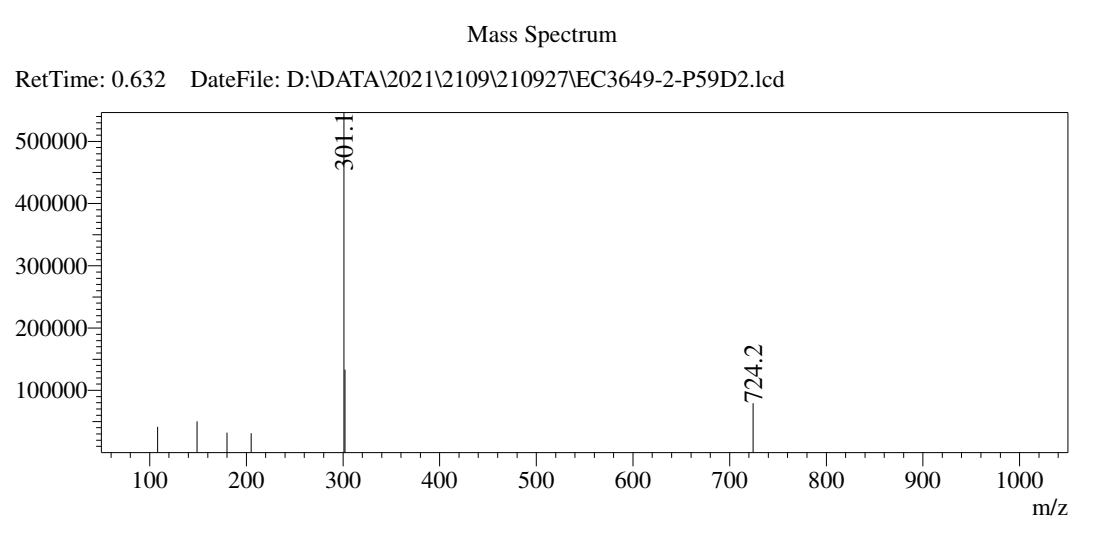


Figure **S41.** ESI mass spectrum of compound **a19**.


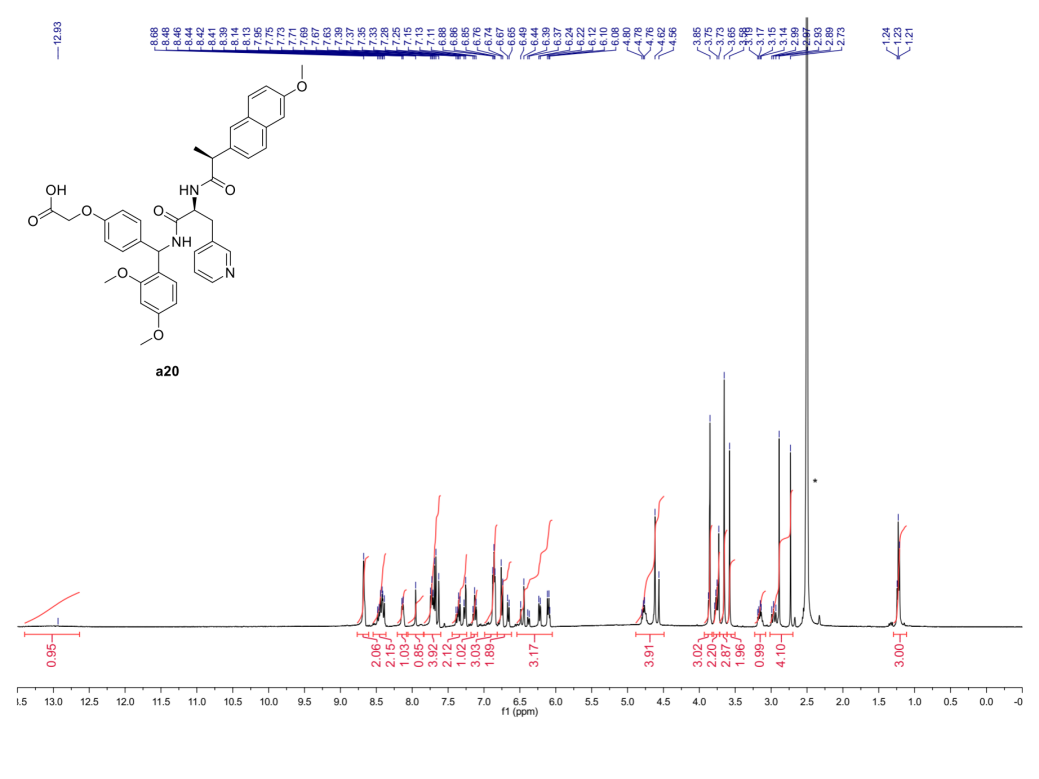


Figure **S42.** ^1^H-NMR spectrum of compound **a20** in DMSO-d_6_.


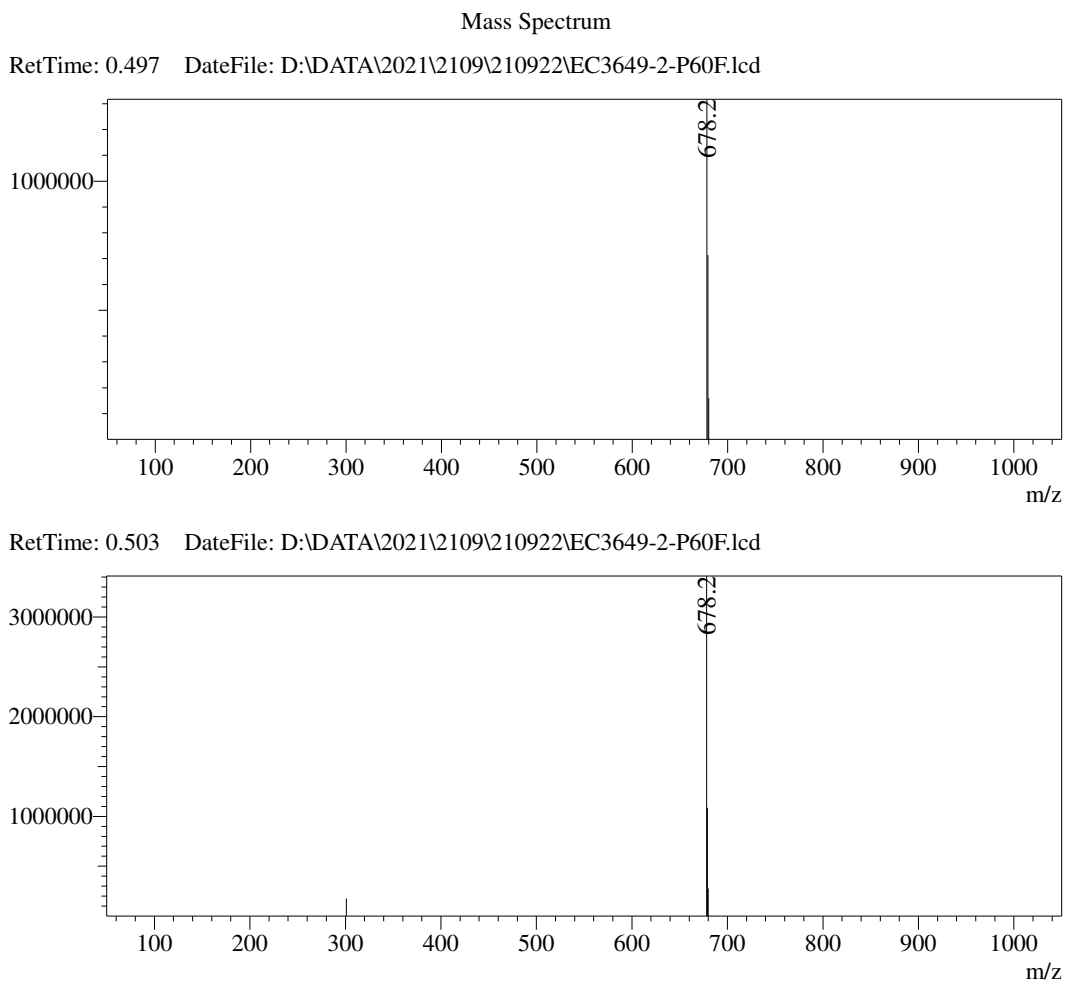


Figure **S43.** ESI mass spectrum of compound **a20**.


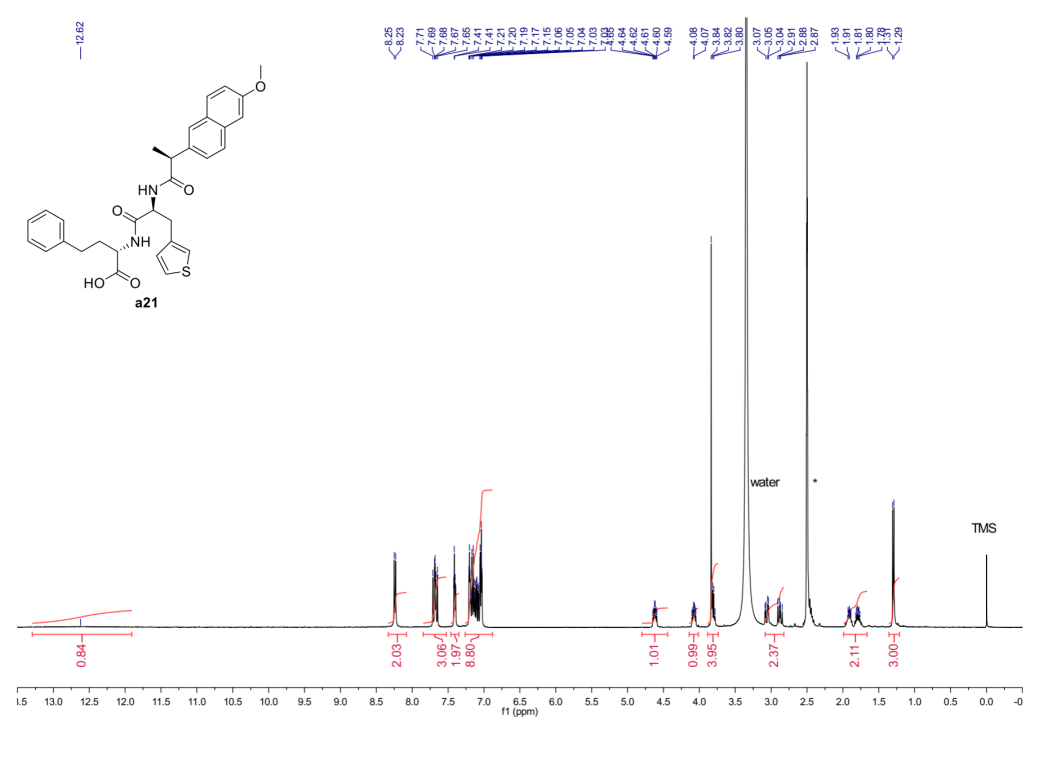


Figure **S44.** ^1^H-NMR spectrum of compound **a21** in DMSO-d_6_.


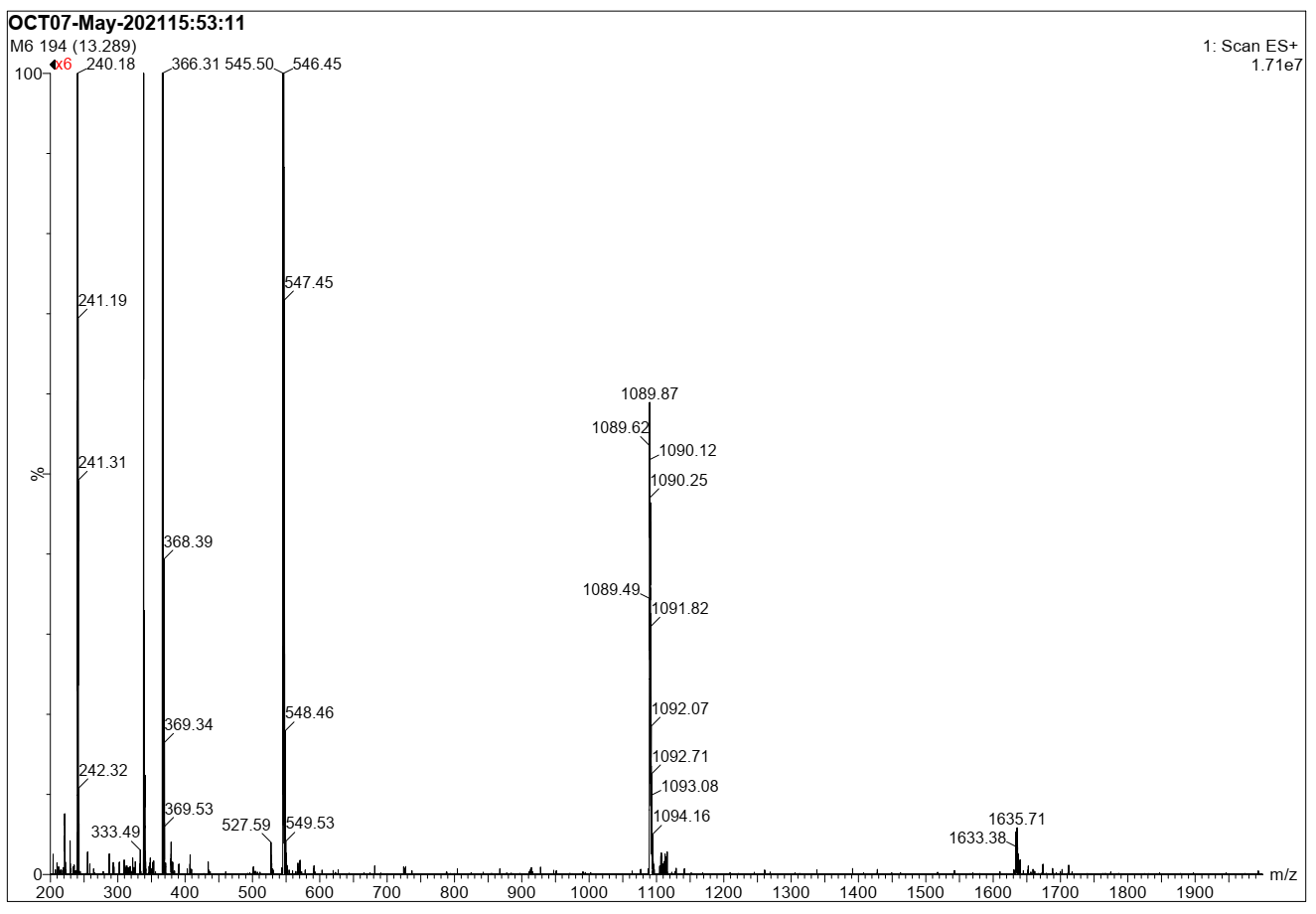


Figure **S45.** ESI mass spectrum of compound **a21**.


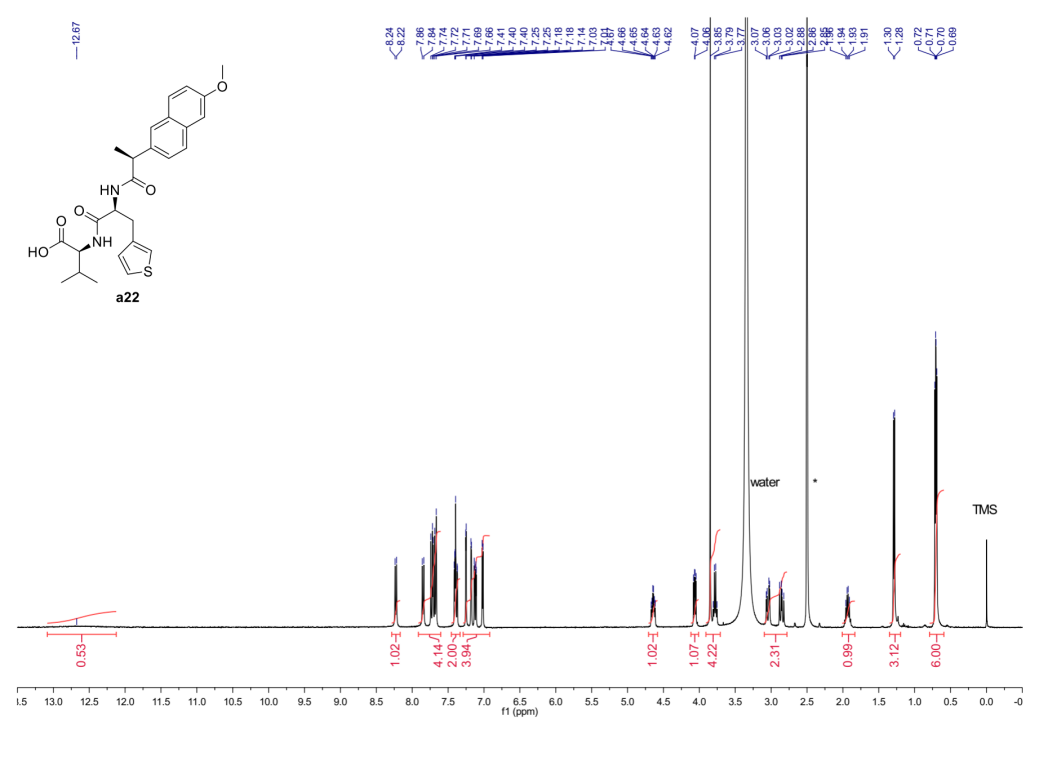


Figure **S46.** ^1^H-NMR spectrum of compound **a22** in DMSO-d_6_.


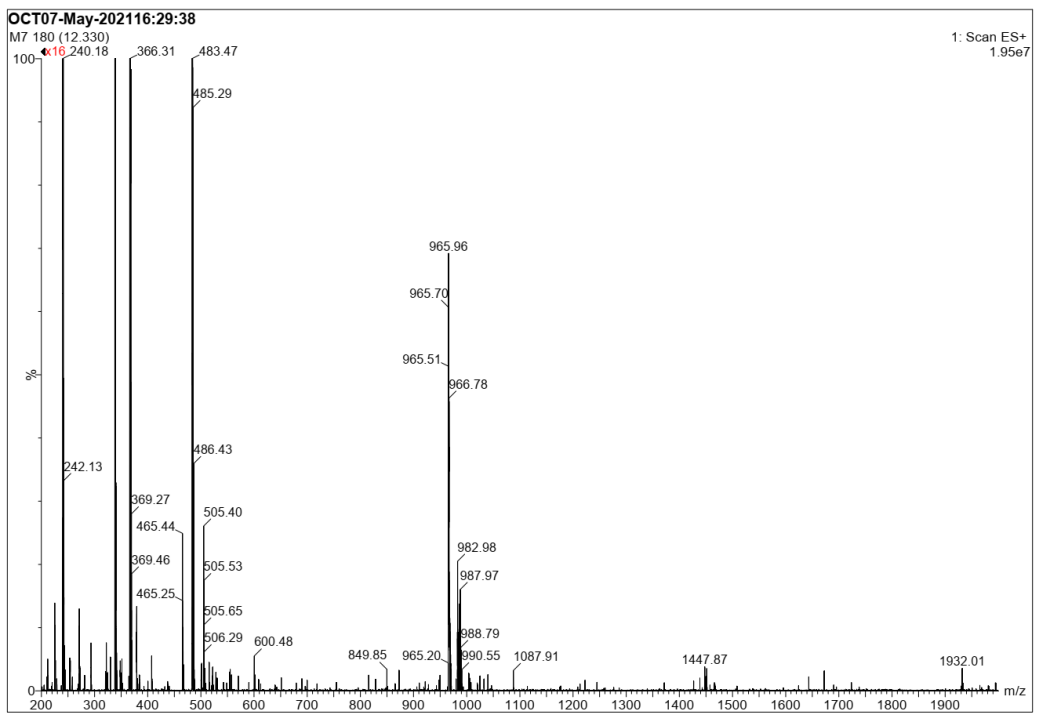


Figure **S47.** ESI mass spectrum of compound **a22**.


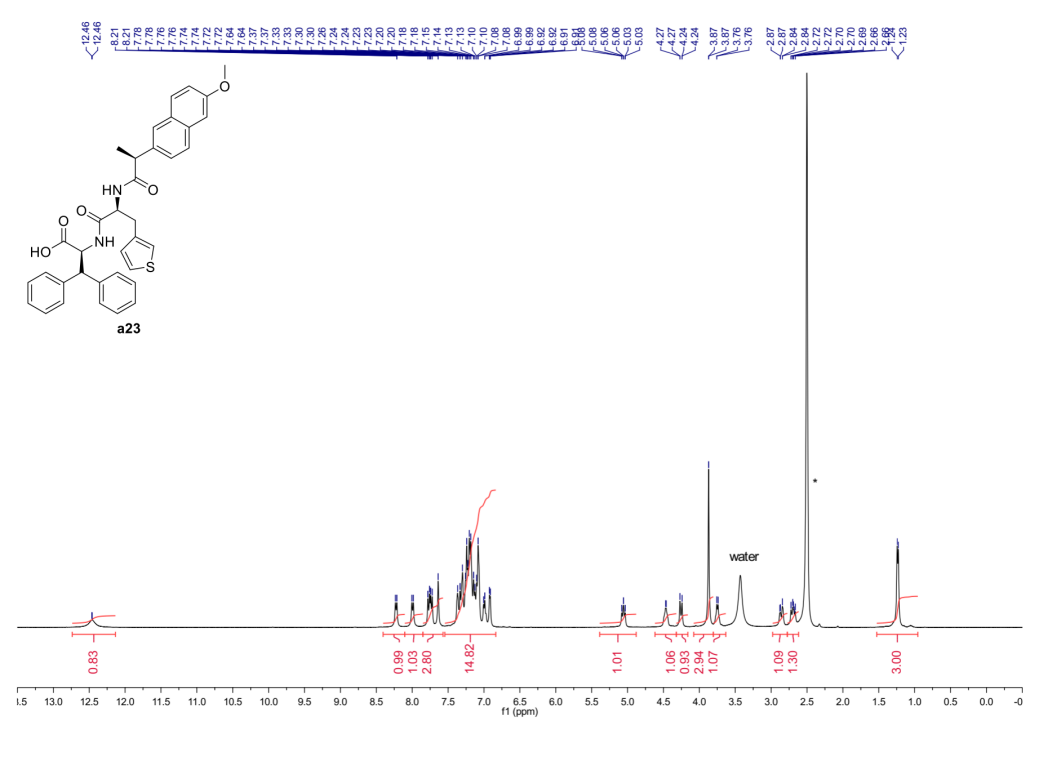


Figure **S48.** ^1^H-NMR spectrum of compound **a23** in DMSO-d_6_.


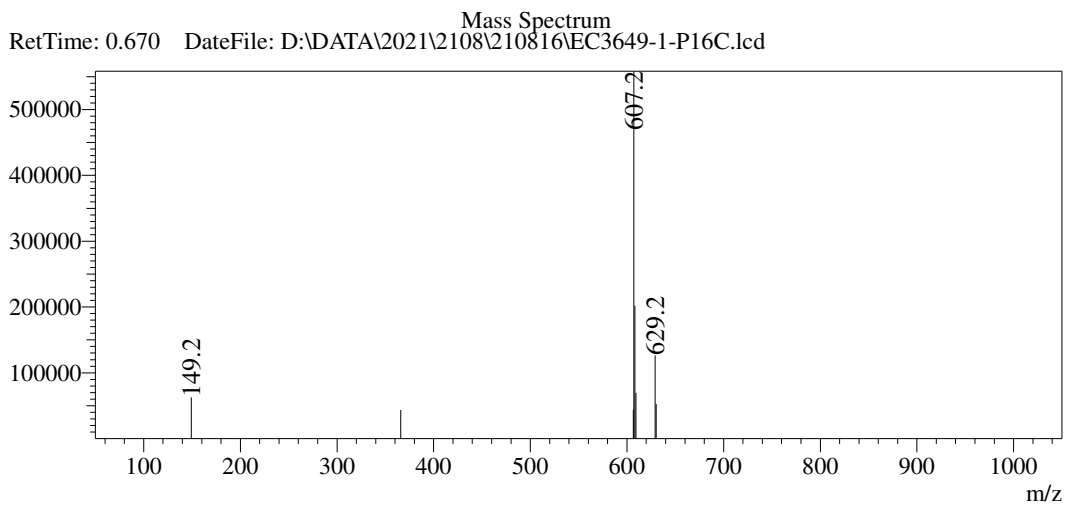


Figure **S49.** ESI mass spectrum of compound **a23**.


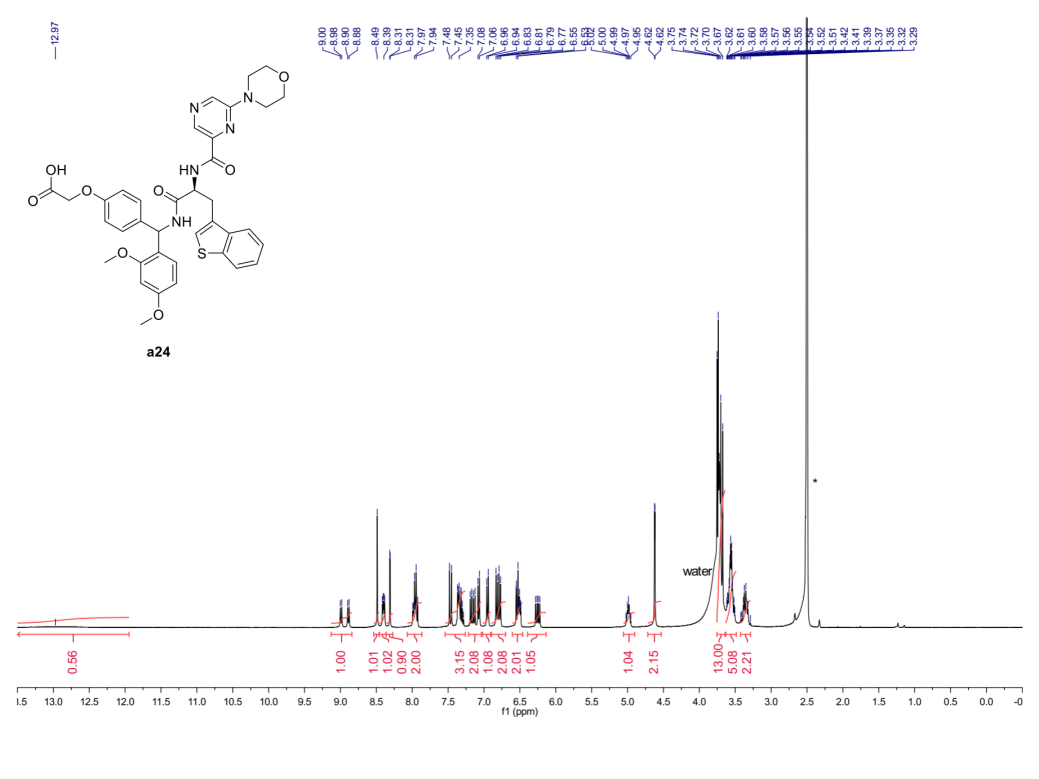


Figure **S50.** ^1^H-NMR spectrum of compound **a24** in DMSO-d_6_.


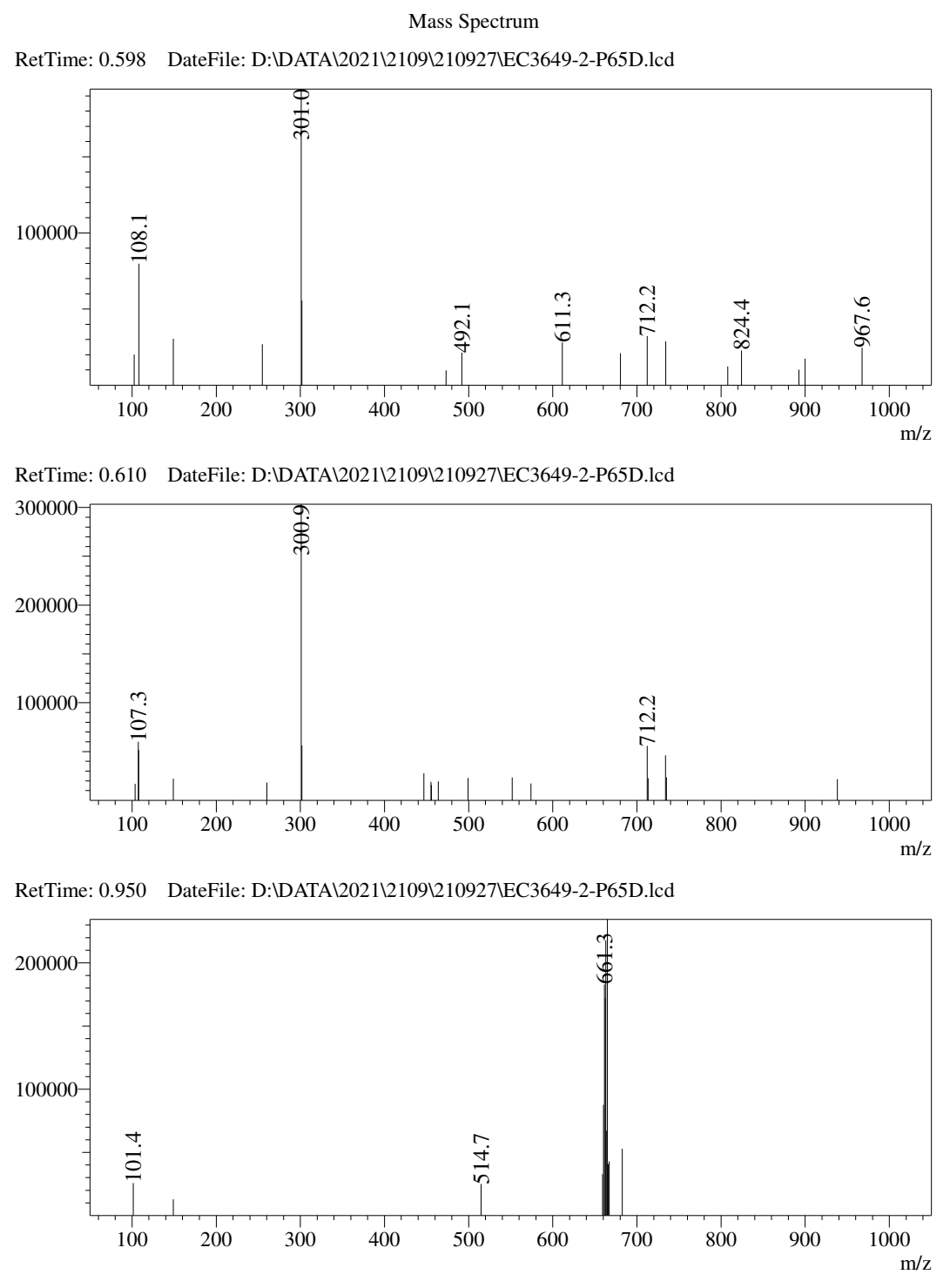


Figure **S51.** ESI mass spectrum of compound **a24**.


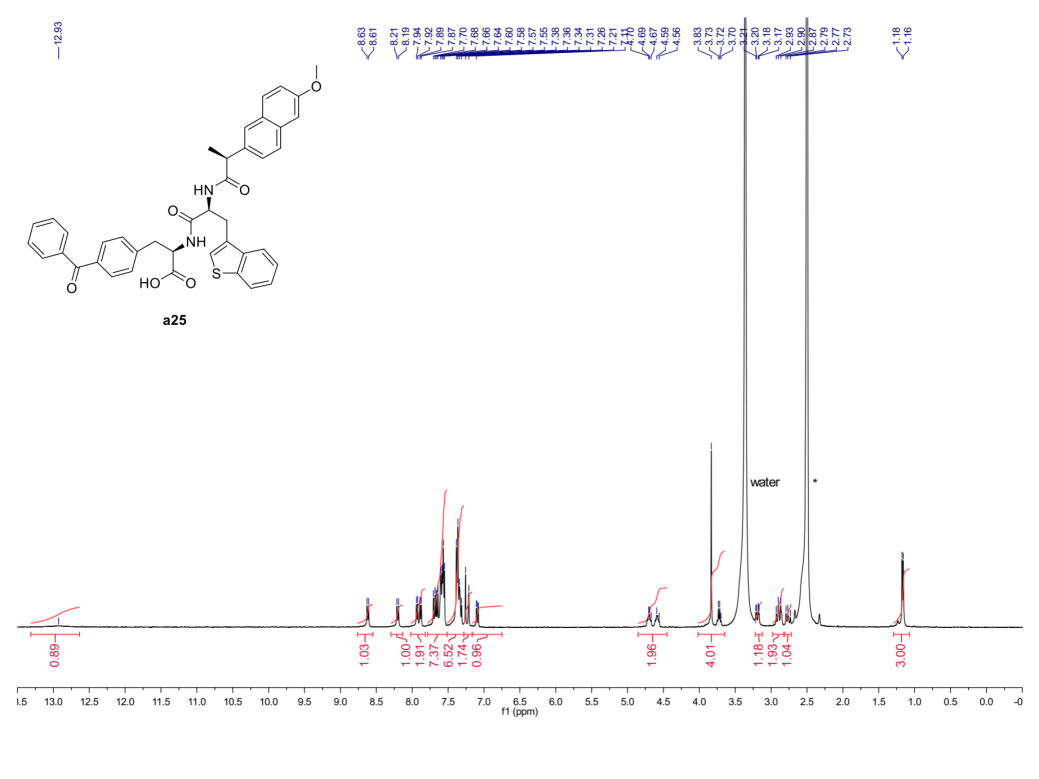


Figure **S52.** ^1^H-NMR spectrum of compound **a25** in DMSO-d_6_.


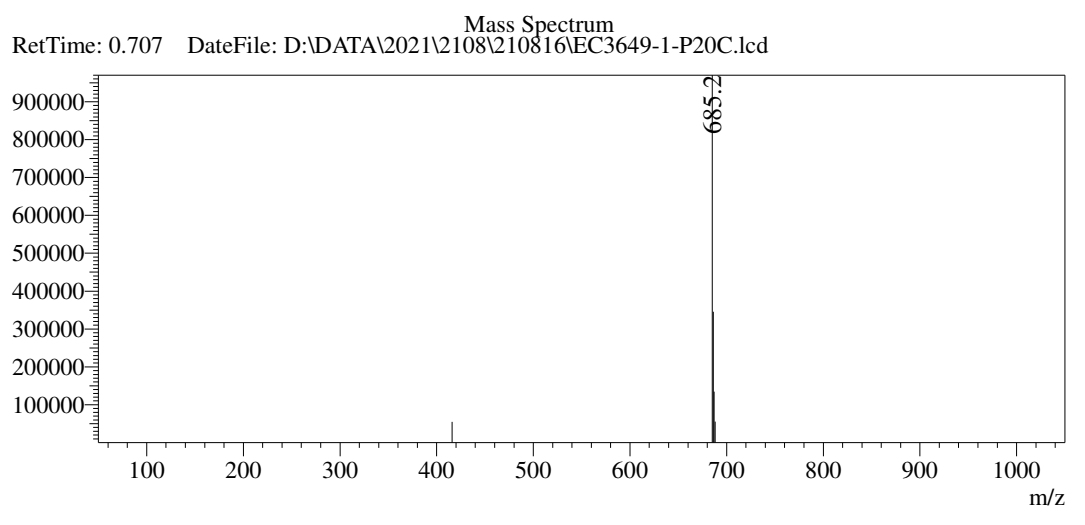


Figure **S53.** ESI mass spectrum of compound **a25**.


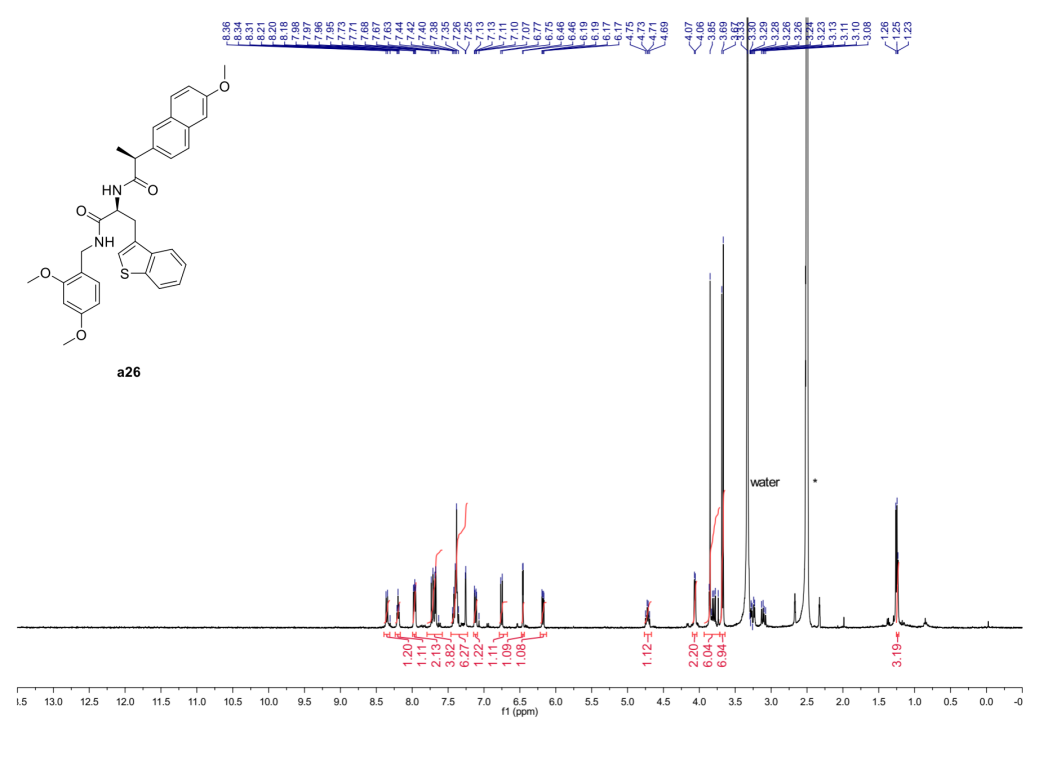


Figure **S54.** ^1^H-NMR spectrum of compound **a26** in DMSO-d_6_.


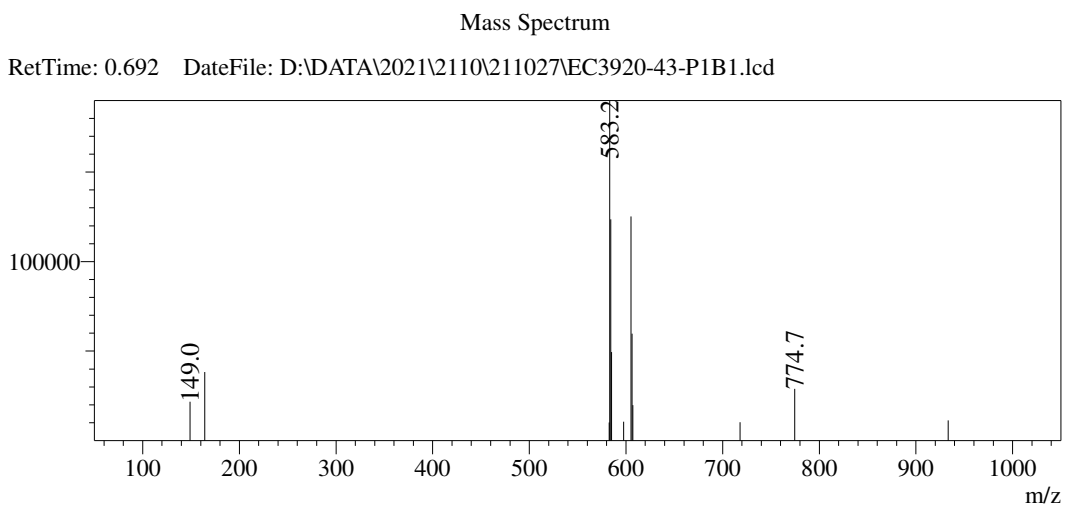


Figure **S55.** ESI mass spectrum of compound **a26**.


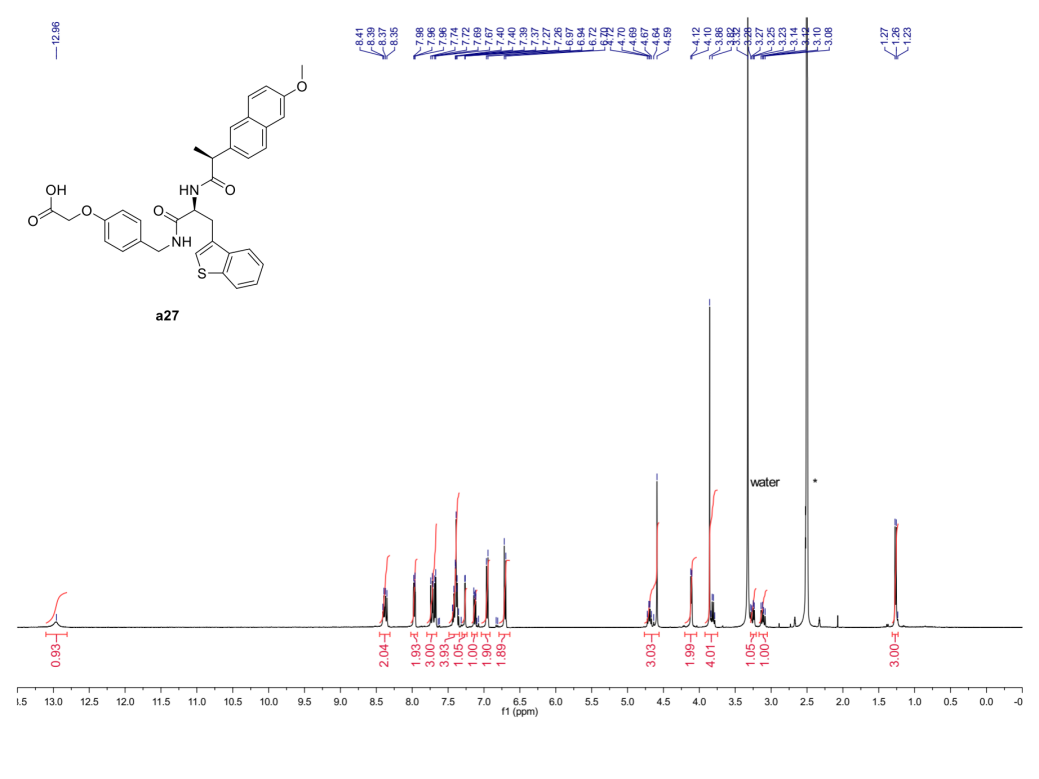


Figure **S56.** ^1^H-NMR spectrum of compound **a27** in DMSO-d_6_.


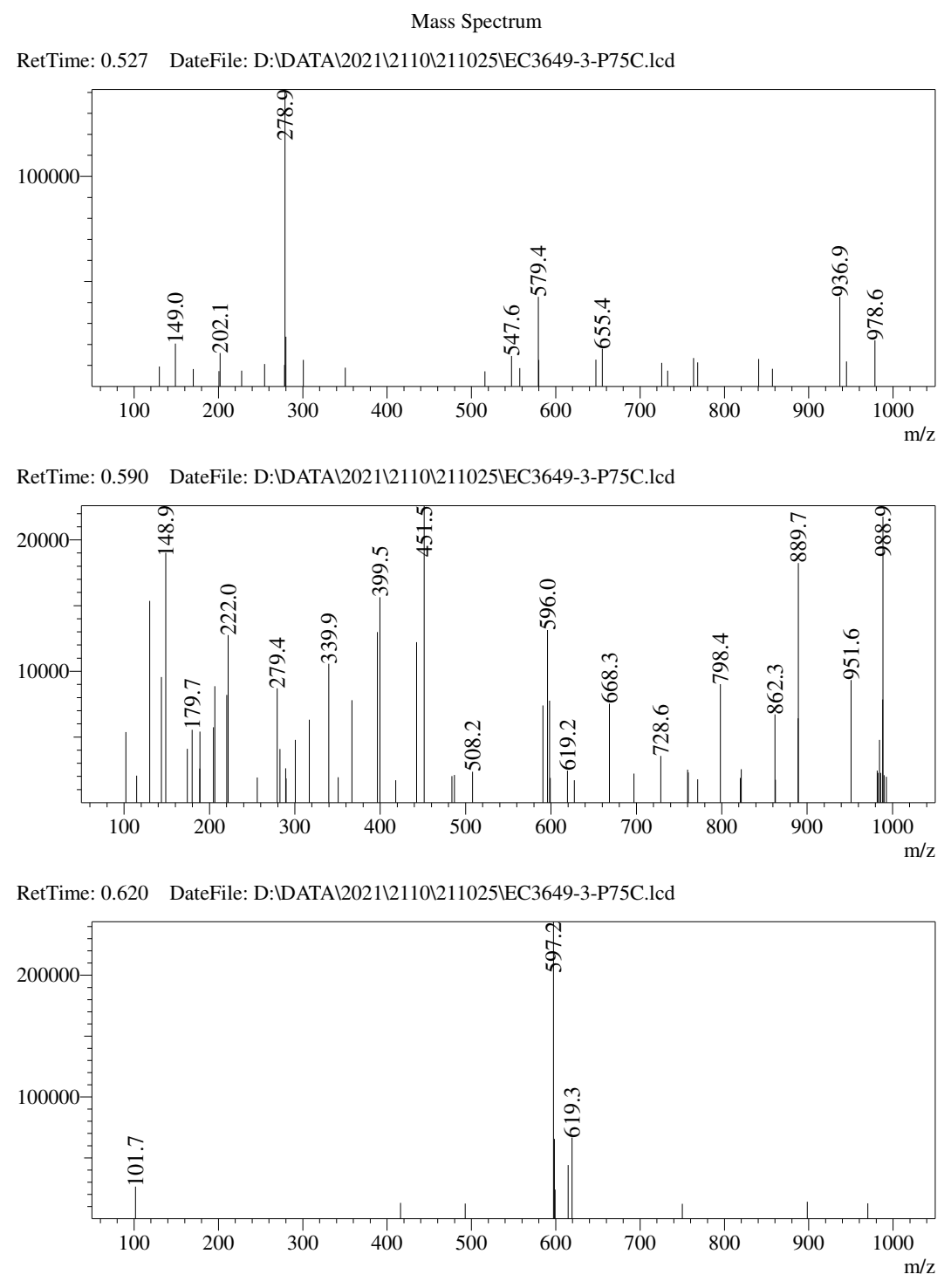


Figure **S57.** ESI mass spectrum of compound **a27**.


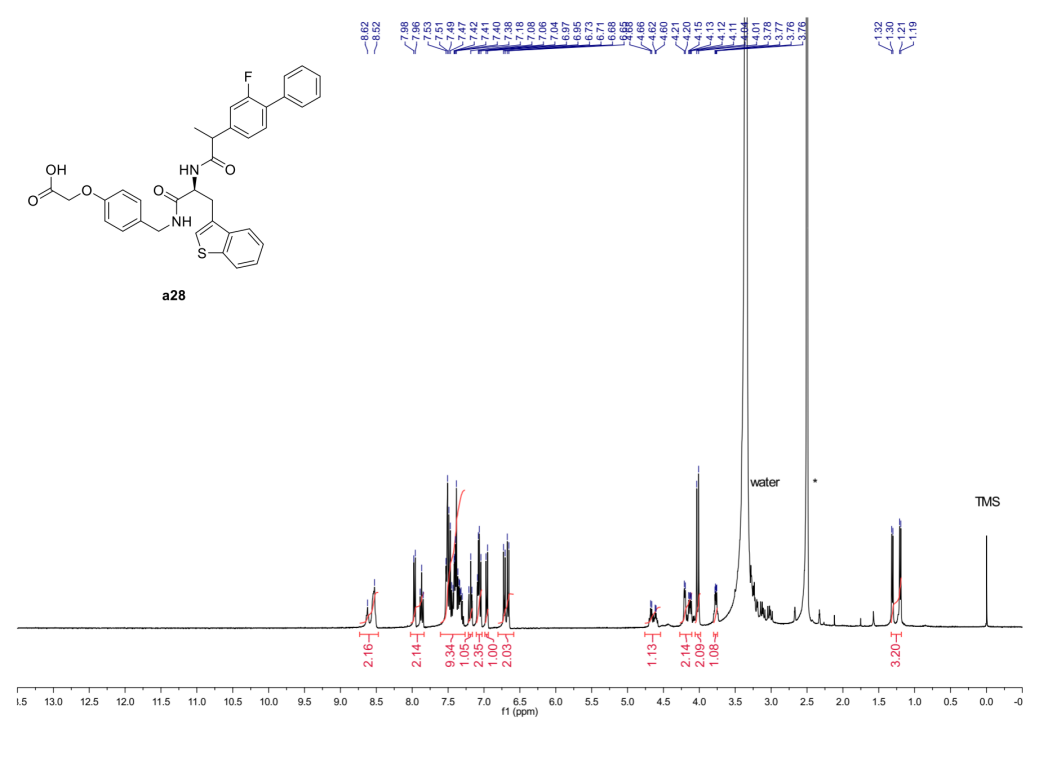


Figure **S58.** ^1^H-NMR spectrum of compound **a28** in DMSO-d_6_.

**
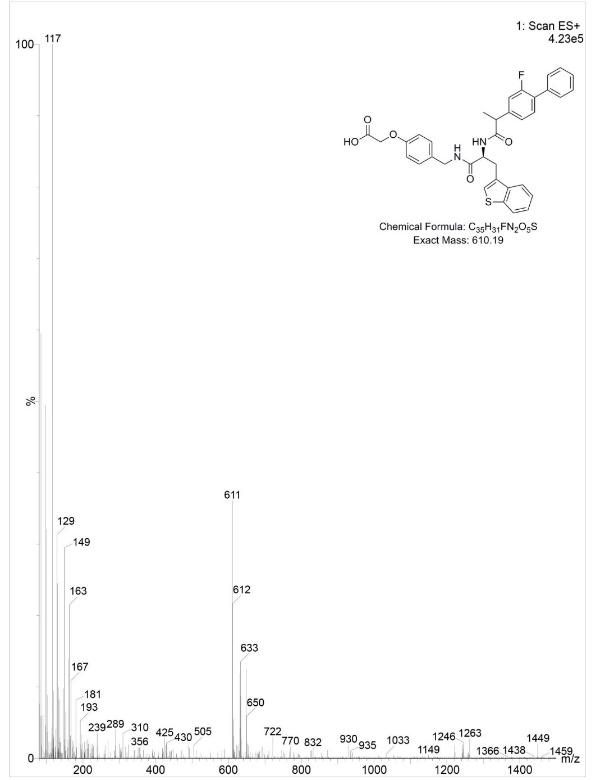
**

Figure **S59.** ESI mass spectrum of compound **a28**.


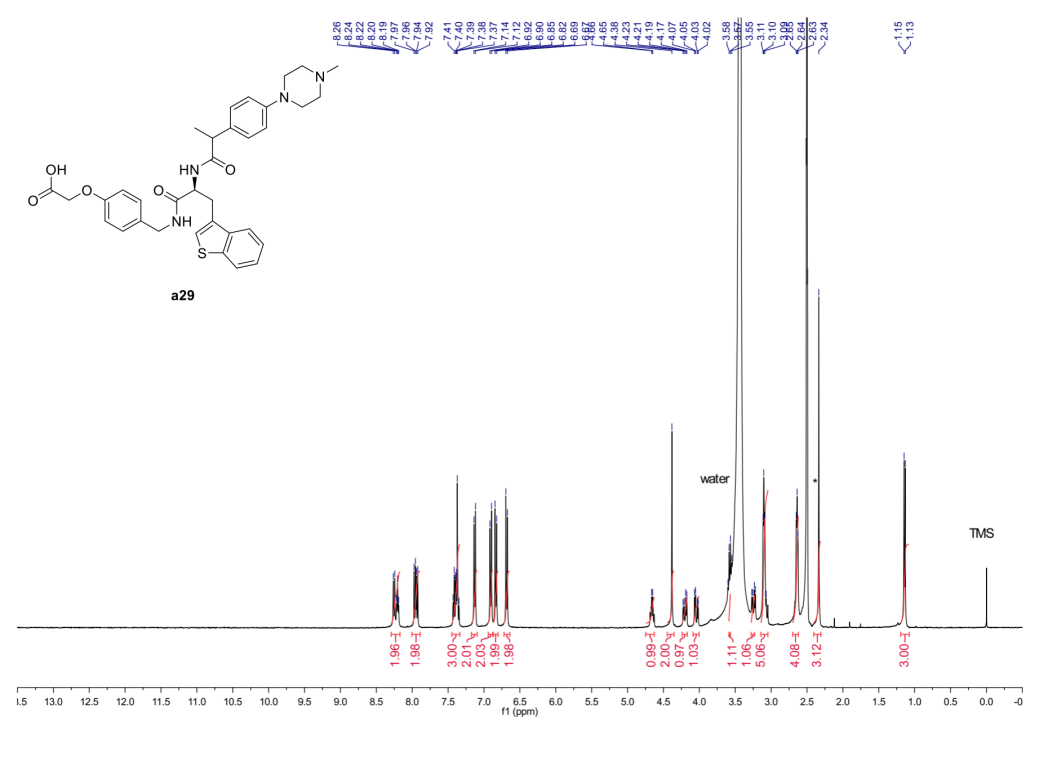


Figure **S60.** ^1^H-NMR spectrum of compound **a29** in DMSO-d_6_.

**
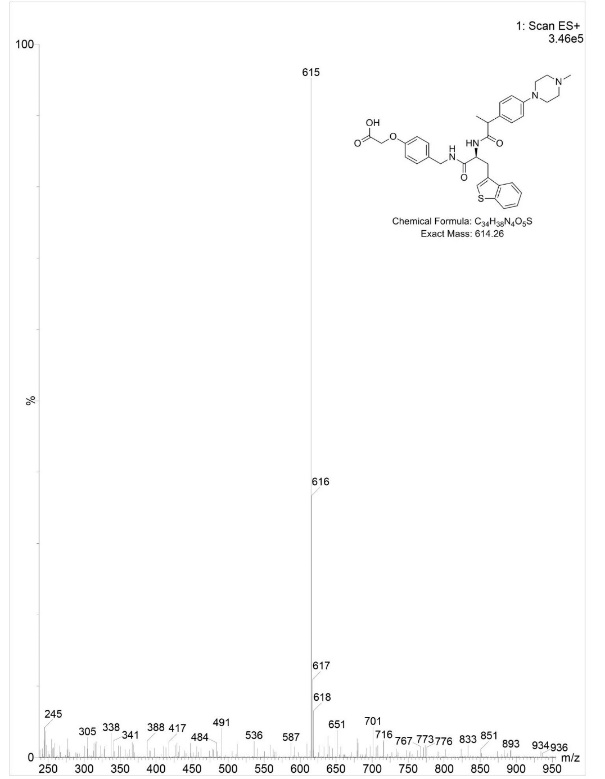
**

Figure **S61.** ESI mass spectrum of compound **a29**.


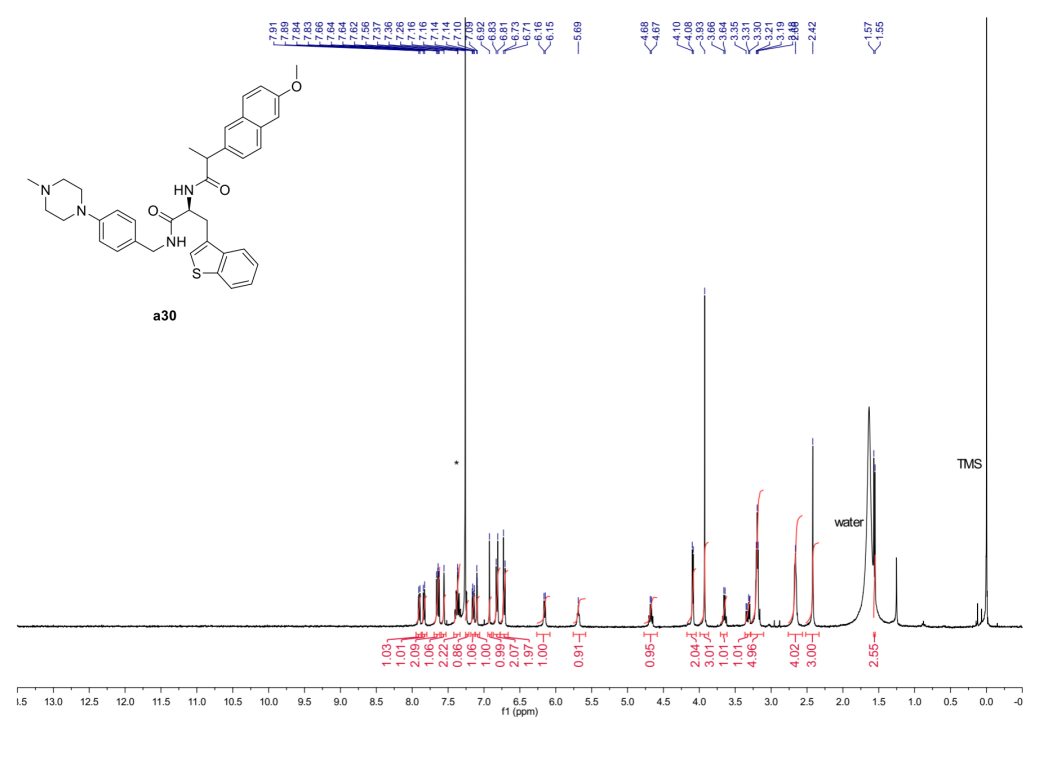


Figure **S62.** ^1^H-NMR spectrum of compound **a30** in CDCl_3_.

**
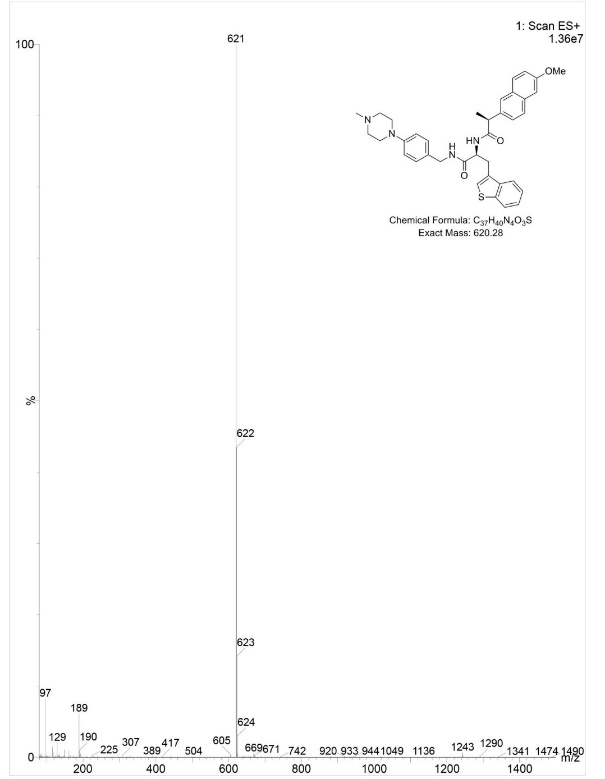
**

Figure **S63.** ESI mass spectrum of compound **a30**.


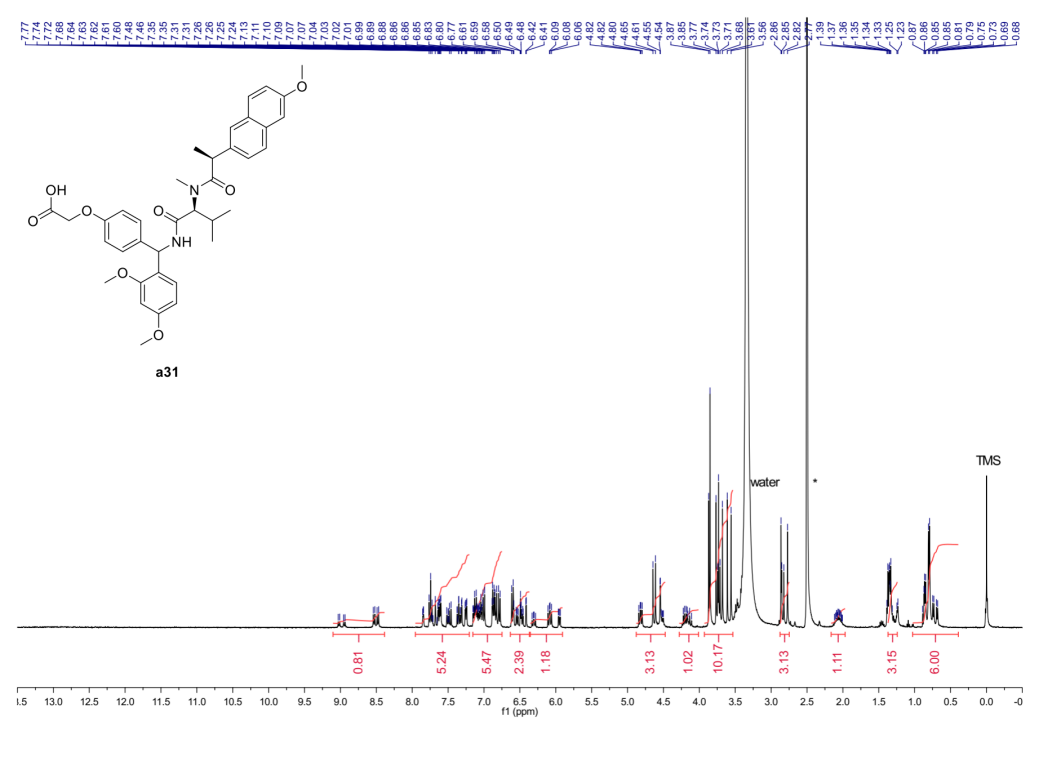


Figure **S64.** ^1^H-NMR spectrum of compound **a31** in DMSO-d_6_.


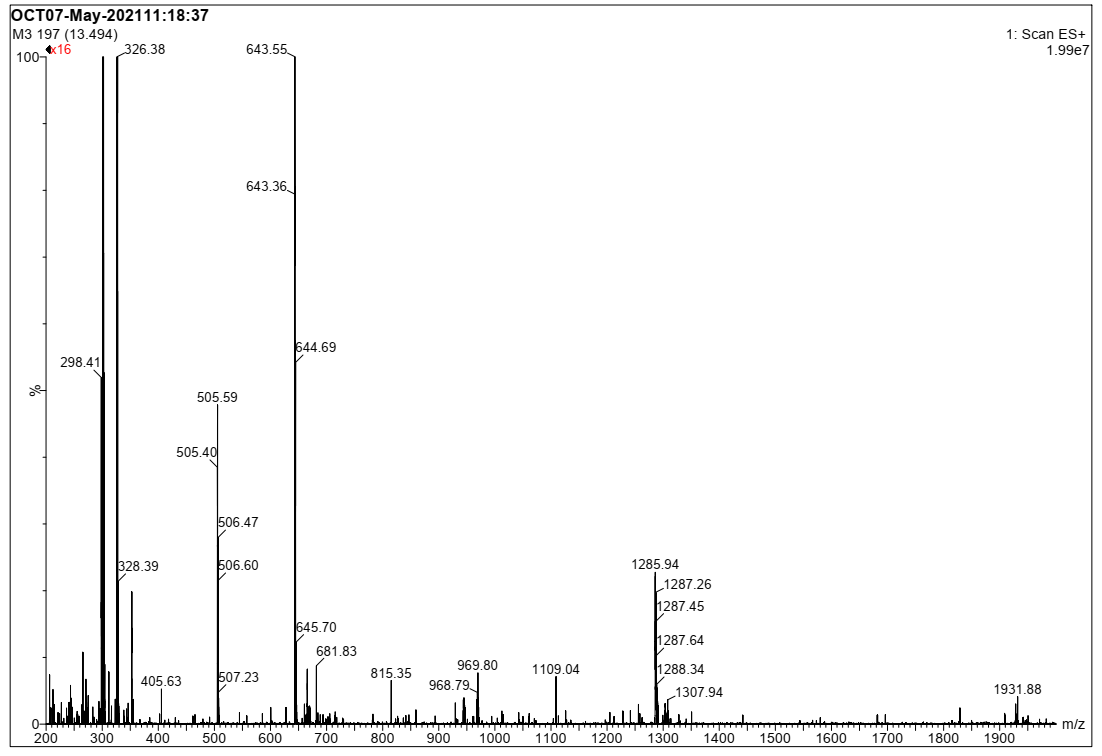


Figure **S65.** ESI mass spectrum of compound **a31**.


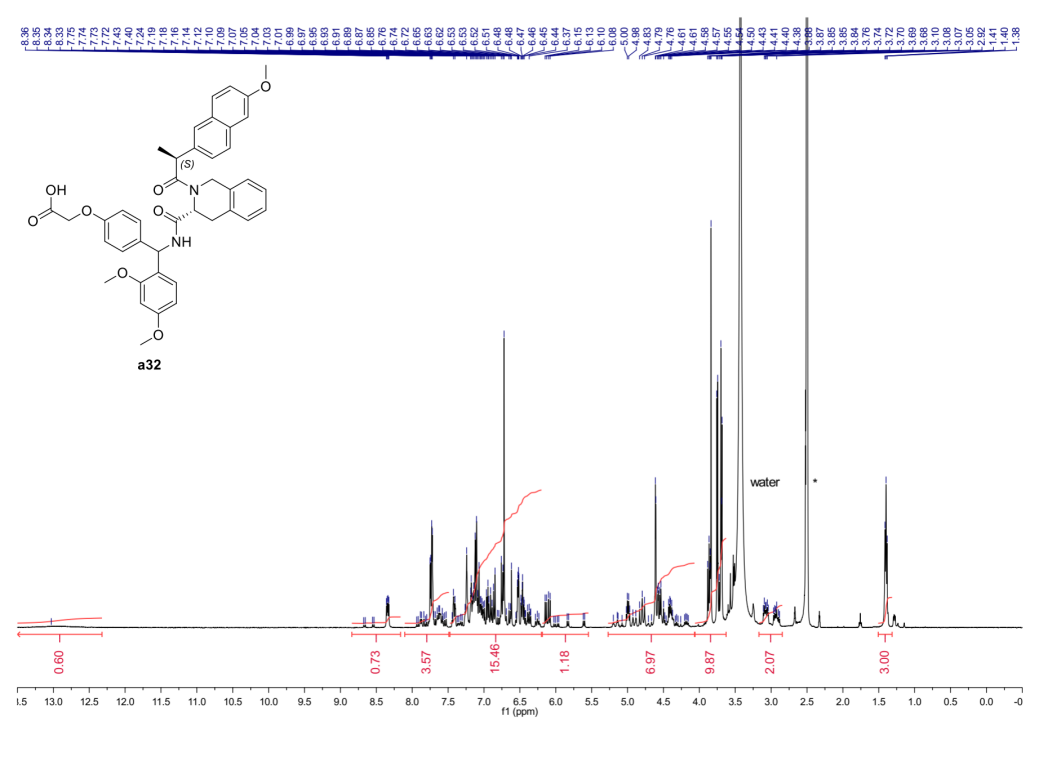


Figure **S66.** ^1^H-NMR spectrum of compound **a32** in DMSO-d_6_.


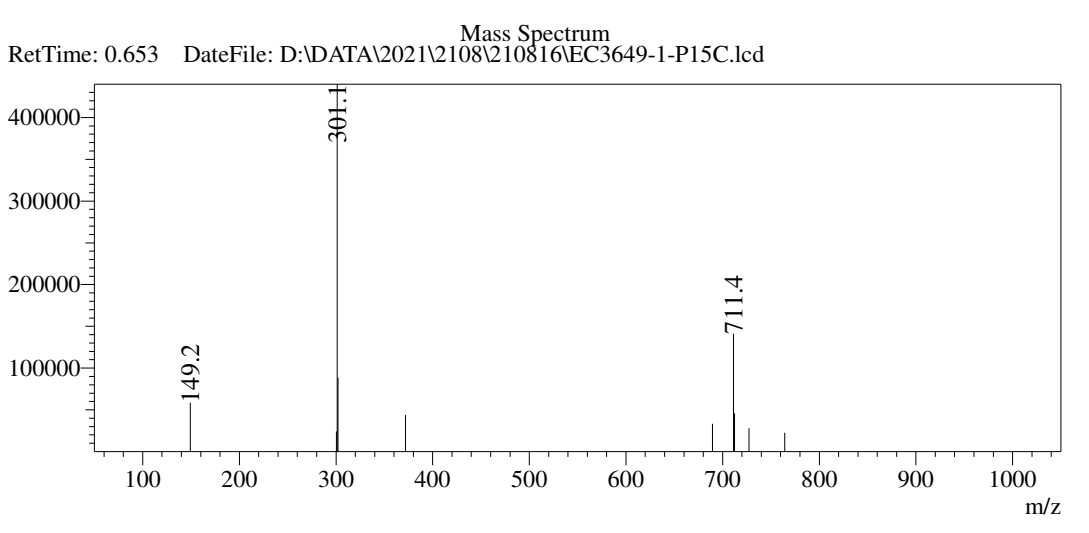


Figure **S67.** ESI mass spectrum of compound **a32**.


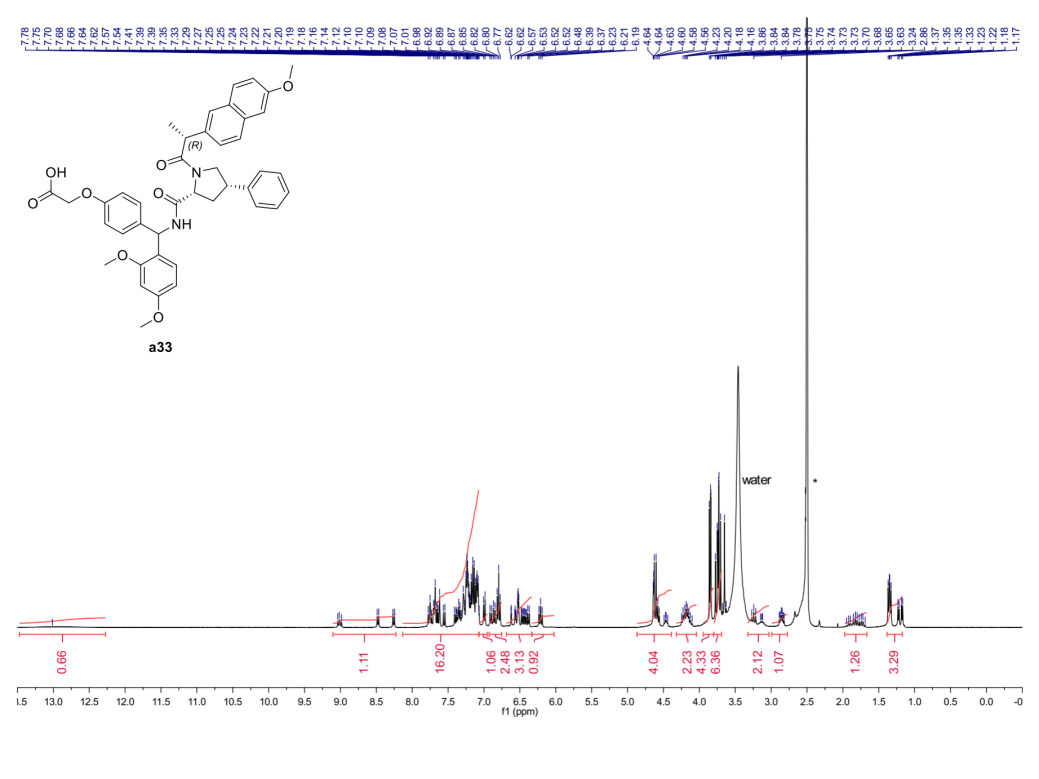


Figure **S68.** ^1^H-NMR spectrum of compound **a33** in DMSO-d_6_.


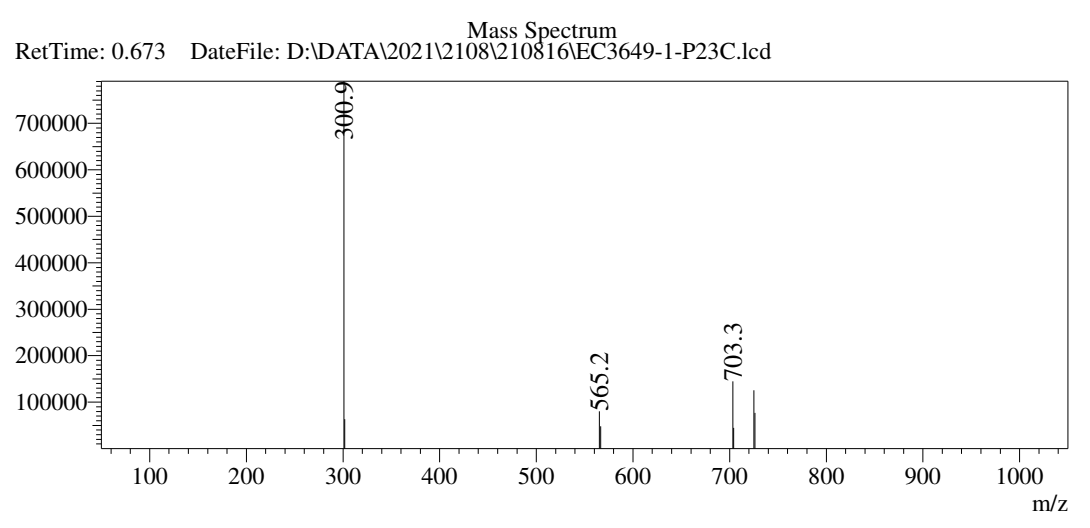


Figure **S69.** ESI mass spectrum of compound **a33**.


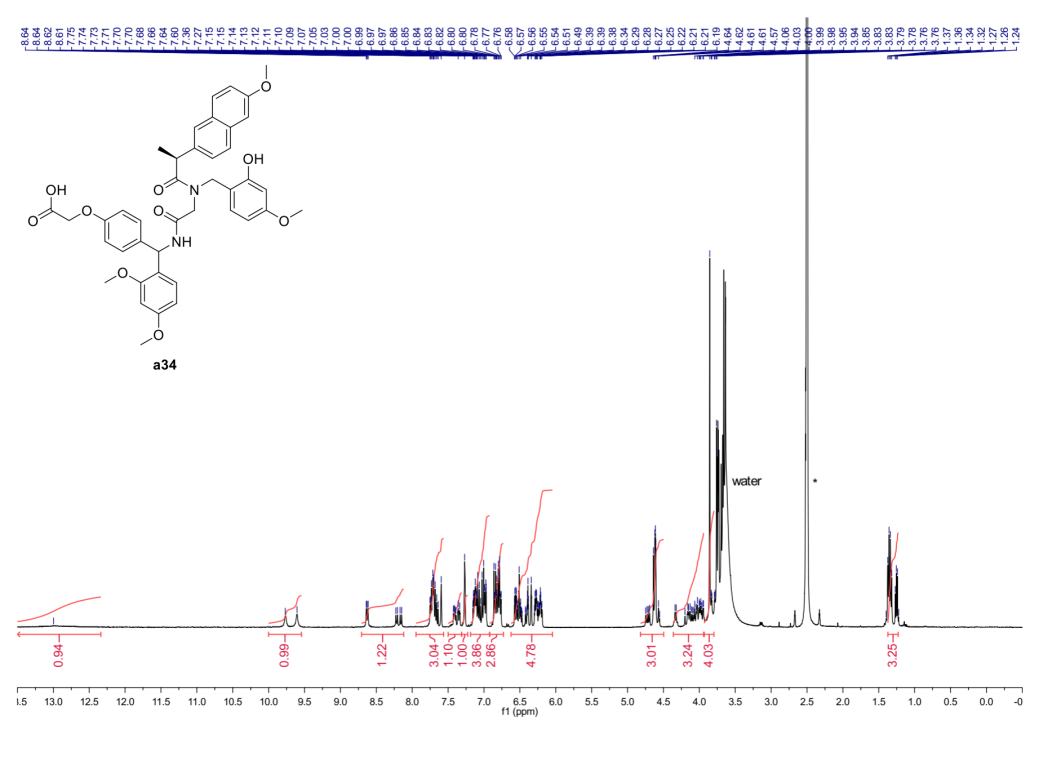


Figure **S70.** ^1^H-NMR spectrum of compound **a34** in DMSO-d_6_.


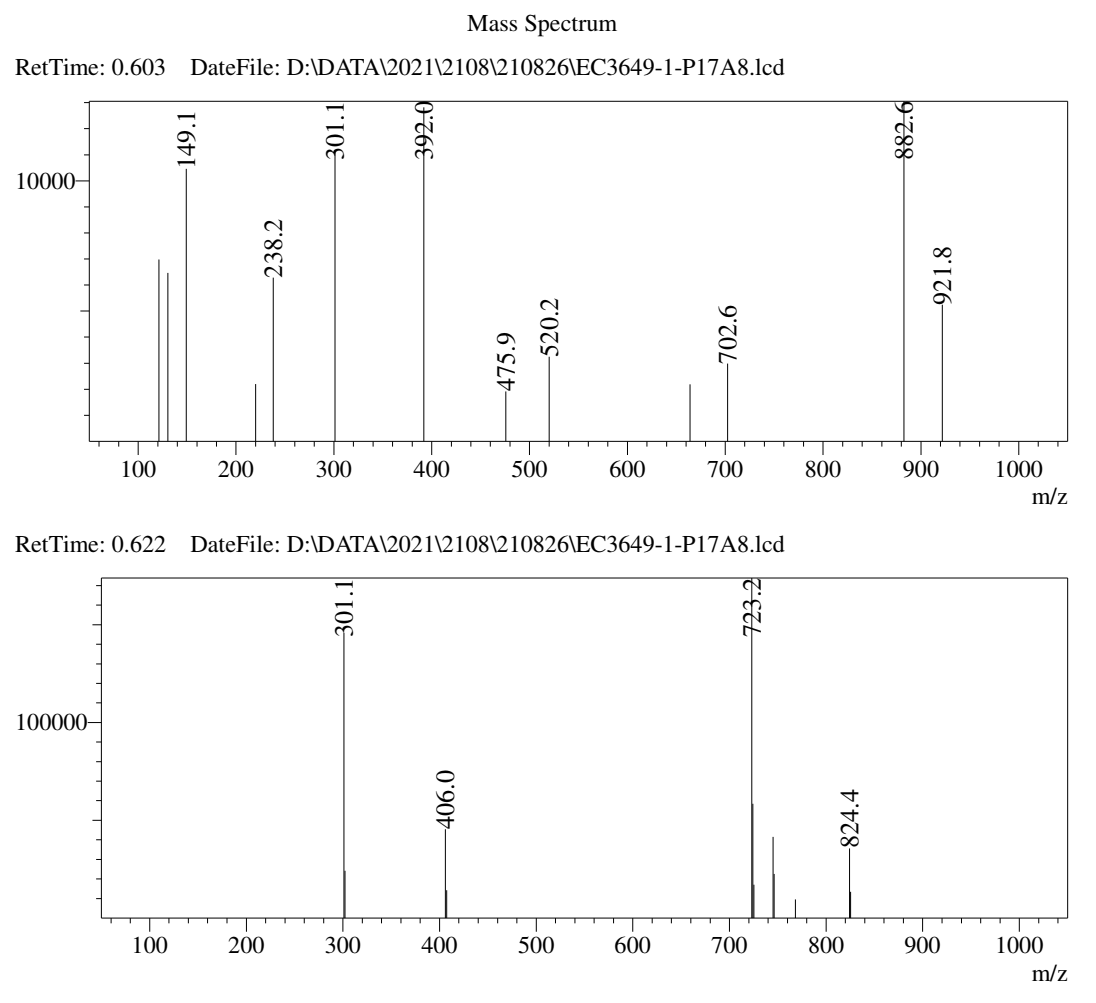


Figure **S71.** ESI mass spectrum of compound **a34**.
